# Ligand-Free Cerium-Catalyzed Decarboxylative Fluorination of Carboxylic Acids

**DOI:** 10.1021/acsorginorgau.5c00024

**Published:** 2025-04-23

**Authors:** Maham Azhar, Tianyou Peng, Osama El-Sepelgy

**Affiliations:** 28392 Leibniz Institute for Catalysis e.V., Albert-Einstein-Str. 29a, 18059 Rostock, Germany

**Keywords:** light, excited-state, cerium, fluorine, decarboxylation

## Abstract

We report a ligand-free, cerium-catalyzed decarboxylative fluorination of carboxylic acids via photoinduced ligand-to-metal charge transfer (LMCT) catalysis. This method utilizes readily available carboxylic acids as radical precursors, enabling the selective formation of alkyl fluorides under mild conditions. The protocol tolerates diverse carboxylic acids with a high functional group tolerance. Mechanistic studies confirm that the reaction proceeds via alkyl radical generation through light-induced LMCT of cerium­(IV) carboxylate followed by fluorine transfer. This efficient and cost-effective strategy provides a sustainable route to fluorinated molecules relevant to pharmaceuticals and agrochemicals.

## Graphical abstract

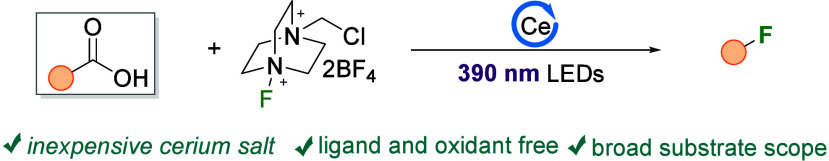

Visible-light-driven photoredox catalysis has emerged as a powerful tool for enabling organic transformations under mild conditions.^1^ Metallophotoredox catalysis is typically based on ruthenium and iridium complexes, benefiting from the long-lived excited state via metal-to-ligand charge transfer (MLCT).^2,3^ However, the high cost of these precious metals has driven a shift toward earth-abundant alternatives.^4–6^ Among these, cerium salts have gained recent attention due to their ability to undergo ligand-to-metal charge transfer (LMCT), where light excitation facilitates electron transfer to empty 4f orbitals.^7,8^ This enables selective cerium-ligand bond homolysis, generating radicals that serve as key intermediates in diverse synthetic applications. The photocatalytic properties of cerium compounds were first demonstrated by Sheldon and Kochi in 1968, who showed that cerium­(IV) pivalate undergoes photochemical activation to form pivalate radicals, which react with oxygen to yield *tert*-butyl alcohol, *tert*-butyl hydroperoxide, and acetone via decarboxylative oxygenation.^9,10^ Building on this foundation, Königs and co-workers reported a pioneering example of decarboxylative hydrazination of carboxylic acids.^11^ Recently, Yu and colleagues demonstrated decarboxylative amidation of carboxylic acids,^12^ while Roy and co-workers reported the alkylation of heterocycles using carboxylic acids as alkylating reagents.^13,14^ More recently, the Jin group developed decarboxylative iodination and bromination using NBS and NIS, respectively (Scheme 1).^15^ Beyond carboxylic acids, the group of Zhiwei has pioneered the use of cerium photocatalysis for the functionalization of alkanes^16–18^ and alcohols^19–23^ via the formation of alkoxy cerium intermediates.

**Scheme 1:**
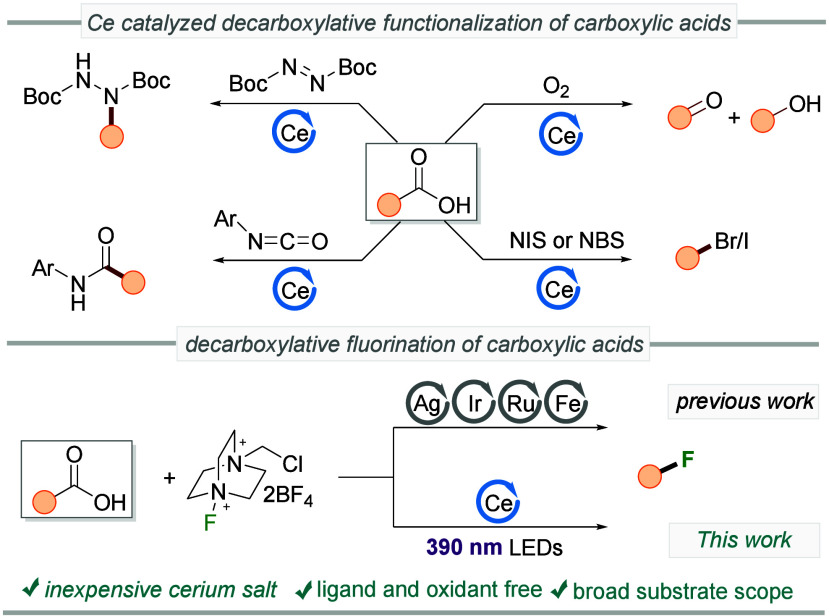
Decarboxylative Functionalization of Carboxylic Acids

Fluorine plays a crucial role in biologically active compounds, significantly enhancing their pharmacological properties. Due to its high electronegativity and small atomic radius, fluorine improves metabolic stability, increases lipophilicity, and strengthens molecular interactions with target biomolecules. Fluorination can enhance drug potency, selectivity, and bioavailability, making it a valuable strategy in pharmaceutical development.^24–30^ In recent years, several site-selective radical fluorination methods have been developed for the synthesis of alkyl fluorides from aliphatic carboxylic acids using Selectfluor as a fluorinating reagent. A notable early example was reported by the Li group, who demonstrated an Ag-catalyzed decarboxylative fluorination of alkyl carboxylic acids.^31^ This was followed by Sammis and co-workers, who achieved direct C–F bond formation in aryloxyacetic acids using Ru catalysis under high-intensity lamp irradiation.^32^ Subsequently, the MacMillan and Ye groups introduced distinct outer-sphere photocatalytic decarboxylative fluorination strategies employing photoredox catalysis.^33,34^ More recently, Hu and co-workers reported an alternative iron/bipyridyl ligand catalytic system.^35^ In light of the increasing demand for sustainable fluorination methodologies, we investigated the potential of simple cerium salts—derived from an earth-abundant and cost-effective transition metal—in combination with Selectfluor for the radical decarboxylative fluorination of carboxylic acids. This approach offers a relatively practical alternative to previously reported noble-metal-based photocatalytic systems and eliminates the need for organic ligands.

To investigate the Ce-photocatalyzed system, we began optimizing the decarboxylative fluorination using 2-(1,3-dioxoisoindolin-2-yl)­acetic acid (**1a**) as a model substrate. After a comprehensive evaluation of catalysts, bases, and solvents, the optimal reaction conditions were established as follows: CeCl_3_ (10 mol %) as the photocatalyst, Selectfluor (2–6 equiv) as the fluorine source, 2,6-lutidine (2 equiv) as the base, and a 1:1 mixture of acetonitrile and water as the solvent. The reaction was performed at room temperature under a nitrogen atmosphere with 390 nm light irradiation for 3 h, yielding the target product **2a** in 90% NMR yield (Table 1, entry 1). Notably, this catalytic system operates efficiently without the need for additional ligands. Among various cerium salts tested, only CeCl_3_ provided high conversion, while no product formation was observed in the absence of a catalyst (Table 1, entry 2). The use of cerium bromide as a catalyst led to a low yield, while the use of (*n*Bu_4_)_2_CeCl_6_ did not afford better results (Table 1, entries 3 and 4). Performing the reaction in the dark resulted in no conversion, confirming the necessity of light (Table 1, entry 5). Longer-wavelength irradiation led to only trace amounts of the product (Table 1, entry 6). We also demonstrated the necessity of conducting the reaction under an inert atmosphere, as no product formation was observed under aerobic conditions (Table 1, entry 7). Next, the role of the base was examined. Omitting the base entirely resulted in no product formation (Table 1, entry 8). Then, various organic and inorganic bases were tested, but none outperformed 2,6-lutidine in terms of yield and efficiency (Table 1, entries 9 and 10). Furthermore, the effect of solvents was explored. Replacing the CH_3_CN:H_2_O (1:1) mixture with DCM:H_2_O or acetone:H_2_O led to lower yields (Table 1, entries 11 and 12). Moreover, the addition of 1.5 equiv of the radical scavenger such as TEMPO and BHT to the standard conditions significantly inhibited the reaction (Table 1, entries 13 and 14).

**Table 1:**
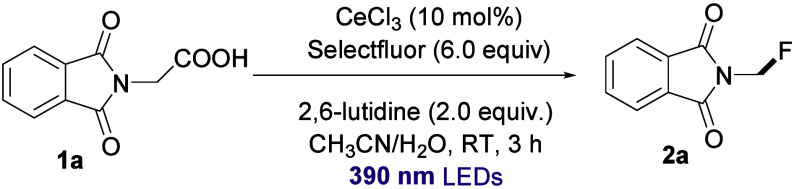
Screening of Reaction Conditions^a^

entry	*deviation from the standard* *conditions*	Yield^b^ (%)
1	none	90
2	no CeCl_3_	0
3	CeBr_3_	6
4	(*n*Bu_4_)_2_CeCl_6_	80
5	dark	0
6	450 nm LEDs	0
7	under air	0
8	no base	0
9	pyridine as base	53
10	Na_2_CO_3_ as base	35
11	DCM: H_2_O as solvent	20
12	acetone: H_2_O as solvent	71
13	1.5 equiv of TEMPO	18
14	1.5 equiv of BHT	0

a Standard condition: substrate **1a** (0.1 mmol), CeCl_3_ (0.01 mmol, 2.4 mg), Selectfluor (0.6 mmol, 212.5 mg), 2,6-lutidine (0.2 mmol, 23 μL), CH_3_CN/H_2_O (1:1, 1 mL), rt, N_2_ atmosphere, 390 nm LEDs, 3 h.

b Determined by ^19^F NMR using *p*-fluorotoluene as an internal standard. TEMPO is 2,2,6,6-tetramethylpiperidine 1-oxide, BHT is Butylated hydroxytoluene.

Next, after optimizing the conditions for decarboxylative fluorination, we explored the substrate scope using a wide variety of carboxylic acids. As shown in Table 2, primary, secondary, and tertiary carboxylic acids reacted efficiently with Selectfluor under standard conditions, leading to the synthesis of diverse alkyl fluorides (**2a–2u**). In most cases, we report both isolated and ^19^F NMR yields. The lower isolated yields in some cases can be attributed to volatility-related losses. To our delight, the catalytic system tolerated a broad range of functional groups, including esters, amides, imides, carbamates, ketones, ethers, and aryl halides. Notably, the method performed well with challenging primary carboxylic acids, affording the corresponding primary alkyl fluorides (**2a–2f**) in moderate to high yields. In the case of benzylic carboxylic acid **1b**, we observed the formation of difluorinated product **2b** in 66% isolated yield, likely due to a subsequent C–H fluorination of the monofluorinated intermediate via hydrogen atom transfer with chloride radicals. The catalytic system was also compatible with tertiary carboxylic acids, yielding tertiary alkyl fluorides (**2g** and **2h**) in moderate yields using 2 equiv of the fluorinating reagent. For secondary carboxylic acids, the reaction also proceeded efficiently using only 2 equiv of Selectfluor. Various cyclic amino acid derivatives proved to be suitable substrates, affording the corresponding fluorinated products (**2i–2m**) in good to moderate yields. Similarly, carboxylic acid **1n** furnished the desired product in good yield with moderate diastereoselectivity. Secondary benzylic carboxylic acid **1p** was converted to the desired product in excellent yield, and comparable results were obtained with acyclic carboxylic acids bearing phthalimide moieties (**2q**, **2r**).

**Table 2:**
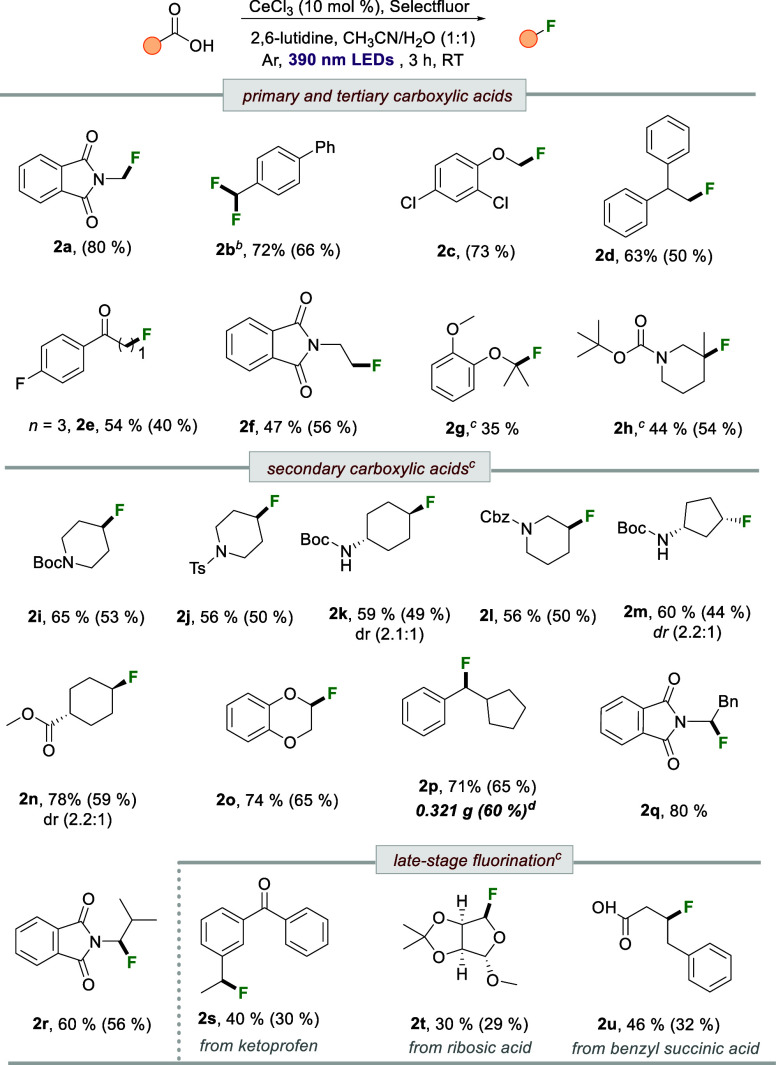
Scope of Carboxylic Acids^a^

a Reaction conditions: **1** (0.4 mmol), CeCl**_3_** (0.04 mmol, 9.8 mg), Selectfluor (6 equiv), 2,6-lutidine (0.8 mmol, 92 μL), CH_3_CN/H_2_O (1:1, 4 mL), RT, LEDs (40 W, 390 nm), 3 h, ^19^F NMR using *p*-fluorotoluene as an internal standard, isolated yields are in parentheses.

b Without base and 2 equiv of Selectfluor.

c Selectfluor (2 equiv).

d 3 mmol scale in batch, 12 h.

Afterward, we evaluated the photoexcited cerium system for the late-stage modification of complex carboxylic acid derivatives (**1s–1u**). Notably, a drug molecule such as Ketoprofen was well-tolerated under the catalytic conditions, yielding the corresponding fluorinated products (**2s**). Additionally, ribosic acid was converted to its fluorinated product **2t**, albeit in a low yield. Finally, we tested 2-benzylsuccinic acid **1u**, which contains both primary and secondary carboxylic acid groups. To our delight, the reaction selectively fluorinated the secondary carboxylic acid, while leaving the primary carboxylic group untouched.

To gain insight into the reaction pathways, we conducted preliminary mechanistic studies (see Supporting Information for details). Light on–off experiments confirmed that the cerium-catalyzed decarboxylative fluorination requires continuous irradiation to proceed (Figure 1a). Additionally, we monitored the reaction progress in real time using in situ ATR-IR spectroscopy with substrate **1a**. As shown in Figure 1b, the reaction mixture contains three key components: carboxylic acid (1774 cm^–1^), carbon dioxide (2342 cm^–1^), and alkyl fluoride product (1367 cm^–1^). The ATR-IR analysis (Figure 1c) reveals distinct spectral changes during the reaction. Initially, the peak at 1774 cm^–1^, corresponding to the asymmetric stretching of the carboxyl group, increases due to substrate deprotonation, forming the carboxylate anion. This signal then diminishes as CO_2_ evolves and saturates over time. Concurrently, the peak at 1367 cm^–1^, attributed to C–F bond formation, progressively intensifies and stabilizes after approximately 3 h, indicating reaction completion.

**Figure 1:**
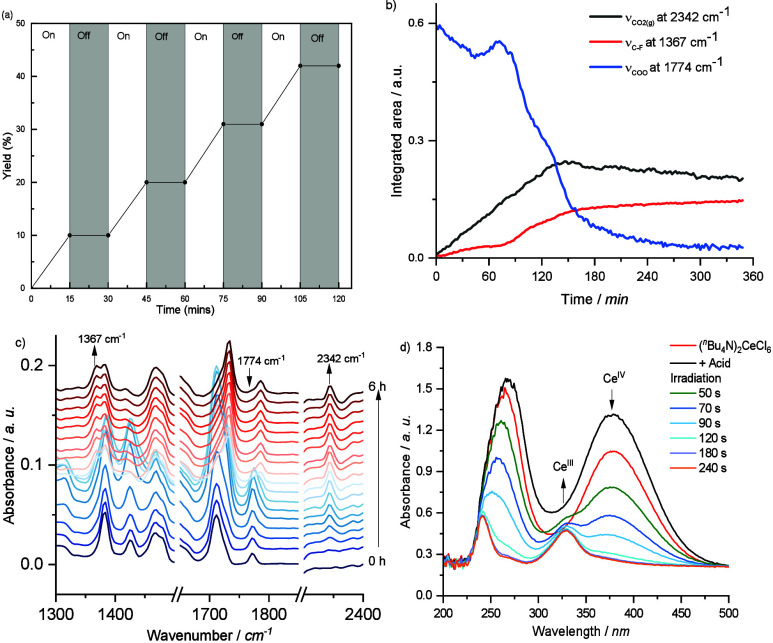
(a) Light on/off experiment; (b) ATR-IR of the reaction progress for substrate, product, and CO_2_; (c) *In situ* ATR-IR spectra of the reaction mixture for CO_2_ detection; (d) UV–vis spectra of the solution of Ce­(IV) catalyst and **1a** under the irradiation of light for the given interval of time.

To further elucidate the role of cerium in the reaction, we conducted a UV–vis study using the synthesized Ce­(IV) catalyst, (*n*Bu_4_)_2_CeCl_6_. The UV–vis spectrum of the Ce­(IV) complex exhibits a sharp absorption band at 378 nm (Figure 1d). In the absence of light, the addition of carboxylic acid **1a** does not alter the spectrum, and the characteristic Ce­(IV) absorption at 378 nm remains unchanged. However, upon irradiation, a rapid reduction of Ce­(IV) to Ce­(III) was observed, evidenced by the emergence of a new absorption band at 330 nm. This result supports the formation of a cerium­(IV) carboxylate complex, which, upon light irradiation, undergoes LMCT. This process generates Ce­(III) species and a carboxylate radical, initiating decarboxylative fluorination process.

Based on our experimental mechanistic studies and previous reports, the proposed reaction mechanism is outlined in Scheme 2.^4,36^ The catalytic cycle is initiated by the oxidation of the Ce­(III) precatalyst to Ce­(IV) by TEDA^2+^ **^.^** (*N*-(chloromethyl)­triethylenediamine), which is generated after the fluorine transfer step. This oxidation is thermodynamically feasible, given the redox potential of Ce­(III)/Ce­(IV) (E_1/2_ = 0.41 V vs SCE in MeCN). Subsequently, the alkyl carboxylic acid undergoes deprotonation by an organic base and coordinates with the oxidized Ce­(IV), forming a cerium­(IV) carboxylate complex. Upon irradiation, this complex undergoes homolysis via LMCT, generating a carboxyl radical. The unstable carboxyl radical rapidly decomposes, releasing CO_2_ and forming an alkyl radical. The resulting alkyl radical then reacts with Selectfluor, leading to the formation of an alkyl fluoride and the oxidation of Selectfluor to TEDA^2+**.**^. The catalytic cycle is closed when TEDA^2+**.**^, a highly oxidizing species (E_1_/_2_ (TEDA^2+**.**^/TEDA^+^) = +0.79 V), reoxidizes Ce­(III) back to Ce­(IV), regenerating the active catalyst and the reduced Selectfluor species (TEDA^+^). Notably, TEDA^2+**.**^ is a more efficient oxidant than Selectfluor itself (E_1_/_2_ (Selectfluor/TEDA^2+**.**^) = +0.33 V), reinforcing its crucial role in closing the catalytic cycle.^37^

**Scheme 2:**
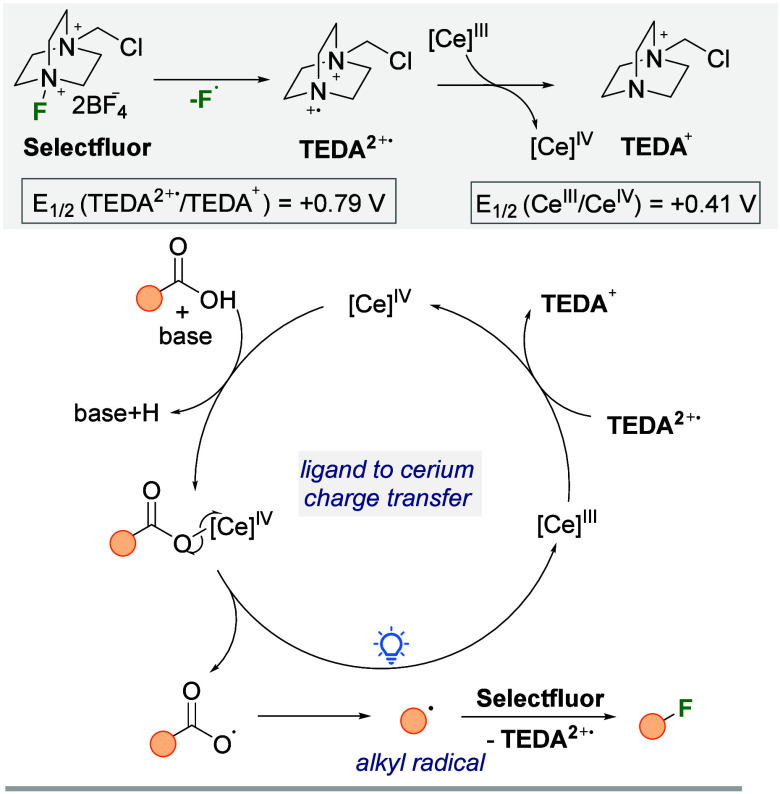
Proposed Mechanism for Decarboxylative Fluorination

In summary, we have developed a novel method for the decarboxylative fluorination of carboxylic acids using an inexpensive cerium salt under ligand-free conditions. This strategy demonstrates a broad substrate scope, high functional group tolerance, and applicability in the late-stage functionalization of complex molecules. Mechanistic investigations suggest that the reaction proceeds via the formation of alkyl radicals through light-induced LMCT of the cerium­(IV) carboxylate. This approach offers a practical and efficient pathway for fluorination, expanding the toolbox for radical-based synthetic methodologies.

## Supplementary Material



## Data Availability

The data underlying this study are available in the published article and its Supporting Information.

## Notes

Experimental procedures, analytical data for all new compounds, and NMR spectra (PDF)
