# Supplementary material for: Ligand-Free Cerium-Catalyzed Decarboxylative Fluorination of Carboxylic Acids
Source: ACS Org Inorg Au. 2025 Apr 23;5(3):166–70. doi: 10.1021/acsorginorgau.5c00024 (PMC12142436; doi:10.1021/acsorginorgau.5c00024)
Supplement: Supplementary file 1 [file gg5c00024_si_001.pdf]

Supporting Information

Ligand-Free Cerium Catalyzed Decarboxylative Fluorination of Carboxylic Acids.

Maham Azhar, Tianyou Peng, and Osama El-Sepelgy*

[†]Leibniz Institute for Catalysis e.V., Albert-Einstein-Str. 29a, 18059 Rostock, Germany

Email: Osama.Elsepelgy@Catalysis.de.

Table of Contents

1. General information S3

2. Optimization of reaction conditions and control experiments S4

3. General procedures S8

4. Characterization data S9

5. Mechanistic studies S15

5.1. ON/OFF experiment S15

5.2. *In-situ* Infrared Spectroscopy S15

5.3. UV-vis Spectroscopy S17

6. NMR spectra S19

7. References S59

1. General information

Unless otherwise noted, all Starting materials were purchased from commercial suppliers and used without further purification. The reactions were monitored by thin layer chromatography (TLC) with aluminum sheets silica gel 60 F_{254} from Merck, and flash column chromatography purifications were performed using silica gel 60 (63-200 μm) from MACHEREY-NAGEL. ^{1}H and ^{13}C NMR spectra were recorded with Bruker AV 300 (300 MHz), AV 400 (400 MHz) or Fourier 300 (300 MHz) NMR spectrometers. Chemical shifts (δ) are given relative to solvent: references for $CDCl_3$ were 7.26 ppm (^{1}H NMR) and 77.16 ppm (^{13}C NMR). And all signals were reported in parts per million (ppm) and spin-spin coupling constants (J) are given in Hz, while multiplicities are abbreviated by s (singlet), d (doublet), t (triplet), q (quartet), br (broad), m (multiplet). All IR data were collected by attenuated total reflectance (ATR, Bruker Alpha FT-IR spectrometer) and wavenumbers ν are given in cm^{-1}. All measurements were carried out at room temperature unless otherwise stated. For light-promoted reactions: Use Kessil lamp (40 W, 390 nm, manufacturer: Kessil, model: PR160L-390 nm). The distance between the light source and the irradiation vessel is about 2 cm, and the reaction vessel is cooled with a fan.

2. Optimization of reaction conditions and control experiments

Table S1: Optimization of Catalyst[a]

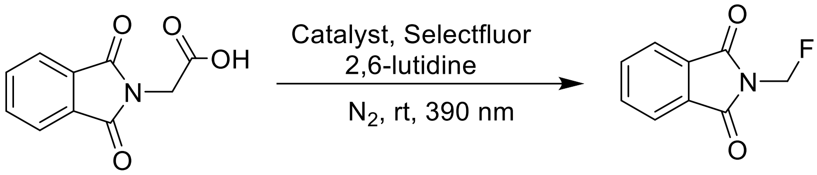

Entry	Catalyst	Wavelength	Yield (%)[b]
1	$CeBr_3$	390 nm	6
2	$Ce(OTf)_2$	390 nm	70
**3**	**$CeCl_3$**	**390 nm**	**86**

[a]Reaction conditions: N-Phthaloylglycine (0.1 mmol, 20.5 mg), Catalyst (10 mol %), Selectfluor (6 equiv. 212.55 mg), 2,6-lutidine (2 equiv., 0.2 mmol, 23 µL), 390 nm, degassed solvent (MeCN: H_2O= 1:1, 1 mL), room temperature, 6 h, [b]Determined by ^{19}F NMR using *p*-fluorotoluene as an internal standard.

Table S2: Optimization for catalyst loading[a]

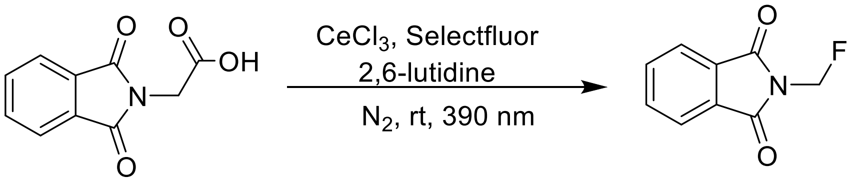

Entry	Catalyst loading	Yield (%)[b]
1	5 mol % $CeCl_3$	75
**2**	**10 mol % $CeCl_3$**	**86**
3	15 mol % $CeCl_3$	37
4	20 mol % $CeCl_3$	45
5	10 mol % $CeCl_3$+ 20 mol % TBACl	55

[a]Reaction conditions: *N*-Phthaloylglycine (0.1 mmol, 20.5 mg), $CeCl_3$ (x mol %), Selectfluor (6 equiv. 212.55 mg), 2,6-lutidine (2 equiv., 0.2 mmol, 23 µL), 390 nm, degassed solvent (MeCN: H_2O= 1:1, 1 mL), room temperature, 6 h, [b]Determined by ^{19}F NMR using *p*-fluorotoluene as an internal standard.

Table S3: Optimization of Base[a]

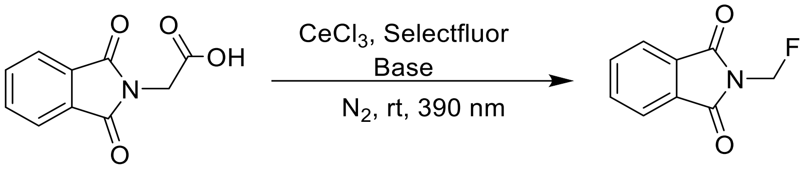

Entry	Base	Yield (%)[b]
1	NaOH	21
2	KOH	20
3	Na_2CO_3	35
4	Pyridine	53
5	2,4,6-Collidine	60
**6**	**2,6-Lutidine**	**86**
7	-	traces

[a]Reaction conditions: *N*-Phthaloylglycine (0.1 mmol, 20.5 mg), $CeCl_3$ (10 mol %), Selectfluor (6 equiv. 212.55 mg), Base (2 equiv.), 390 nm, degassed solvent (MeCN: H_2O= 1:1, 1 mL), room temperature, 6 h, [b]Determined by ^{19}F NMR using *p*-fluorotoluene as an internal standard.

Table S4: solvent screening

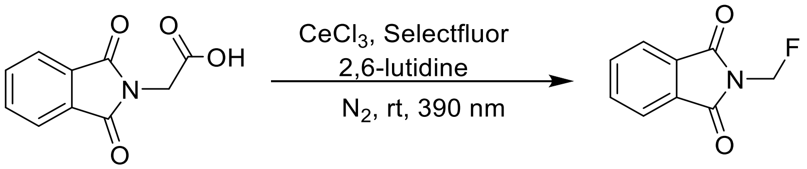

Entry	Solvent	Yield (%)[b]
1	DCM	Traces
2	Acetone	10
3	H_2O	24
4	MeCN	8
5	DCM: H_2O (1:1)	20
6	Acetone: H_2O (1:1)	71
**7**	**MeCN: H_2O (1:1)**	**86**

[a]Reaction conditions: *N*-Phthaloylglycine (0.1 mmol, 20.5 mg), $CeCl_3$ (10 mol %), Selectfluor (6 equiv. 212.55 mg), 2,6-lutidine (2 equiv., 0.2 mmol, 23 μL), 390 nm, degassed solvent (1 mL), room temperature, 6 h, [b]Determined by ^{19}F NMR using *p*-fluorotoluene as an internal standard.

Table S5: Optimization for light source[a]

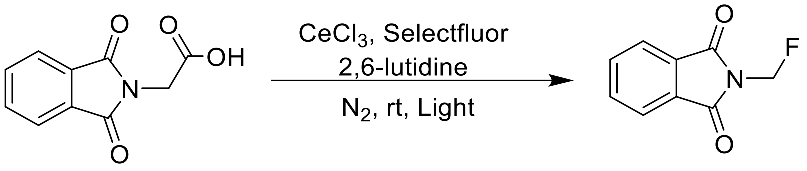

Entry	Light	Yield (%)[b]
1	Blue (450 nm)	Traces
2	427 nm	25
**3**	**390 nm**	**86**
4	Dark	n.d

[a]Reaction conditions: *N*-Phthaloylglycine (0.1 mmol, 20.5 mg), $CeCl_3$ (10 mol %), Selectfluor (6 equiv. 212.5 mg), 2,6-lutidine (2 equiv., 23 μL), Light, degassed solvent (MeCN: H_2O= 1:1, 1 mL), room temperature, 6 h, [b]Determined by ^{19}F NMR using *p*-fluorotoluene as an internal standard.

Table S6: Selectfluor optimization for primary carboxylic acids[a]

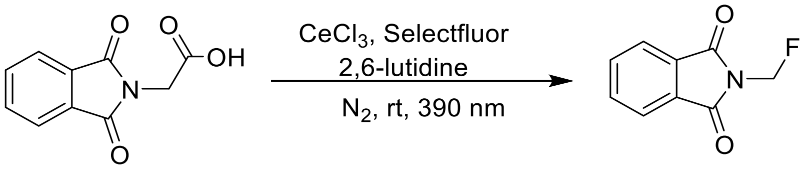

Entry	Selectfluor	Yield (%)[b]
1	2	10
2	3	30
3	4	52
4	5	64
**5**	**6**	**86**

[a]Reaction conditions: *N*-Phthaloylglycine (0.1 mmol, 20.5 mg), $CeCl_3$ (10 mol %), Selectfluor (x equiv.), 2,6-lutidine (2 equiv., 23 μL), 390 nm, Degassed solvent (MeCN: H_2O= 1:1, 1 mL), room temperature, 6 h, [b]Determined by ^{19}F NMR using *p*-fluorotoluene as an internal standard.

Table S7: Optimization for Selectfluor and base for secondary and tertiary carboxylic acid[a]

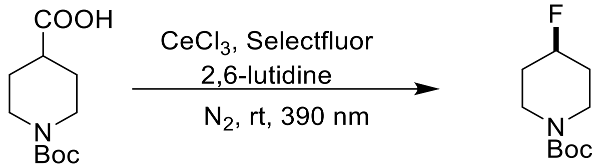

Entry	Selectfluor	2,6-Lutiidne	Light	Yield (%)[b]
1	2	1.2	390 nm	47
2	2	1.5	390 nm	54
3	2	1.8	390 nm	63
**4**	**2**	**2**	**390 nm**	**68**
5	2	2.5	390 nm	33
6	2	3.2	390 nm	46
7	1.2	2	390 nm	24
8	1.5	2	390 nm	48
9	1.8	2	390 nm	48
10	2.5	2.5	427 nm	41

[a]Reaction conditions: 1-(tert-butoxycarbonyl)piperidine-4-carboxylic acid (0.1 mmol, 23 mg), $CeCl_3$ (10 mol %), Selectfluor (x equiv.), 2,6-lutidine (x equiv.), 390 nm, degassed solvent (MeCN: H_2O= 1:1, 1 mL), room temperature, 6 h, [b]Determined by ^{19}F NMR using *p*-fluorotoluene as an internal standard.

3. General procedures

3.1 General procedure for catalysis of fluorinated products

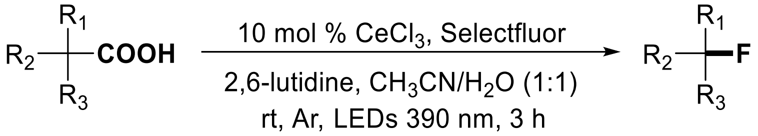

In an oven-dried 25 mL Schlenk tube, carboxylic acid (0.4 mmol), $CeCl_3$ (9.9 mg, 10 mol%), and Selectfluor (2.0 equivalents for secondary and tertiary carboxylic acids; 6.0 equivalents for primary carboxylic acids) were added. Degassed acetonitrile/water (4.0 mL, 1:1 v/v) and 2,6-lutidine (92.7 μL, 2.0 equiv.) were then added to the reaction mixture. The solution was subjected to three freeze–pump–thaw cycles to remove dissolved oxygen. The reaction mixture was stirred and irradiated under an argon atmosphere using a 390 nm Kessil lamp (40 W) for 3.0 hours, with cooling provided by a fan. After completion, the mixture was quenched with water and extracted with dichloromethane (3 × 20 mL). The combined organic layers were dried over anhydrous Na_2SO_4, filtered, and concentrated under reduced pressure. The crude product was purified by column chromatography on silica gel to afford the desired fluorinated compound.

4. Characterization data

2-(fluoromethyl)isoindoline-1,3-dione (2a) [1]

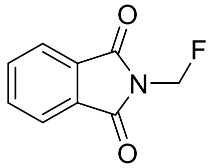

2a was purified with column chromatography on silica gel (DCM) to yield 80 %, 57 mg, white solid.

^{1}H NMR (400 MHz, CDCl$_3$) δ 7.98 – 7.92 (m, 2H), 7.84 – 7.76 (m, 2H), 5.83 (s, 1H), 5.70 (s, 1H).
^{19}F NMR (282 MHz, CDCl$_3$) δ -175.68 (t, *J* = 52.5 Hz, 1F).
^{13}C{^{1}H} NMR (101 MHz, CDCl$_3$) δ 166.5 (d, *J* = 2.2 Hz), 134.9, 131.6, 124.2, 74.9 (d, *J* = 198.4 Hz).

4-(difluoromethyl)-1,1'-biphenyl (2b) [2]

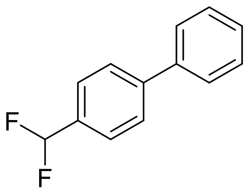

2b was purified with column chromatography on silica gel (Pentane) to yield 66 %, 54 mg, white solid (NMR yield 72%).

^{1}H NMR (400 MHz, CDCl$_3$) δ 7.71 – 7.65 (m, 2H), 7.63 – 7.57 (m, 4H), 7.51 – 7.44 (m, 2H), 7.43 – 7.36 (m, 1H), 6.71 (t, *J* = 56.5 Hz, 1H).
^{19}F NMR (376 MHz, CDCl$_3$) δ -110.34 (d, *J* = 56.5, 2F).
^{13}C{^{1}H} NMR (101 MHz, CDCl$_3$) δ 143.7 (t, *J* = 2.0 Hz), 140.2, 133.2 (t, *J* = 22.4 Hz), 128.9, 127.9, 127.5, 127.3, 126.1 (t, *J* = 6.1 Hz), 114.8 (t, *J* = 237.6 Hz).

2,4-dichloro-1-(fluoromethoxy)benzene (2c) [1]

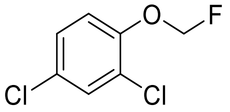

2c was purified with column chromatography on silica gel (DCM) to yield 73 %, 57 mg white solid.

^{1}H NMR (300 MHz, CDCl$_3$) δ 7.42 (d, *J* = 2.14 Hz, 1H), 7.23 (dd, *J* = 8.7, 2.6 Hz, 1H), 7.14 (dd, *J* = 8.9, 0.9 Hz, 1H), 5.80 (s, 1H), 5.62 (s, 1H).
^{19}F NMR (282 MHz, CDCl$_3$) δ -149.60 (t, *J* = 54.2 Hz, 1F).
^{13}C{^{1}H} NMR (75 MHz, CDCl$_3$) δ 151.3 (d, *J* = 3.1 Hz), 130.3, 129.6, 128.0, 125.1, 118.4 (d, *J* = 1.7 Hz), 102.6, 99.6.

(2-fluoroethane-1,1-diyl) dibenzene (2d) [1]

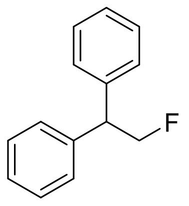

2d was purified with column chromatography on silica gel (Penatne) to yield 50 %, 40 mg, colorless oil (NMR yield 63%).

^{1}H NMR (300 MHz, CDCl3) δ 7.39 – 7.29 (m, 4H), 7.28 – 7.21 (m, 6H), 5.00 (d, J = 7.0 Hz, 1H), 4.85 (d, J = 7.0 Hz, 1H), 4.47 – 4.34 (m, 1H).
^{19}F NMR (282 MHz, CDCl3) δ -214.74 (s, 1F).
^{13}C{^{1}H} NMR (101 MHz, CDCl3) δ 140.3 (d, J = 4.9 Hz), 128.6, 128.3, 126.9, 85.3 (d, J = 175.0 Hz), 51.3 (d, J = 19.7 Hz).

4-fluoro-1-(4-fluorophenyl)butan-1-one (2e) [1]

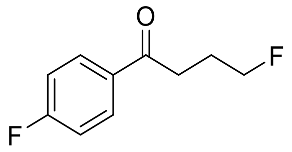

2e was purified with column chromatography on silica gel (Pentane: Ethyl acetate = 20:1) to yield 40 %, 40 mg, colorless oil (NMR yields 54 %).

^{1}H NMR (300 MHz, CDCl3) δ 8.06 - 7.94 (m, 2H), 7.20 - 7.06 (m, 2H), 4.63 (t, J = 5.7 Hz, 1H), 4.48 (t, J = 5.7 Hz, 1H), 3.12 (t, J = 7.1 Hz, 2H), 2.26 - 1.99 (m, 2H).
^{19}F NMR (282 MHz, CDCl3) δ -105.14 (s, 1F), -220.25 (s, 1F).
^{13}C{^{1}H} NMR (75 MHz, CDCl3) δ 198.0, 168.0, 164.7, 133.8, 131.2 (d, J = 9.4 Hz), 116.3 (d, J = 21.9 Hz), 84.9, 82.7, 34.5 (d, J = 4.0 Hz), 25.4 (d, J = 20.2 Hz).

2-(2-fluoroethyl)isoindoline-1,3-dione (2f) [3]

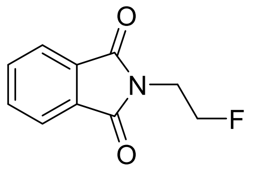

2f was purified with column chromatography on silica gel (DCM) to yield 47 %, 36 mg, white solid (NMR yields: 56 %).

^{1}H NMR (400 MHz, CDCl3) δ 7.91 – 7.84 (m, 2H), 7.76 – 7.72 (m, 2H), 4.65 (td, J = 46.9, 5.2 Hz, 2H), 4.03 (td, J = 24.0, 5.1 Hz, 2H).
^{19}F NMR (376 MHz, CDCl3) δ -224.69 (tt, J = 47.21, 24.32 Hz, 1F).
^{13}C{^{1}H} NMR (101 MHz, CDCl3) δ 168.0, 134.1, 131.9, 123.4, 80.4 (d, J = 171.9 Hz), 38.2 (d, J = 22.0 Hz).

tert-butyl 3-fluoro-3-methylpiperidine-1-carboxylate (2h) [1]

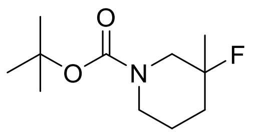

2h was purified with column chromatography on silica gel (DCM) to yield 44 %, 38 mg, colorless oil (NMR yields 54 %).

^{1}H NMR (300 MHz, CDCl3) δ 3.85 - 3.61 (m, 2H), 3.01 - 2.84 (m, 2H), 2.02 - 1.76 (m, 2H), 1.65 - 1.53 (m, 2H), 1.45 (s, 9H), 1.33 (d, J = 21.0 Hz, 3H).

^{19}**F NMR (282 MHz, CDCl3)** δ -152.42 (d, *J* = 226.6 Hz, 1F).
^{13}**C{^{1}H} NMR (75 MHz, CDCl3)** δ 155.1, 91.2 (d, J = 172.4 Hz), 79.7, 52.7, 42.9, 35.3 (d, J = 22.6 Hz), 28.4, 24.3 (d, J = 23.7 Hz), 21.5.

tert-butyl 4-fluoropiperidine-1-carboxylate (2i) [1]

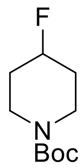

2i was purified with column chromatography on silica gel (DCM) to yield 53 %, 43 mg, colorless oil (NMR yield 65 %).

^{1}**H NMR (300 MHz, CDCl3)** δ 4.90 – 4.71 (m, 1H), 3.54 – 3.42 (m, 4H), 1.88 – 1.74 (m, 4H), 1.46 (s, 9H).
^{19}**F NMR (282 MHz, CDCl3)** δ -182.23 (s, 1F).
^{13}**C{^{1}H} NMR (75 MHz, CDCl3)** δ 154.8, 88.2 (d, *J* = 171.0 Hz), 79.7, 39.8, 31.1 (d, *J* = 19.8 Hz), 28.4.

4-fluoro-1-tosylpiperidine (2J) [1]

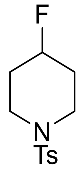

2J was purified with column chromatography on silica gel (DCM) to yield 50 %, 51 mg, white solid (NMR yield 56 %).

^{1}**H NMR (300 MHz, CDCl3)** δ 7.58 – 7.55 (m, 2H), 7.26 – 7.23 (m, 2H), 4.76 – 4.57 (m, 1H), 3.28 – 3.23 (m, 2H), 2.83 – 2.76 (m, 2H), 2.35 (s, 3H), 1.91 – 1.78 (m, 4H).
^{19}**F NMR (282 MHz, CDCl3)** δ -185.49 (s, 1F).
^{13}**C{^{1}H} NMR (75 MHz, CDCl3)** δ 143.7, 133.1, 129.7, 127.7, 86.3 (d, *J* = 171.0 Hz), 41.8 (d, *J* = 4.4 Hz), 30.5 (d, *J* = 20.2 Hz), 21.6.

tert-butyl ((1r,4r)-4-fluorocyclohexyl)carbamate (2k) [1]

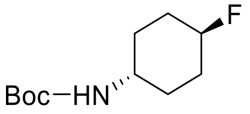

According to the general procedure for catalysis of fluorinated products, **2k** was purified with column chromatography on silica gel (DCM) to yield 49 %, 46 mg, white solid (NMR yield 59 %). The dr value (2.08:1) was detected by ^{19}F NMR.
^{1}**H NMR (300 MHz, CDCl3)** δ 4.78 - 4.59 (m, 1H), 4.53 - 4.34 (m, 1H), 3.42 (s, 1H), 1.97 - 1.91 (m, 3H), 1.76 - 1.70 (m, 2H), 1.54 - 1.43 (m, 4H), 1.38 (s, 9H).
^{19}**F NMR (282 MHz, CDCl3)** δ -174.47 (minor), -184.01(major).
^{13}**C{^{1}H} NMR (75 MHz, CDCl3)** δ 155.2, 92.0, 89.7, 89.0, 86.8, 79.3, 48.2, 30.5 (d, J = 20.0 Hz), 29.8 (d, J = 7.0 Hz), 29.4, 28.4 (d, J = 1.5 Hz), 27.5 (d, J = 2.4 Hz).

benzyl (R)-3-fluoropiperidine-1-carboxylate (2l) [1]

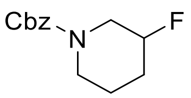

2l was purified with column chromatography on silica gel (DCM) to yield 50 %, 47 mg, yellow oil (NMR yield 56 %).

^{1}H NMR (400 MHz, CDCl$_3$) δ 7.29 – 7.19 (m, 5H), 5.05 (s, 2H), 4.53 (d, *J* = 46.6 Hz, 1H), 3.67 (s, 1H), 3.48-3.34 (m, 2H), 3.21 (s, 1H), 1.82 – 1.70 (m, 3H), 1.42 (s, 1H).
^{19}F NMR (376 MHz, CDCl$_3$) δ -184.67 (s,1F).
^{13}C{^{1}H} (101 MHz, CDCl$_3$) δ 155.5, 136.7, 128.5, 128.0, 127.9, 86.2 (d, *J* = 175.0 Hz), 67.2, 47.9 (d, *J* = 22.4 Hz), 43.8, 29.7 (d, *J* = 20.2 Hz), 20.9 (d, *J* = 27.6 Hz).

***Tert*-butyl *N*-[(1S)-3-fluorocyclopentyl]carbamate (2m)** [1]

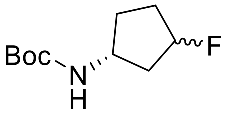

2m was purified with column chromatography on silica gel (Pentane: Ethyl acetate = 20:1) to yield 44 %, 36 mg, white solid (NMR yield 60 %). The dr value (2.2:1) was detected by ^{19}F NMR.
^{1}H NMR (300 MHz, CDCl$_3$) δ 5.13 (dt, *J* = 53.6, 5.0 Hz, 1H), 4.84 (s, 0.32H), 4.54 (s, 0.62H), 4.21 - 4.13 (m, 1H), 2.40 - 2.00 (m, 3H), 1.88 - 1.57 (m, 3H), 1.42 (s, 9H).
^{19}F NMR (376 MHz, CDCl$_3$) δ -167.70 (minor), -169.36 (major).
^{13}C{^{1}H} NMR (75 MHz, CDCl$_3$) δ 155.4, 96.1, 93.8, 79.2, 50.5, 40.8 (d, *J* = 21.1 Hz), 32.4, 32.0 (d, *J* = 22.1 Hz), 31.6 (d, *J* = 4.8 Hz), 30.9, 29.7, 28.4.

Methyl 4-fluorocyclohexane-1-carboxylate (2n) [1]

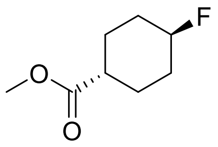

2n was purified with column chromatography on silica gel (Pentane: Ethyl acetate= 20:1) to yield 59 %, 38 mg, colorless oil (NMR yield 78 %). The dr value (2.2:1) was detected by ^{19}F NMR.

^{1}H NMR (300 MHz, CDCl$_3$) δ 4.81 – 4.60 (m, 0.73H) (major), 4.58 – 4.34 (m, 0.25 H) (minor), 3.61 (s, 2.06 H) (major), 3.60 (s, 0.78H) (minor), 2.30 – 2.23 (m, 1H), 2.07 – 1.82 (m, 2.8H), 1.84 – 1.65 (m, 3H), 1.61– 1.37 (m, 2.8H).
^{19}F NMR (282 MHz, CDCl$_3$) δ -172.64 (minor), -183.15 (major).
^{13}C{^{1}H} NMR (101 MHz, CDCl$_3$) δ 175.6, 91.8 (minor), 88.2 (d, *J* = 168.7 Hz) (major),51.7 (d, *J* = 4.5 Hz), 41.6 (major), 41.3 (minor), 31.6 (major), 31.0 (minor), 29.9 (d, *J* = 21.1 Hz), 25.9 (d, *J* = 10.3 Hz), 23.2 (d, *J* = 2.9 Hz).

(S)-2-fluoro-2,3-dihydrobenzo[b][1,4]dioxine (2o) [4]

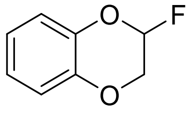

2o was purified with column chromatography on silica gel (hexanes to 2 % EtOAc/hexanes) 65 %, 53 mg, colorless oil (NMR yield 74 %).

^{1}H NMR (300 MHz, CDCl$_3$) δ 7.01 - 6.87 (m, 4H), 6.03 (ddd, *J* = 53.7, 1.4, 0.8 Hz, 1H), 4.39 (ddd, *J* = 12.0, 4.0, 1.4 Hz, 1H), 4.03 (ddd, *J* = 28.1, 12.0, 0.8 Hz, 1H).
^{19}F NMR (282 MHz, CDCl$_3$) δ -131.26 (ddd, *J* = 53.4, 28.2, 3.9 Hz, 1F).

$^{13}C\{^1H\}$ NMR (75 MHz, $CDCl_3$) δ 142.9, 123.1, 122.4, 117.5 (d, *J* = 14.8 Hz), 102.5, 99.6, 64.4 (d, *J* = 24.1 Hz).

(S)-(cyclopentylfluoromethyl)benzene(2p) [4]

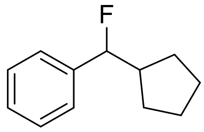

2p was purified with column chromatography on silica gel (hexanes) to yield 65 %, 53 mg, colorless oil (NMR yield 71 %).

^1H NMR (300 MHz, $CDCl_3$) δ 7.63 - 7.03 (m, 5H), 5.30 - 4.89 (m, 1H), 2.49 - 2.03 (m, 1H), 1.87 - 1.69 (m, 1H), 1.64 - 1.29 (m, 6H), 1.25 - 1.15 (m, 1H).
^{19}F NMR (282 MHz, $CDCl_3$) δ -172.34 (dd, *J* = 16.79, 16.02 Hz, 1F).
$^{13}C\{^1H\}$ NMR (75 MHz, $CDCl_3$) δ 140.2, 128.3, 128.2 (d, *J* = 2.2 Hz), 126.2 (d, *J* = 6.5 Hz), 99.34 97.1, 45.9 (d, *J* = 22.4 Hz), 28.9 - 28.5 (m), 25.4 (d, *J* = 6.8 Hz).

(R)-2-(1-fluoro-2-phenylethyl) isoindoline-1,3-dione (2q) [4]

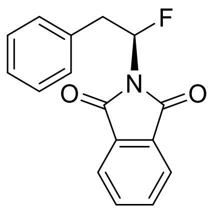

2q was purified with column chromatography on silica gel (Ethyl acetate: hexanes = 1: 2) to yield 80 %, 86 mg, white solid.

^1H NMR (400 MHz, $CDCl_3$) δ 7.86 (dd, *J* = 3.1, 5.5 Hz, 2H), 7.74 (dd, *J* = 3.7, 5.5 Hz, 2H), 7.34 - 7.15 (m, 5H), 6.36 (dt, *J* = 7.2, 47.6 Hz, 1H), 3.95 - 3.53 (m, 2H).
^{19}F NMR (376 MHz, $CDCl_3$) δ -144.73 (dd, *J* = 9.1, 19.7 Hz), -144.85 (dd, *J* = 9.2, 20.1 Hz).
$^{13}C\{^1H\}$ NMR (101 MHz, $CDCl_3$) δ 166.9, 134.8, 134.7 (d, *J* = 4.8 Hz), 131.4, 129.3, 128.8, 127.3, 124.0, 91.4, 89.3, 37.5 (d, *J* = 27.3 Hz).

(S)-2-(1-fluoro-2-methylpropyl)isoindoline-1,3-dione (2r) [3]

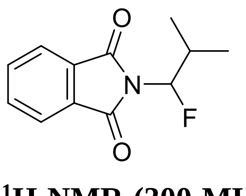

2r was purified with column chromatography on silica gel (DCM) to yield 56 %, 50 mg, white solid (NMR yield 60 %).

^1H NMR (300 MHz, $CDCl_3$) δ 7.97 - 7.90 (m, 2H), 7.84 - 7.77 (m, 2H), 5.73 (dd, *J* = 47.5, 10.3 Hz, 1H), 3.17 (ddp, *J* = 10.2, 7.7, 6.7 Hz, 1H), 1.22 (dd, *J* = 6.6, 0.9 Hz, 3H), 0.87 (dd, *J* = 6.8, 0.8 Hz, 3H).
^{19}F NMR (282 MHz, $CDCl_3$) δ -150.58 (dd, *J* = 47.5, 7.8 Hz, 1F).
$^{13}C\{^1H\}$ NMR (75 MHz, $CDCl_3$) δ 166.9, 134.7, 131.5, 123.9, 96.4, 93.7, 29.2 (d, *J* = 24.2 Hz), 18.9 (d, *J* = 2.0 Hz), 17.4 (d, *J* = 7.4 Hz).

(4-(1-fluoroethyl)phenyl)(phenyl)methanone (2s) [1]

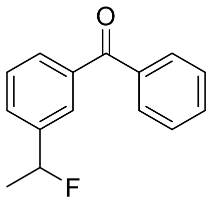

2s was obtained after purification by column chromatography on silica gel (Pentane: Ethyl acetate= 20:1) to yield 30 %, 27 mg, colorless oil (NMR yields 40 %).

^{1}H NMR (300 MHz, $CDCl_3$) δ 7.85 - 7.77 (m, 3H), 7.74 (dq, *J* = 7.52, 1.42 Hz, 1H), 7.64 - 7.56 (m, 2H), 7.54 - 7.45 (m, 3H), 5.70 (dq, *J* = 47.5, 6.4 Hz, 1H), 1.67 (dd, *J* = 23.9, 6.4 Hz, 3H).
^{19}F NMR (282 MHz, $CDCl_3$) δ -168.57 (dq, *J* = 48.1, 24.4 Hz, 1F).
^{13}C{^{1}H} NMR (75 MHz, $CDCl_3$) δ 196.5, 141.9 (d, *J* = 20.0 Hz), 137.6 (d, *J* = 33.9 Hz), 132.6, 130.0 (d, *J* = 10.2 Hz), 129.1 (d, *J* = 6.8 Hz), 128.5 (d, *J* = 11.1 Hz), 126.7 (d, *J* = 6.9 Hz), 90.5 (d, *J* = 169.0 Hz), 23.0 (d, *J* = 24.9 Hz).

(3aS,6R,6aR)-4-fluoro-6-methoxy-2,2-dimethyltetrahydrofuro[3,4-d][1,3]dioxole (2t) [1]

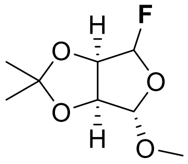

2t was obtained after purification by column chromatography on silica gel (DCM) to yield 29 %, 22 mg, colorless oil (NMR yields 30 %).

^{1}H NMR (300 MHz, $CDCl_3$) δ 5.9 (s, 0.5 H), 5.7 (s, 0.5 H), 5.2 (d, *J* = 2.9 Hz, 1H), 4.8 (m, 1H), 4.7 (d, *J* = 5.7 Hz, 1H), 3.4 (s, 3H), 1.4 (q, *J* = 0.7 Hz, 3H), 1.3 (d, *J* = 0.7 Hz, 3H).

^{19}F NMR (282 MHz, $CDCl_3$) δ -119.4 (d, *J* = 64.3 Hz).

^{13}C{^{1}H} NMR (75 MHz, $CDCl_3$) δ 117.2, 114.2, 113.0, 111.5 (d, *J* = 1.7 Hz), 84.2, 83.7, 83.3, 55.4, 26.2, 24.8.

3-fluoro-4-phenylbutanoic acid (2u) [1]

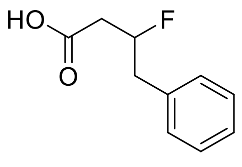

2u was obtained after purification by column chromatography on silica gel (DCM: MeOH = 100:1) to yield 32 %, 23 mg, white solid (NMR yields 46 %).

^{1}H NMR (300 MHz, $CDCl_3$) δ 10.24 (s, 1H), 7.38 - 7.23 (m, 5H), 5.34 - 5.04 (m, 1H), 3.16 - 2.97 (m, 2H), 2.81 - 2.65 (m, 2H).
^{19}F NMR (282 MHz, $CDCl_3$) δ -177.82 (s, 1F).
^{13}C{^{1}H} NMR (75 MHz, $CDCl_3$) δ 175.7 (d, *J* = 5.5 Hz), 136.0 (d, *J* = 5.1 Hz), 129.5, 128.6, 126.9, 122.3, 91.4, 89.6, 41.9 (d, *J* = 21.2 Hz), 39.5 (d, *J* = 23.9 Hz).

5. Mechanistic studies

5.1. ON/OFF experiment

A 25 mL Schlenk tube, previously oven-dried and equipped with a magnetic stir bar, was charged with N-Phthaloylglycine (0.1 mmol, 20.5 mg) and Selectfluor (212.55 mg, 6 equiv.). $CeCl_3$ (10 mol%, 2.46 mg) was added in to Schlenk tube. Degassed MeCN (0.5 mL), degassed H_2O (0.5 mL), and 2,6-lutidine (23 μL, 2 equiv.) were introduced via syringe. The mixed reaction solution was frozen three times to remove oxygen, and the reaction mixture was stirred and irradiated for three hours under the Ar in the dark or irradiated with 390 nm LEDs light for varying durations while being cooled with a fan. Upon completion, the reaction mixture was washed with water and extracted with DCM (3 × 10 mL). The combined organic layers were dried over Na_2SO_4, filtered, and passed through a short silica plug using DCM to remove iron species. After concentration by rotary evaporation, the crude yield of product 1 was determined by ^{19}F NMR using p-fluorotoluene as an internal standard.

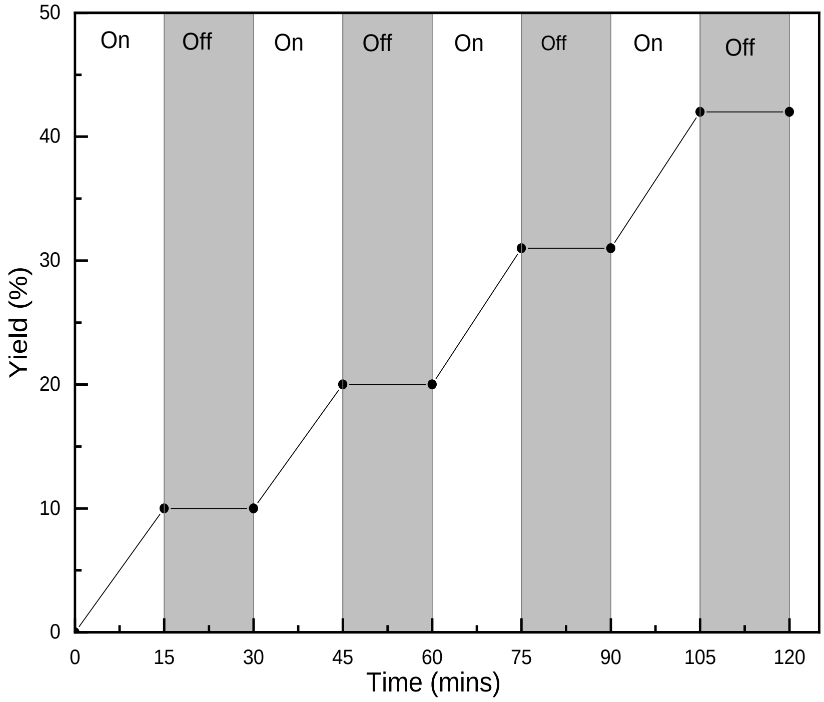

Figure S1 ON/OFF experiment

5.2. *In-situ* Infrared Spectroscopy

We opted for *In-situ* ATR-IR spectroscopy for the detection of CO_2 evolution during the progression of reaction. Data was collected using the spectral window of 2400 to 1200 cm^{-1} sampled in 60 second intervals. In a Schlenk tube N-Phthaloylglycine (0.1 mmol, 20.5 mg)

and Selectfluor (212.55 mg, 6 equiv.). $CeCl_3$ (10 mol%, 2.46 mg) was added. Degassed MeCN (0.5 mL), degassed H_2O (0.5 mL), and 2,6-lutidine (23 µL, 2 equiv.) were introduced via syringe Then, diamond probe was inserted in reaction mixture and in-situ IR spectra were recorded for 5 hr at 60 second intervals. As illustrated in Figure S1, the signal intensity at 2342 cm^{-1}, corresponding to the asymmetric stretching of CO_2, progressively increases as the reaction proceeds. Other then this there were two other signals which were highly observable.

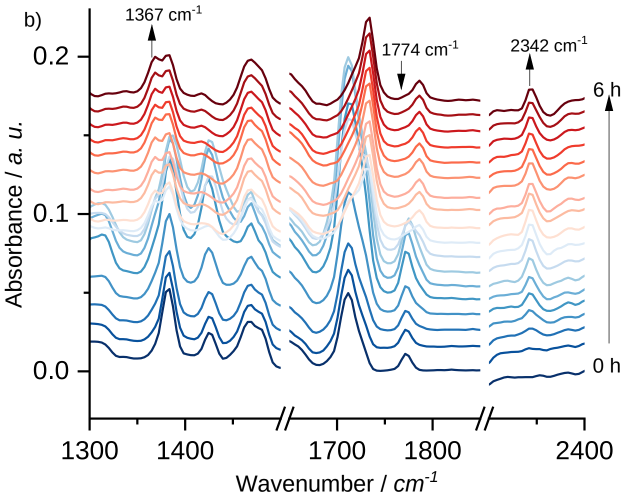

Figure S2: *In-situ* ATR-IR spectrum of reaction mixture

As shown in Figure S3, the peak at 1774 cm^{-1}, corresponding to the asymmetric stretching of the carboxyl group, initially increases and then decreases over time. This trend suggests that the substrate undergoes deprotonation by the base, leading to the formation of the carboxylate anion (R-COO^{-}). Over the course of the reaction, this signal diminishes due to the evolution of CO_2, which progressively increases and eventually reaches saturation. Another key signal is observed at 1367 cm^{-1}, corresponding to the formation of the C–F bond in the product. This peak intensifies over time and stabilizes after approximately three hours, indicating the completion of the reaction within this timeframe.

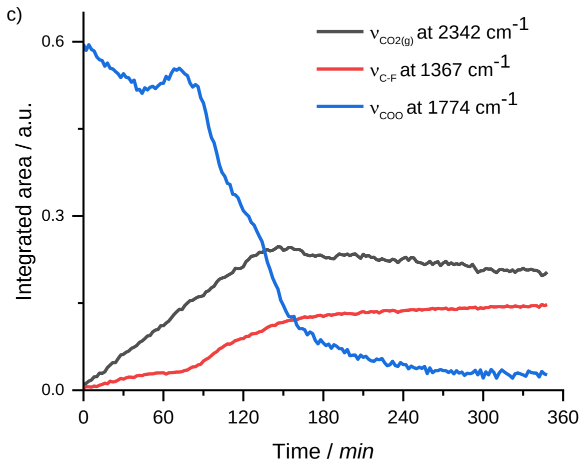

Figure S3: *In-situ* ATR-IR spectrum for individual signal area over time

5.3. UV-vis Spectroscopy

To investigate whether the interaction between carboxylic acids and cerium(IV) facilitates a ligand-to-metal charge transfer (LMCT) process, leading to the reduction of Ce(IV) to Ce(III), $(n\text{-}Bu_4N)_2CeCl_6$ was selected as the Ce(IV) source. This choice ensured sufficient solubility in organic solvents, facilitating species detection. UV-Vis spectra were recorded in the 200–500 nm range to monitor this transformation.

Synthesis of $(n\text{-}Bu_4N)_2CeCl_6$[5]

In an oven-dried round-bottom flask equipped witha a Teflon-coated magnetic stir bar, $Ce(SO_4)_2{\cdot}(H_2O)_n$ (1.8 g, 5.8 mmol, 1.0 equiv.) and tetrabutylammonium chloride (TBACl, 3.22 g, 11.7 mmol, 2.0 equiv.) were added. Concentrated hydrochloric acid (37%, 15.0 mL) was then added dropwise to the mixture at room temperature. Upon formation of a yellow-orange precipitate, an additional portion of TBACl (324 mg, 1.2 mmol, 0.1 equiv.) was introduced, and stirring was continued for 20.0 minutes.

The resulting suspension was cooled to 5.0 °C using an ice-water bath. The solid product was collected by suction filtration through a sintered glass funnel, washed thoroughly (at least three times) with minimal cold acetone, and dried under high vacuum to afford an intensely yellow powder (520 mg, 0.57 mmol, 11% yield).

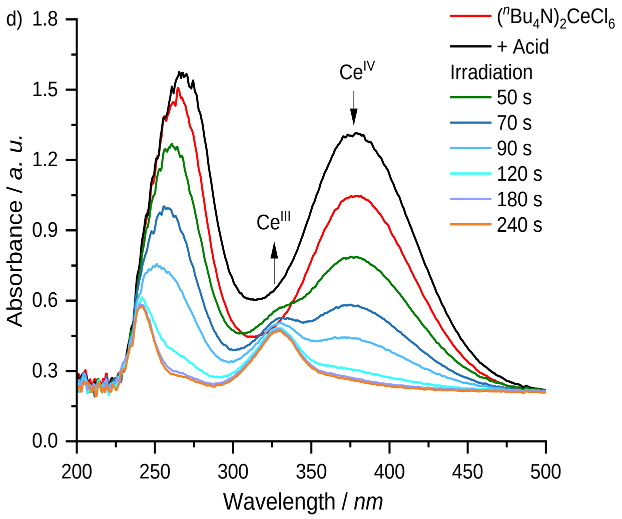

Figure S4 Uv-Vis spectra of the solution of Ce^{IV} catalyst and **1a** under the irradiation of light for the given interval of time.

The UV-Vis spectra of a Ce^{IV} catalyst solution were recorded to examine its photoreduction behavior (Figure S4). For the first solution, Ce^{IV} catalyst (2.2 mg, 2.6 μmol) was dissolved in 4 mL MeCN, and its UV-Vis spectra under 390 nm light irradiation displayed an absorption peak characteristic of Ce^{IV}, with no observable reduction. However, upon addition of N-

Phthaloylglycine (1a, 0.4 mmol, 84 mg) to the same solution, followed by irradiation at 390 nm with 15-second intervals, a rapid reduction of Ce^{IV} to Ce^{III} was observed. The absorption peak at 378 nm gradually diminished, while a new absorption band at 330 nm appeared, confirming the formation of Ce^{III}. This spectral shift strongly supports the interaction between the Ce^{IV} catalyst and carboxylic acids via the LMCT process.

6. NMR spectra

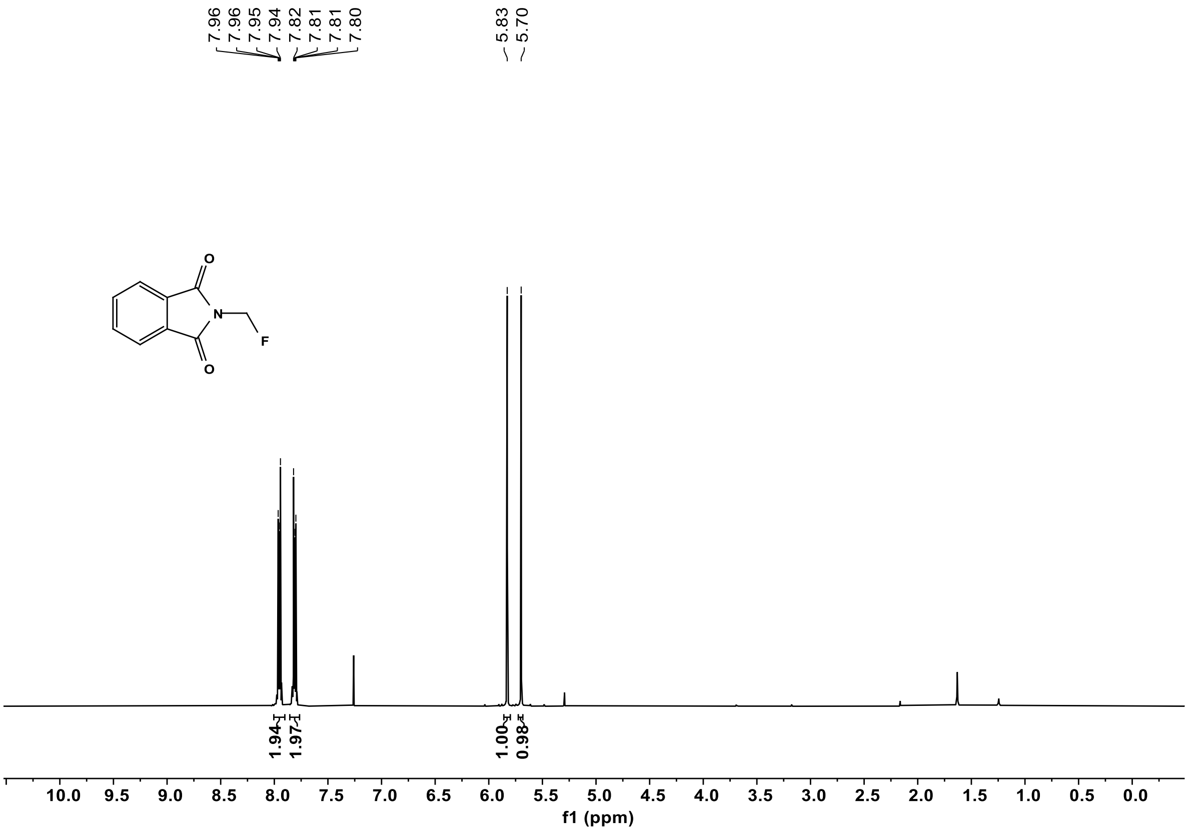

^{1}H NMR spectrum of compound **2a** (400 MHz, $CDCl_3$)

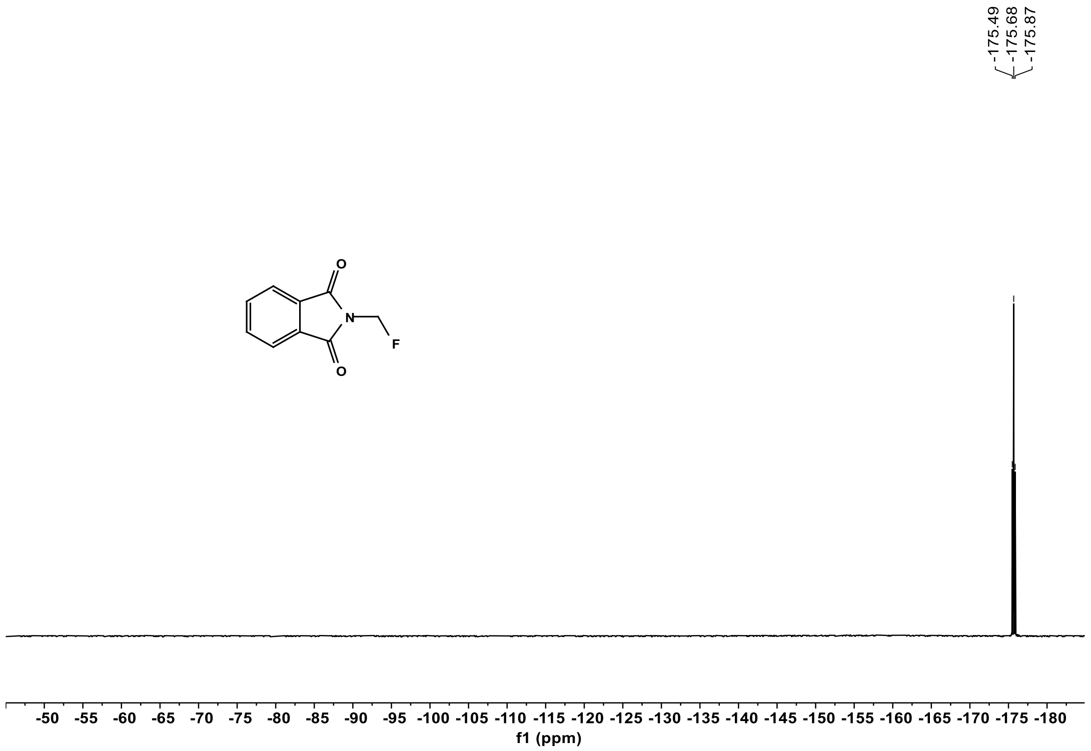

^{19}F NMR spectrum of compound **2a** (282 MHz, $CDCl_3$)

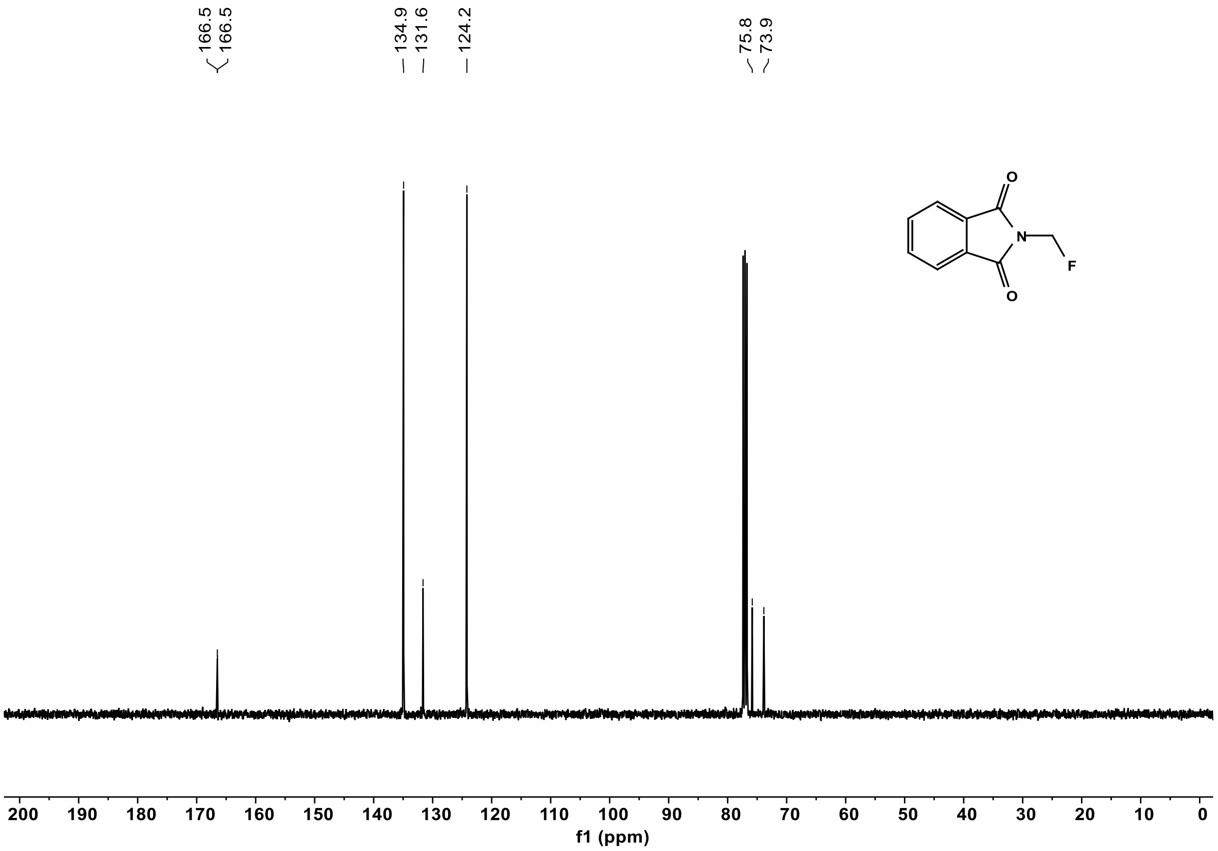

$^{13}C\{^{1}H\}$ NMR spectrum of compound 2a (75 MHz, $CDCl_3$)

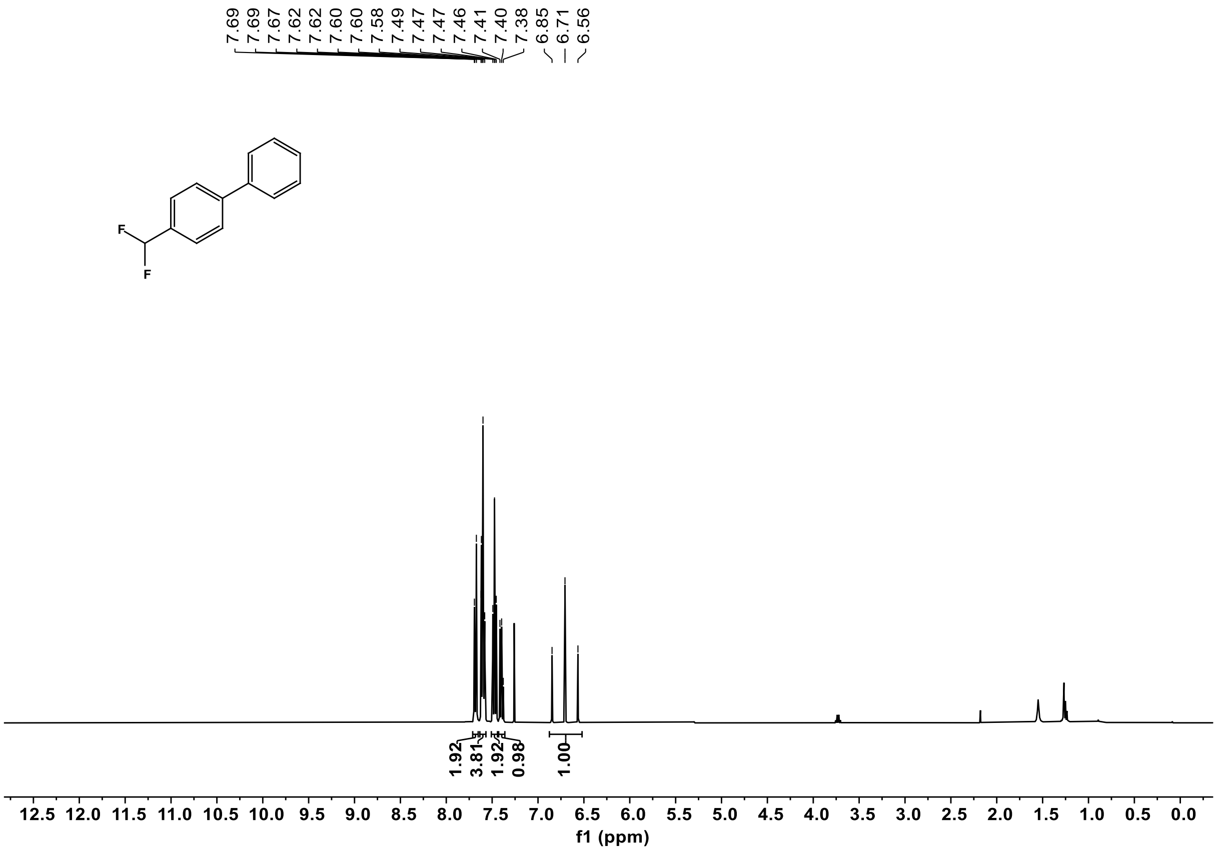

^{1}H NMR spectrum of compound **2b** (400 MHz, $CDCl_3$)

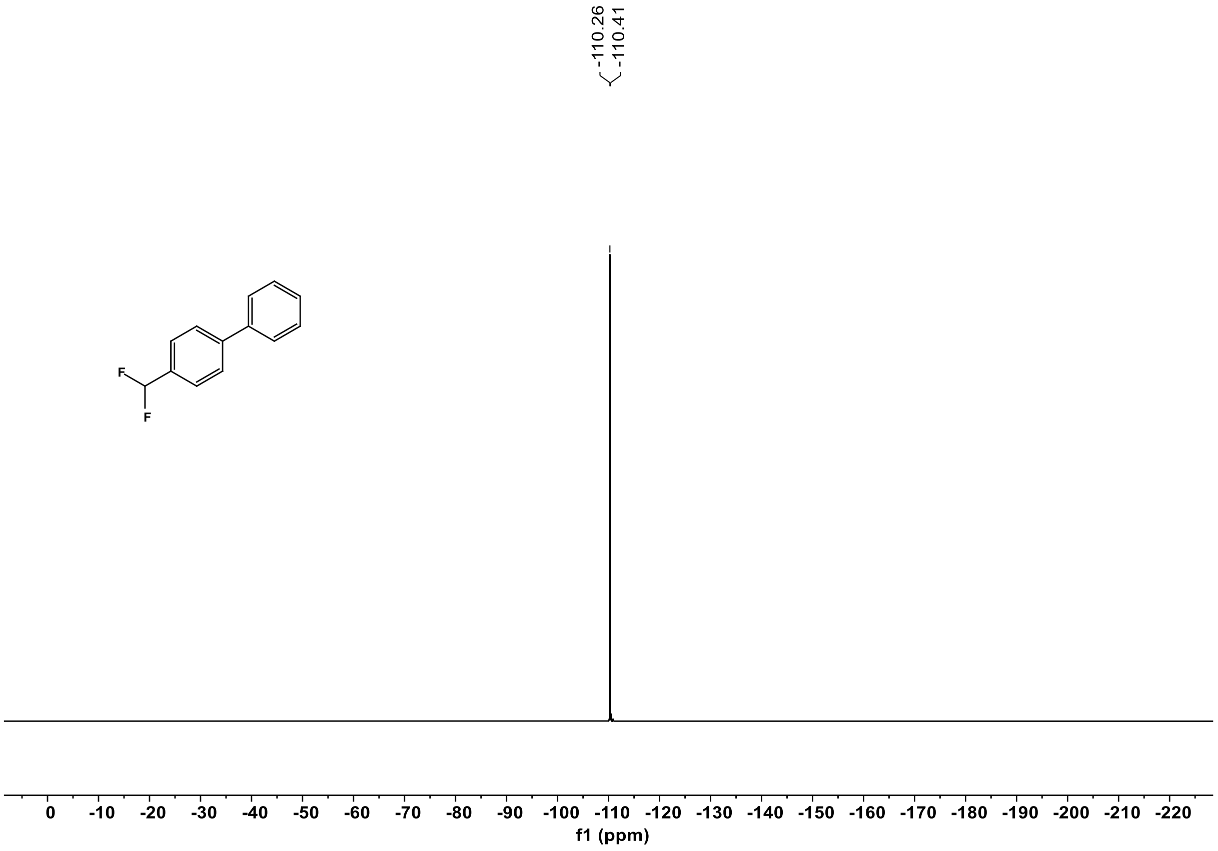

^{19}F NMR spectrum of compound **2b** (376 MHz, $CDCl_3$)

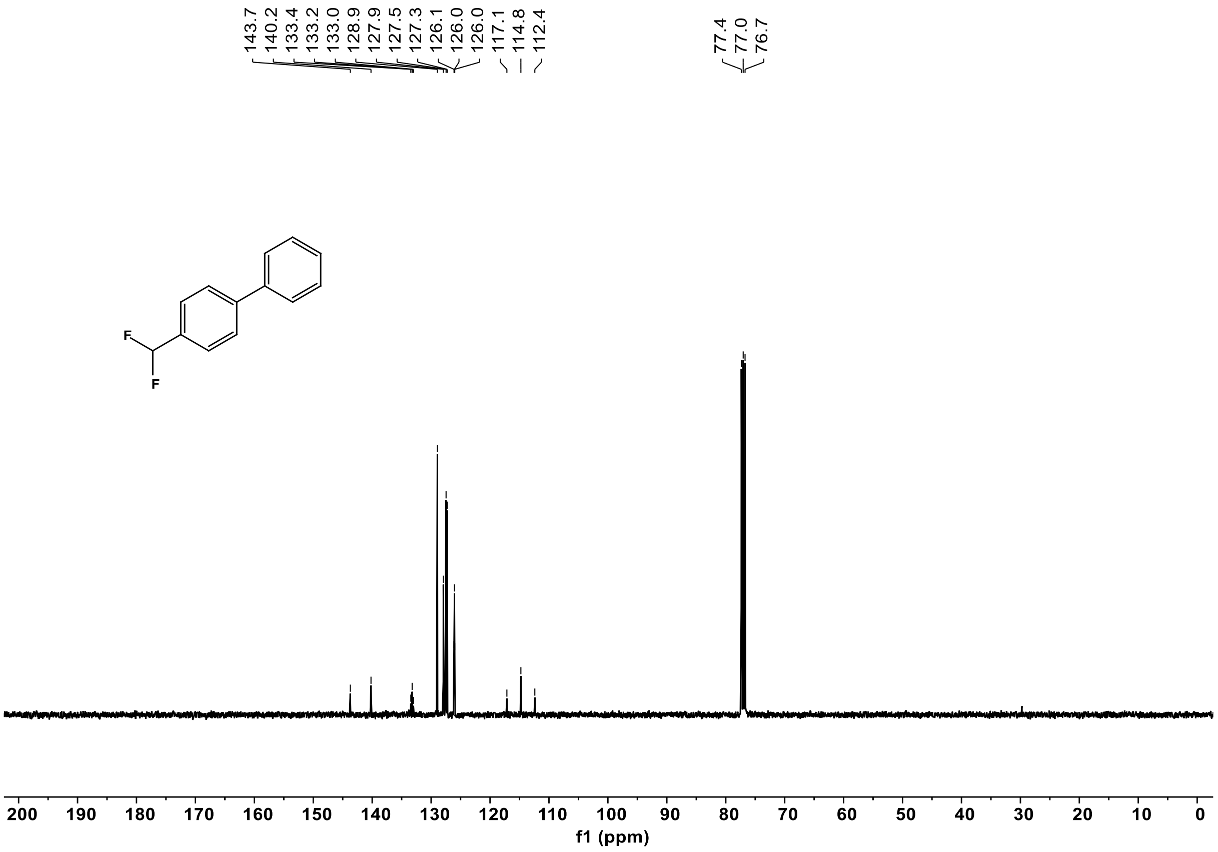

$^{13}C\{^1H\}$ NMR spectrum of compound **2b** (101 MHz, $CDCl_3$)

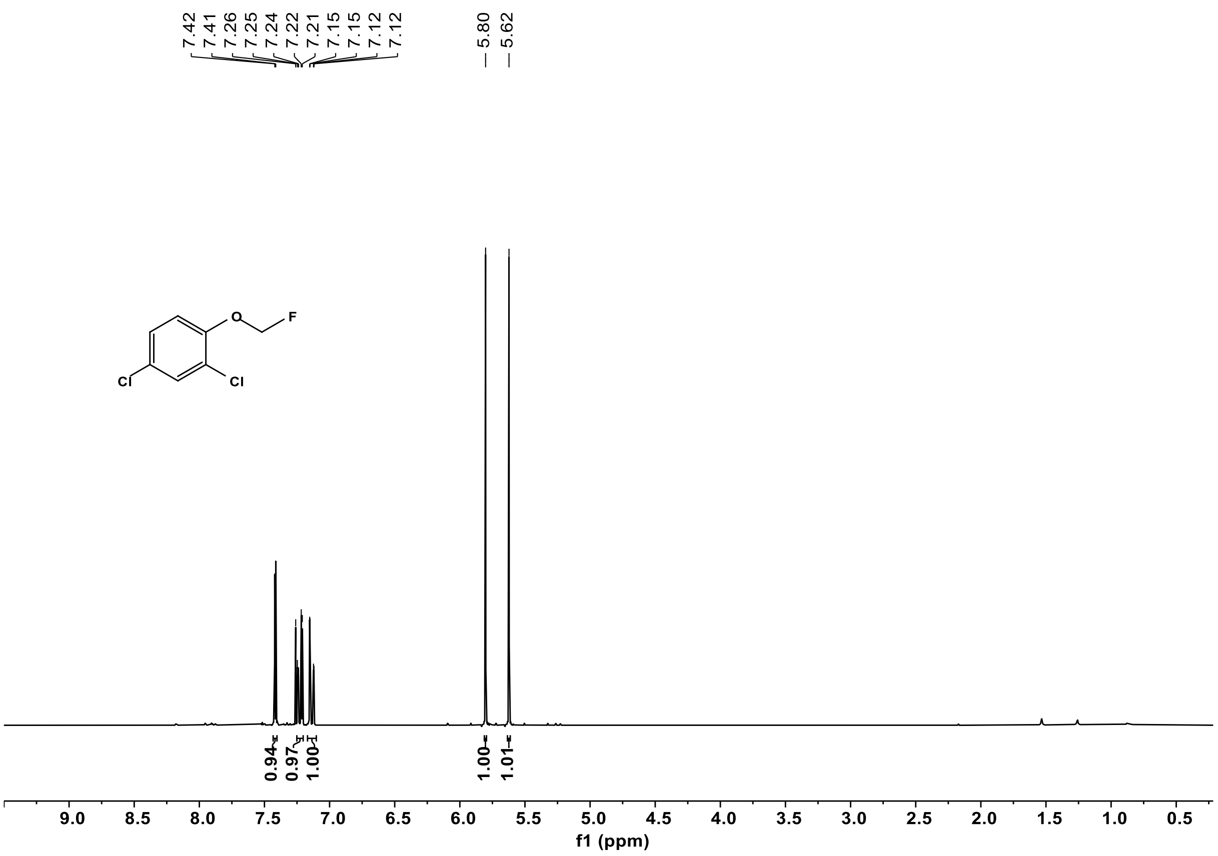

^{1}H NMR spectrum of compound **2c** (300 MHz, $CDCl_3$)

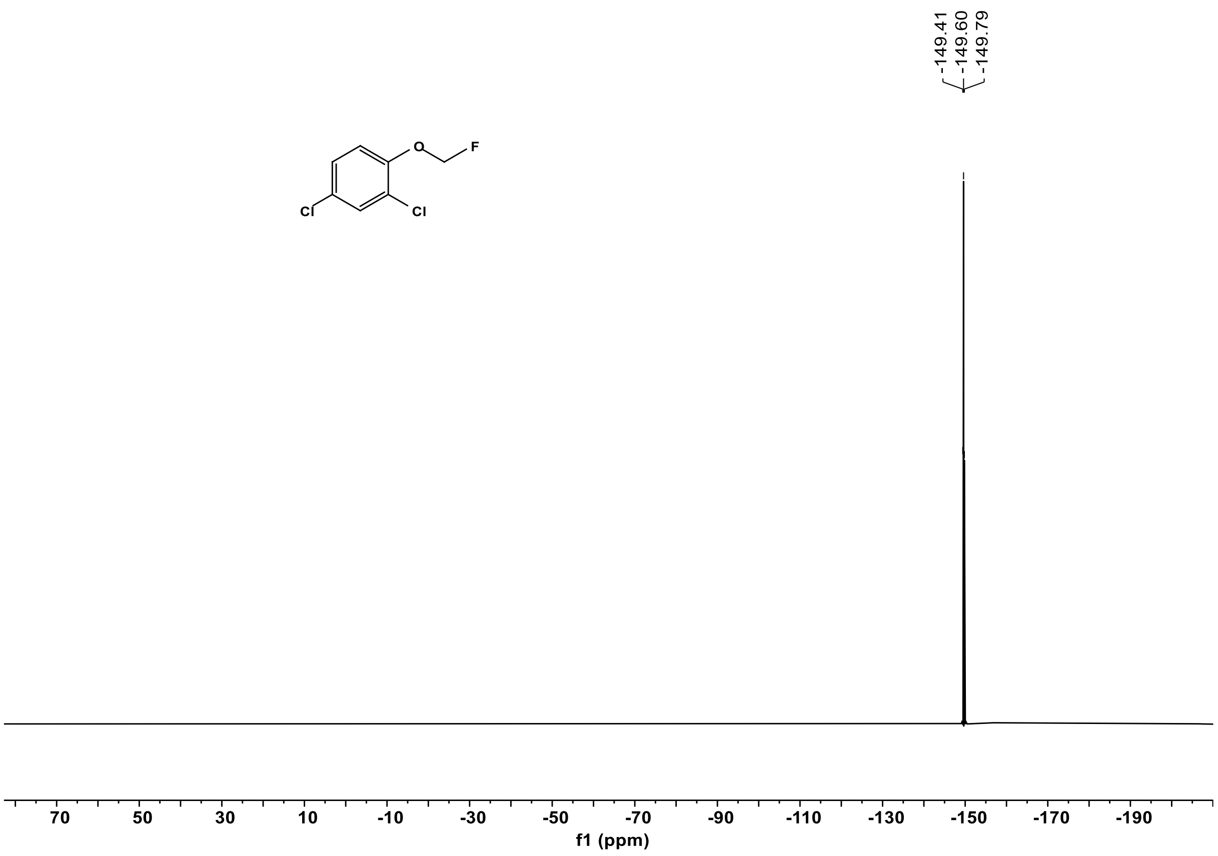

^{19}F NMR spectrum of compound **2c** (282 MHz, $CDCl_3$)

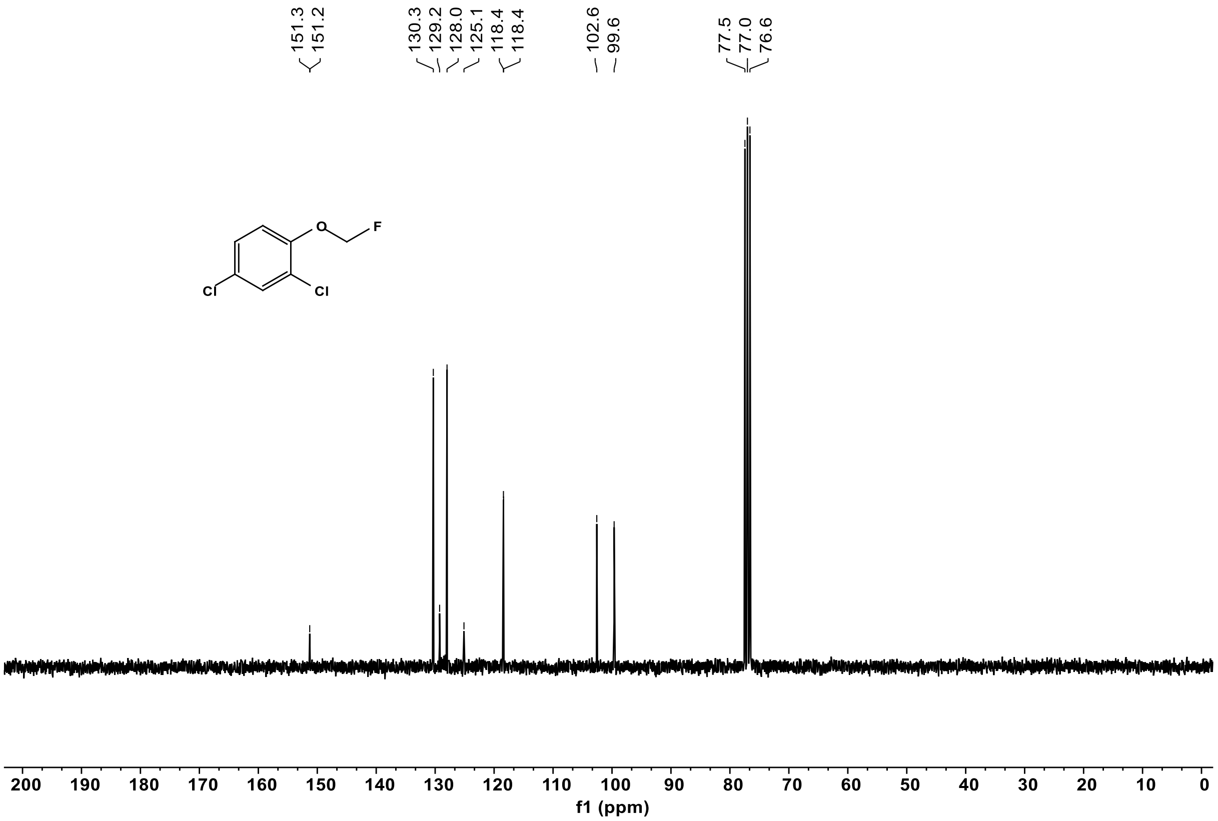

$^{13}C\{^{1}H\}$ NMR spectrum of compound **2c** (75 MHz, $CDCl_3$)

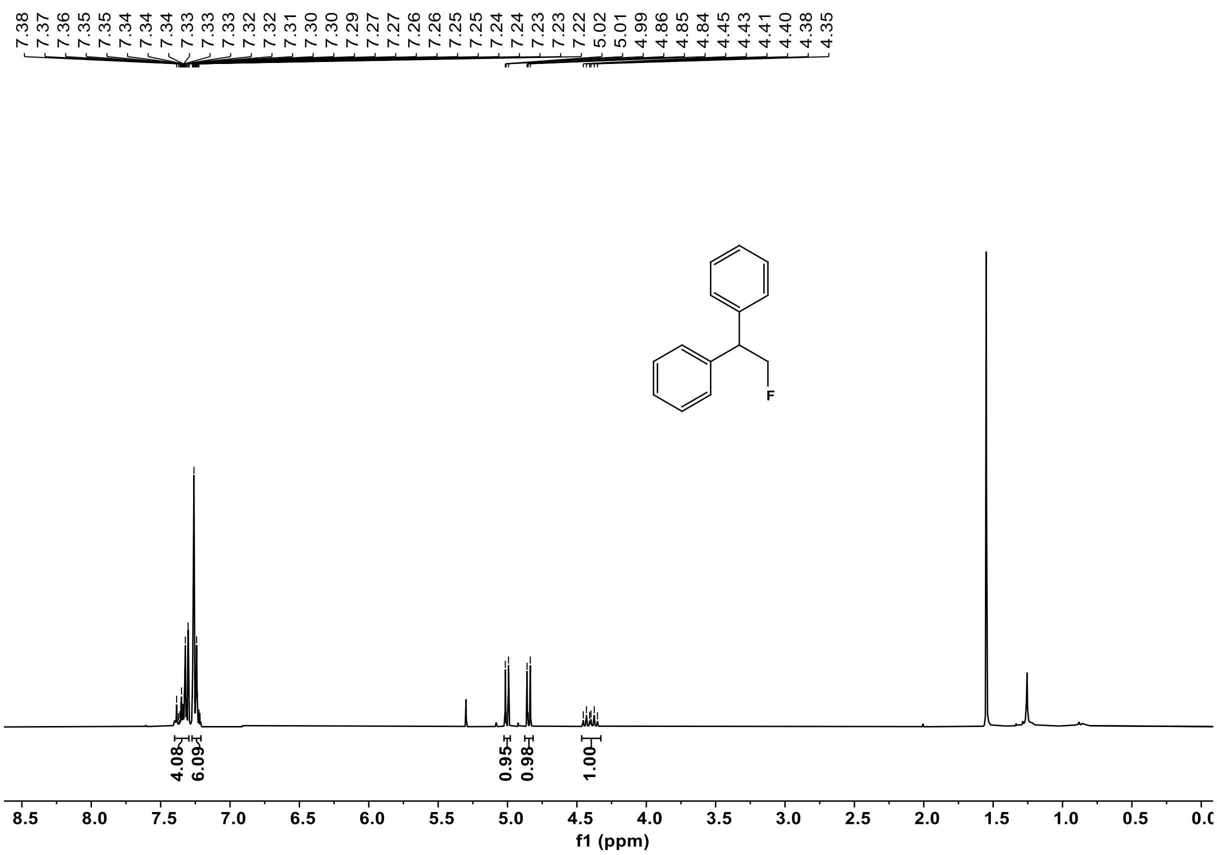

^{1}H NMR spectrum of compound **2d** (300 MHz, $CDCl_3$)

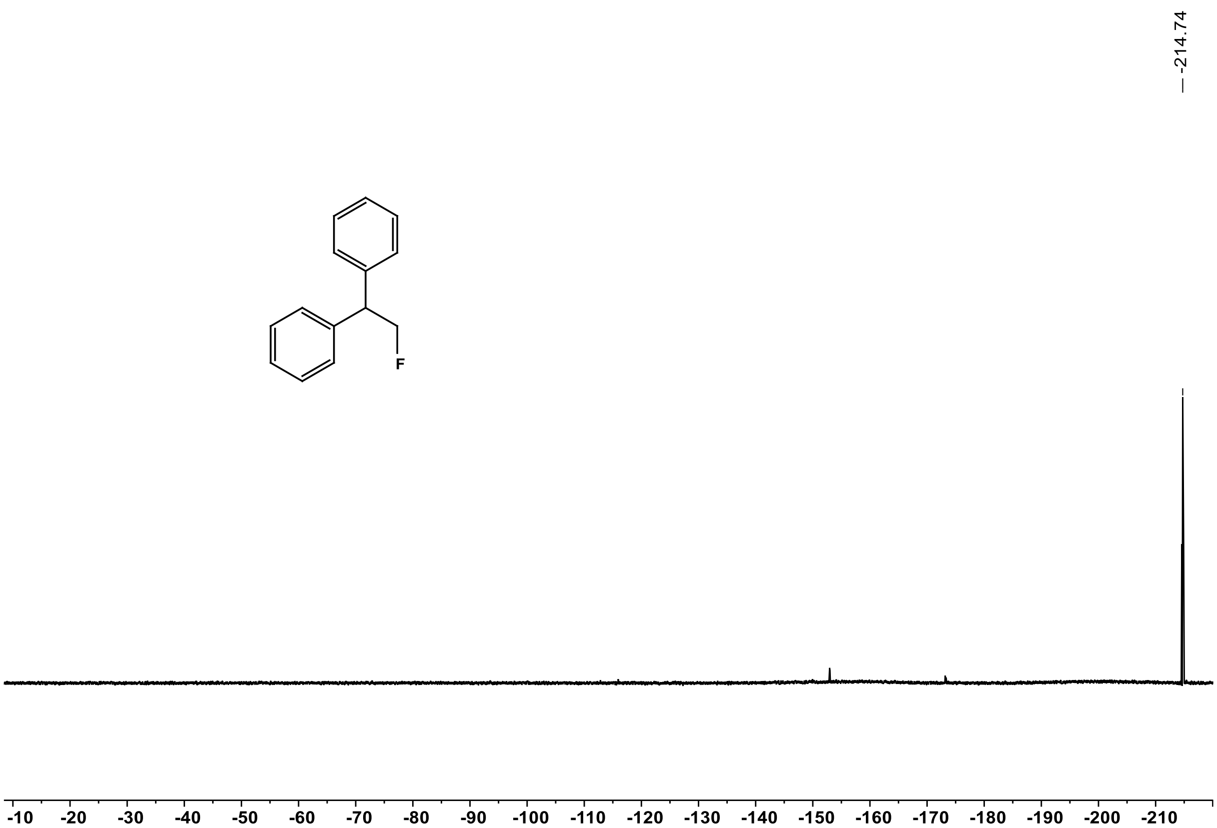

^{19}F NMR spectrum of compound **2d** (282 MHz, $CDCl_3$)

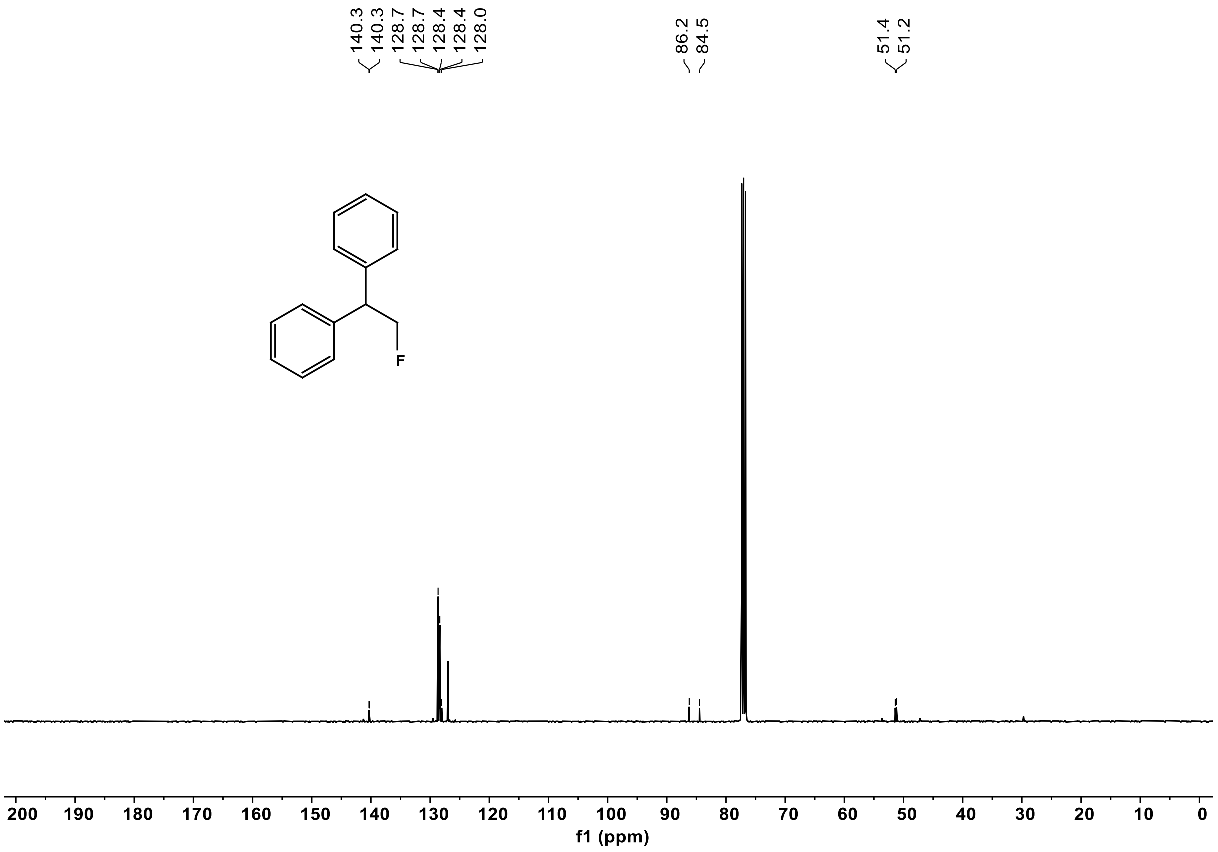

$^{13}C\{^{1}H\}$ NMR spectrum of compound 2d (101 MHz, $CDCl_3$)

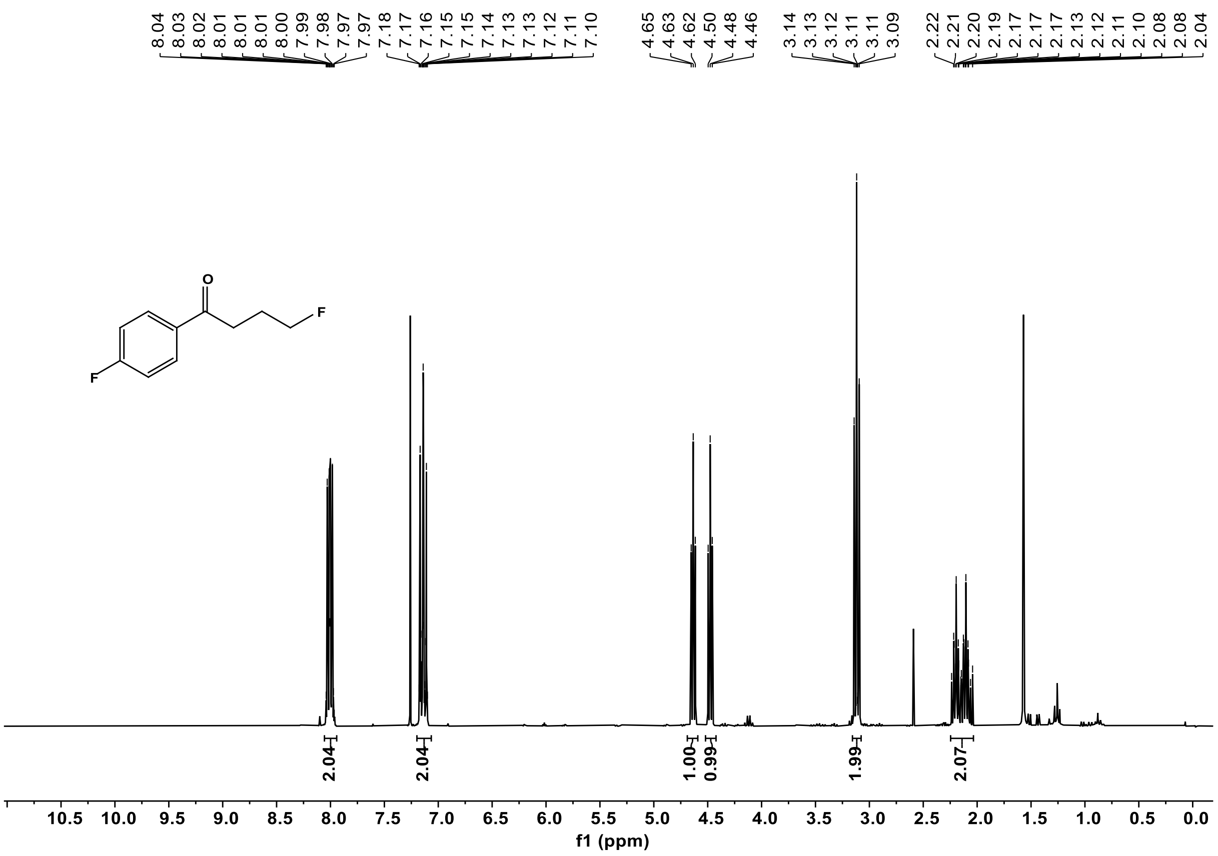

^{1}H NMR spectrum of compound **2e** (300 MHz, $CDCl_3$)

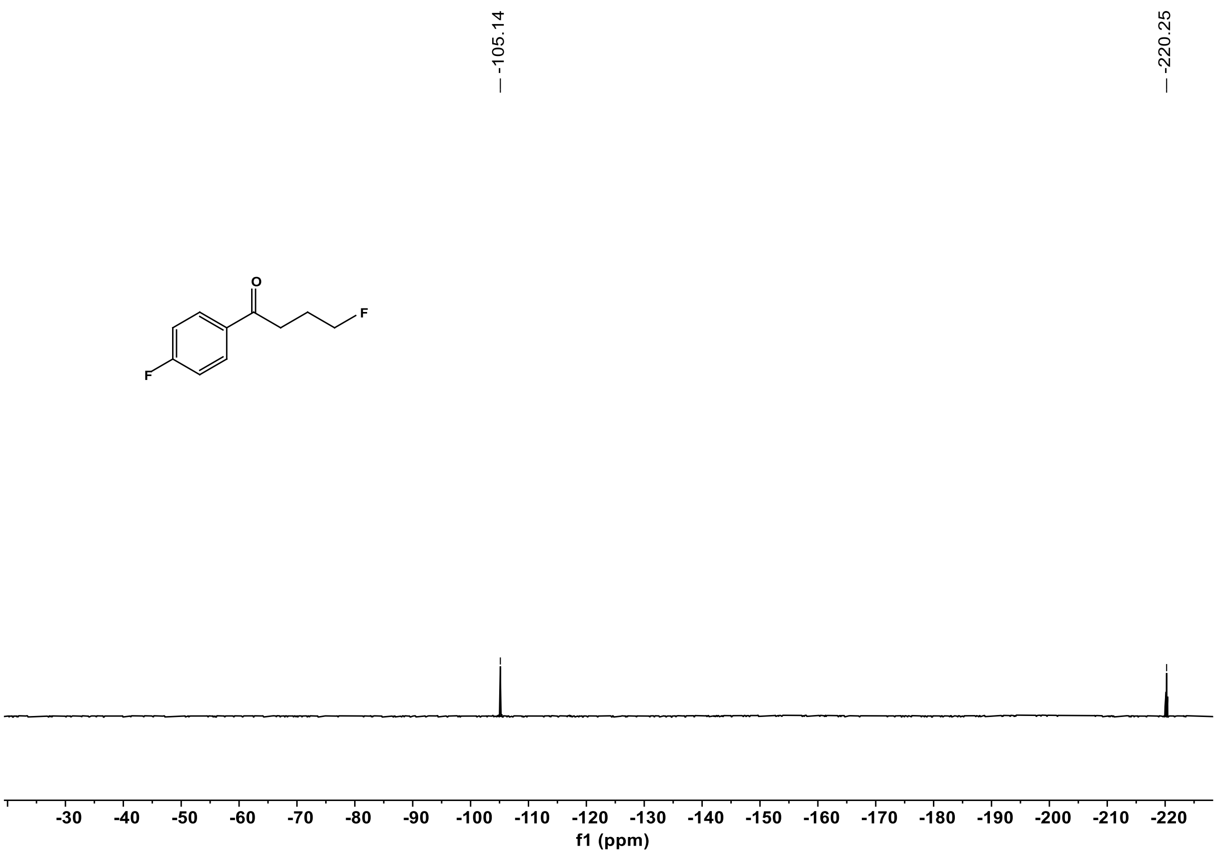

^{19}F NMR spectrum of compound **2e** (282 MHz, $CDCl_3$)

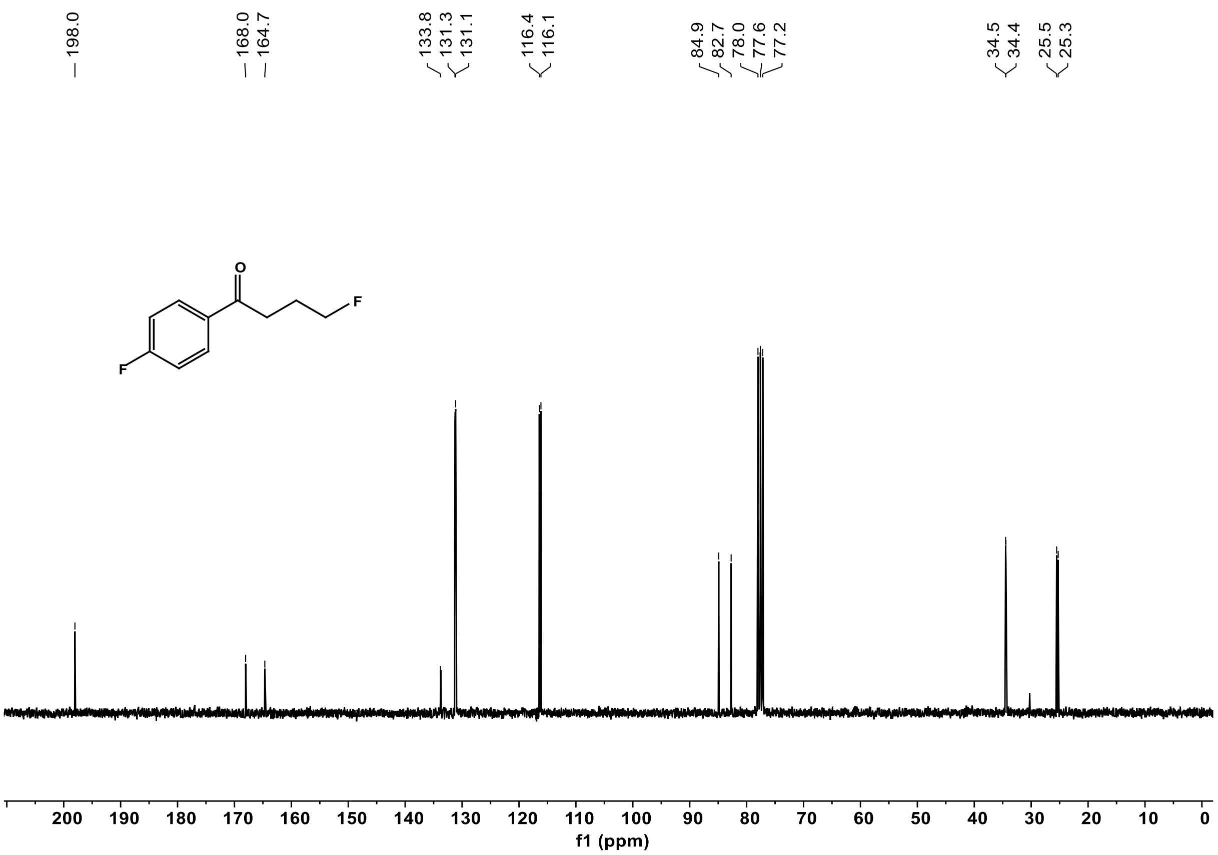

$^{13}C\{^{1}H\}$ NMR spectrum of compound **2e** (75 MHz, $CDCl_3$)

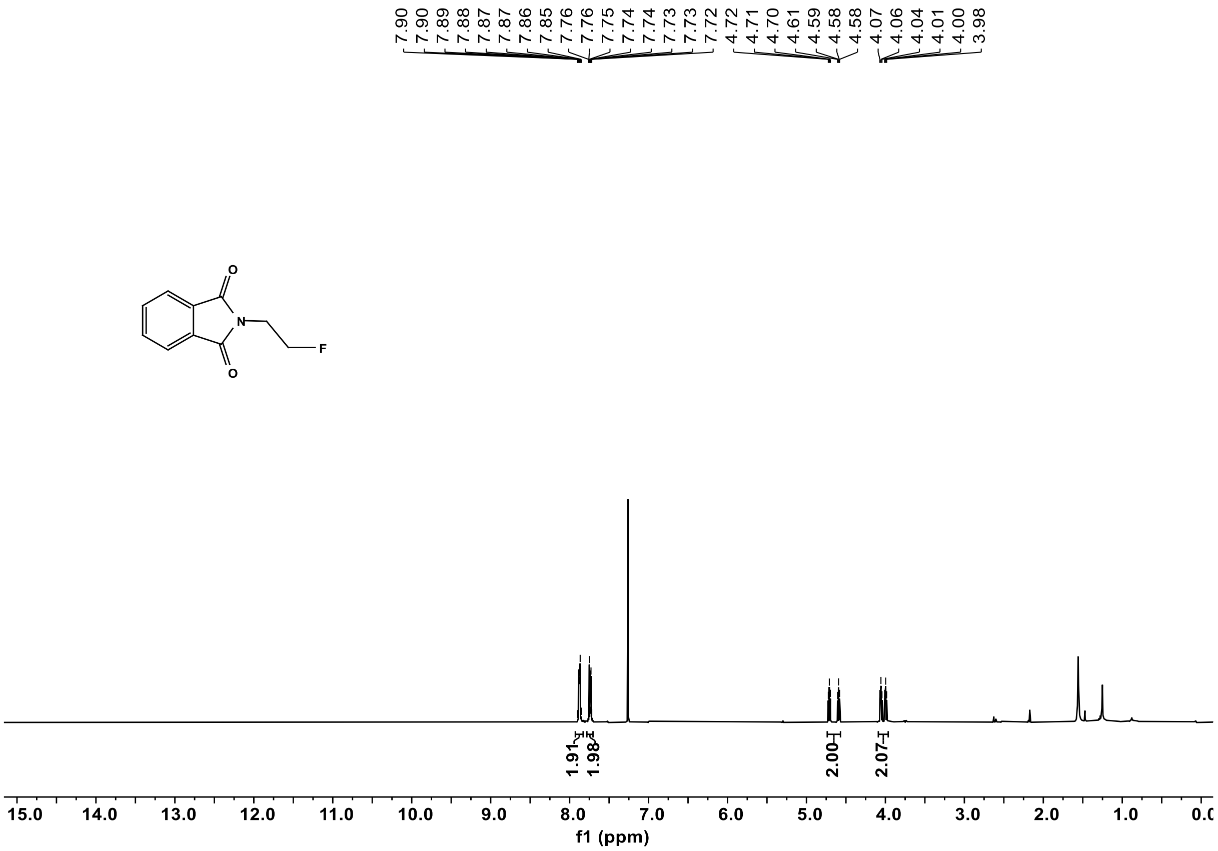

^{1}H NMR spectrum of compound **2f** (400 MHz, $CDCl_3$)

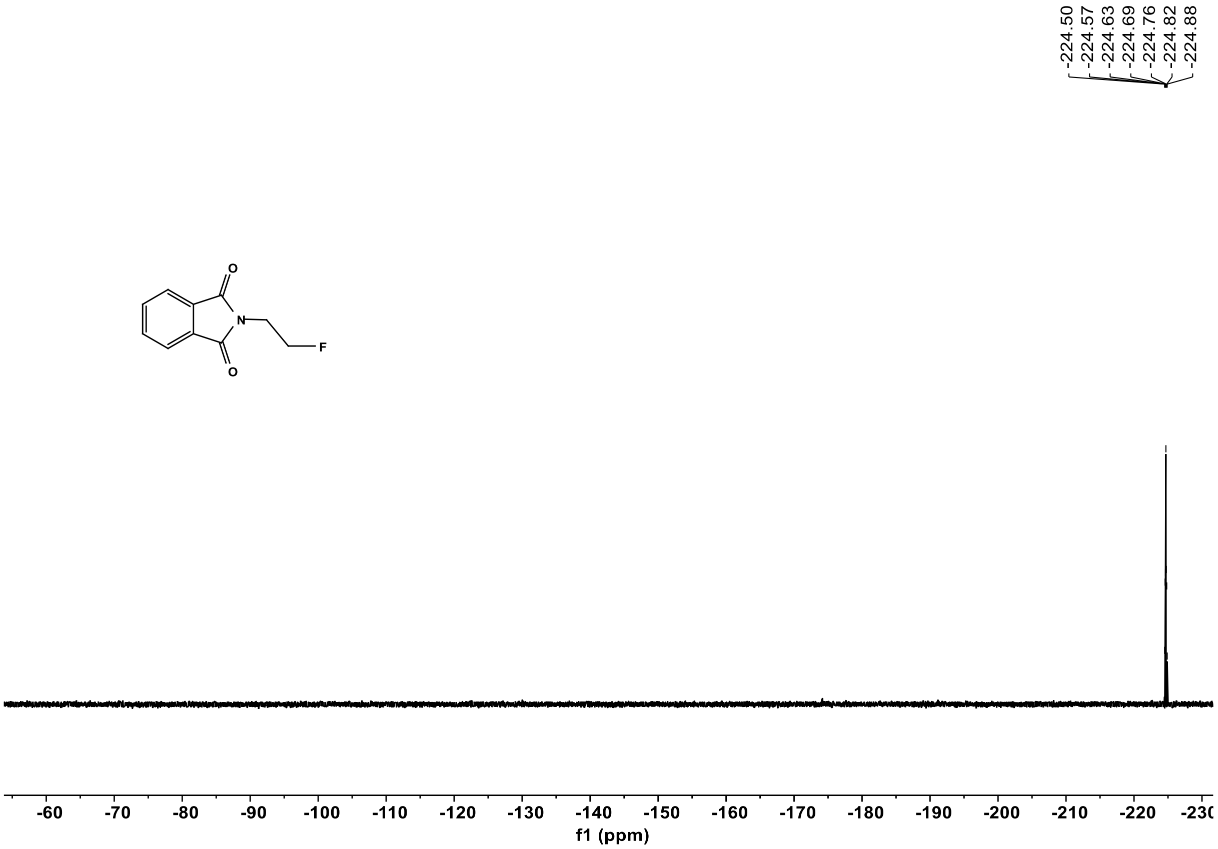

^{19}F NMR spectrum of compound **2f** (376 MHz, $CDCl_3$)

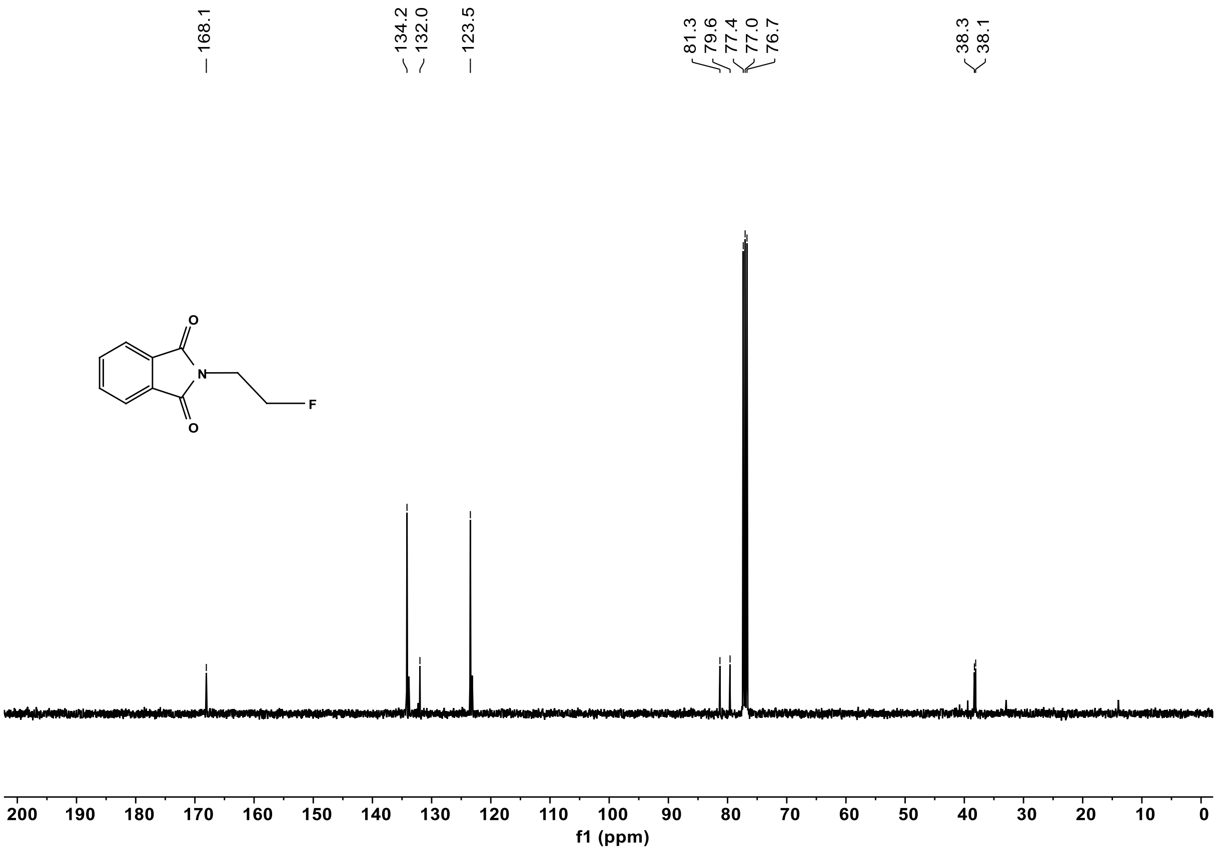

$^{13}C\{^{1}H\}$ NMR spectrum of compound **2f** (101 MHz, $CDCl_3$)

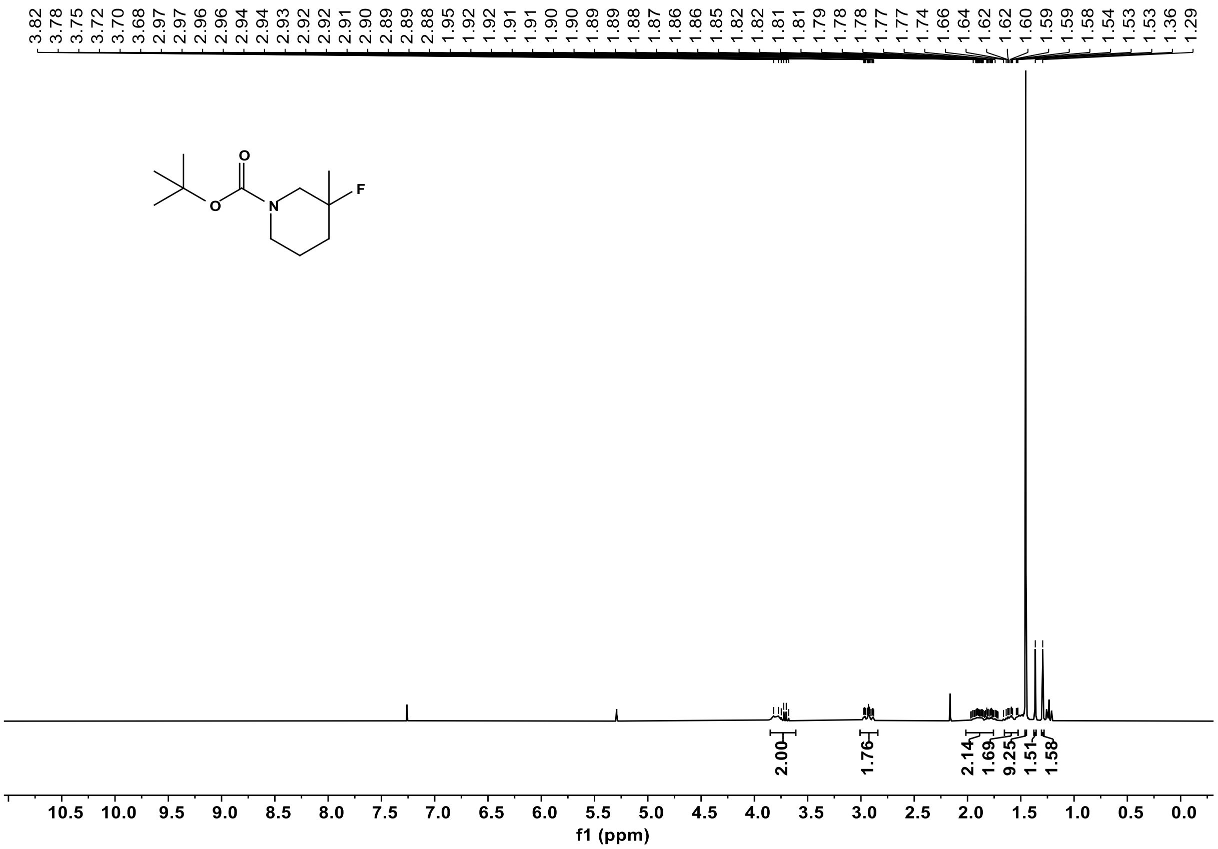

^{1}H NMR spectrum of compound **2h** (300 MHz, $CDCl_3$)

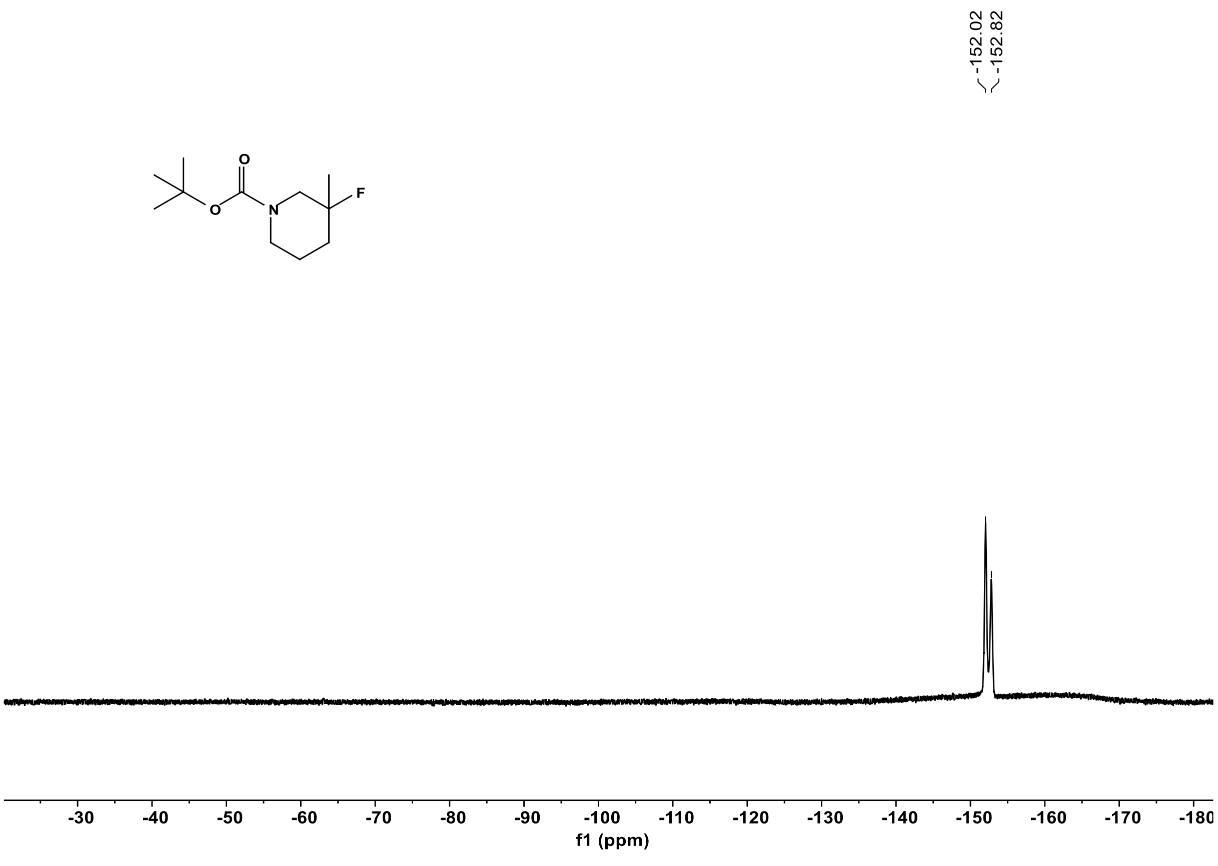

^{19}F NMR spectrum of compound **2h** (282 MHz, $CDCl_3$)

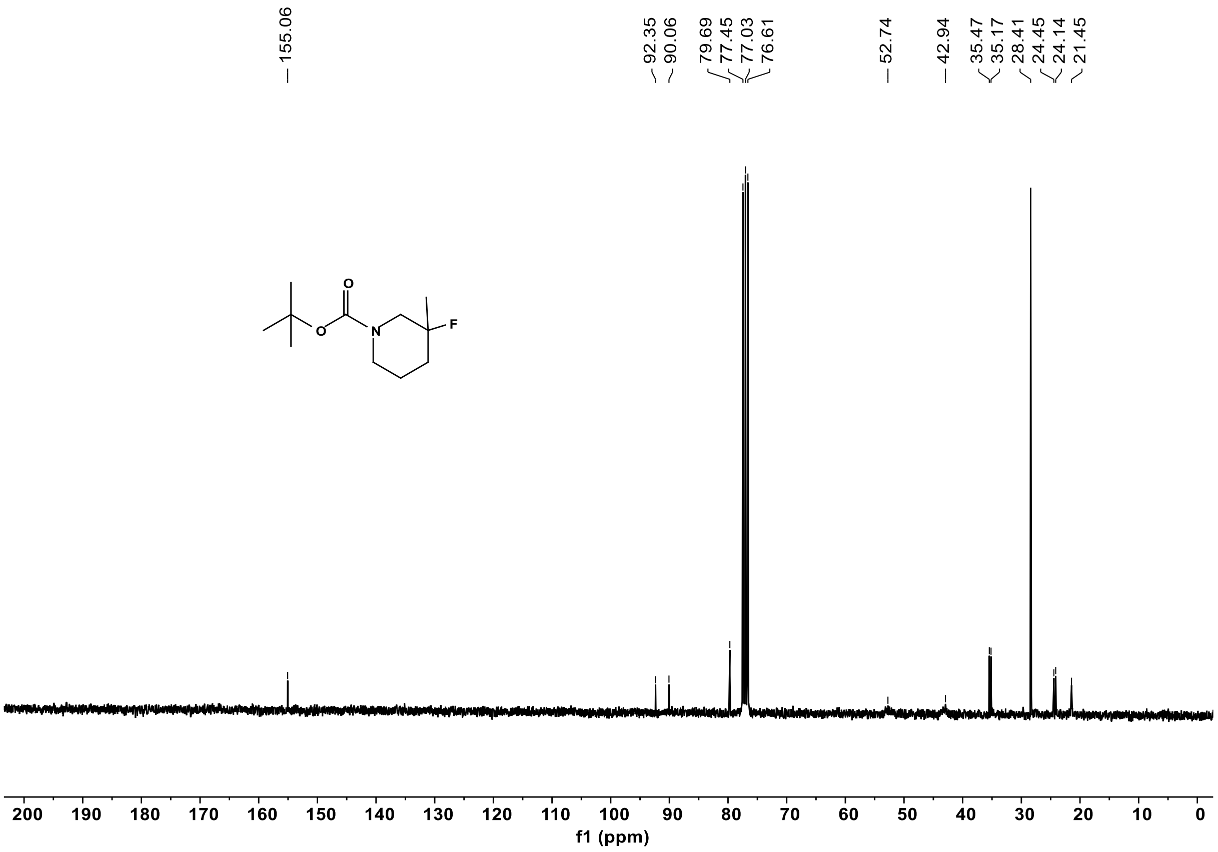

$^{13}C\{^{1}H\}$ NMR spectrum of compound **2h** (75 MHz, $CDCl_3$)

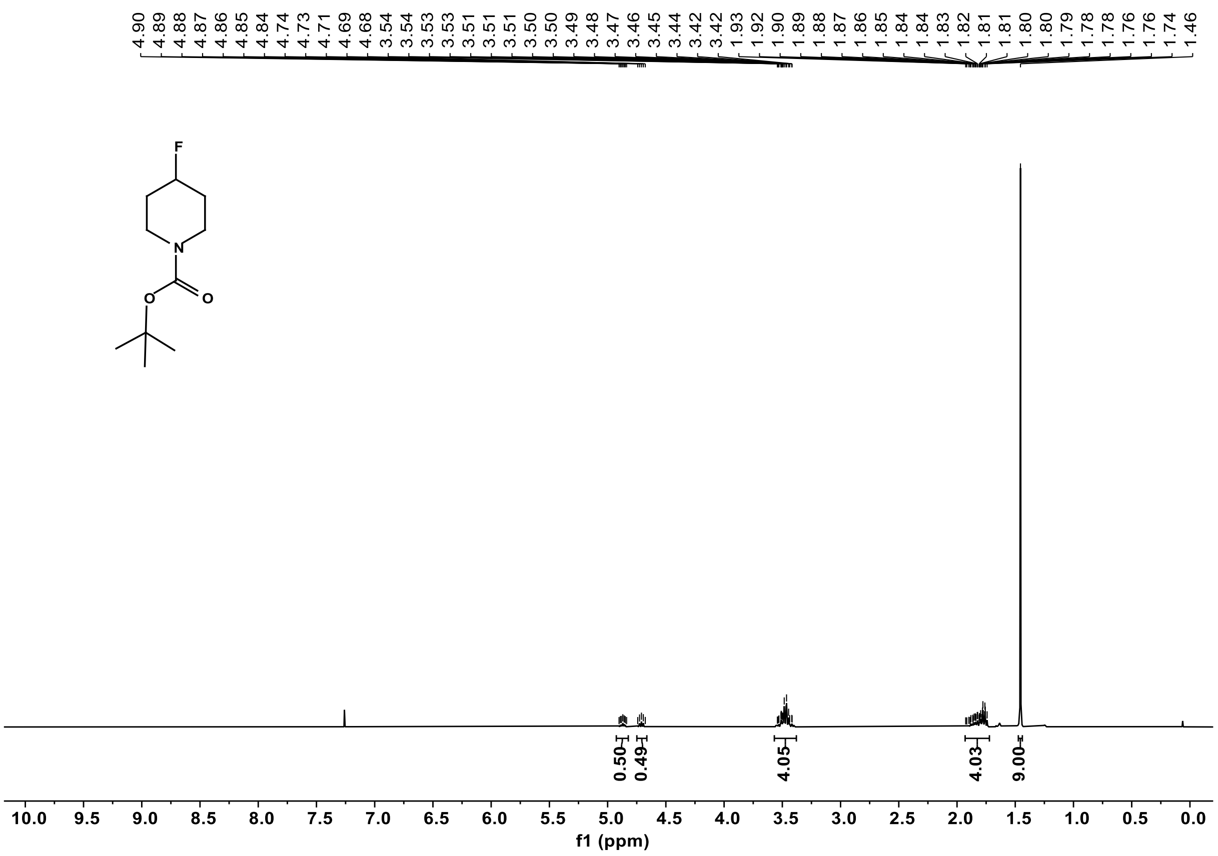

^{1}H NMR spectrum of compound **2i** (300 MHz, $CDCl_3$)

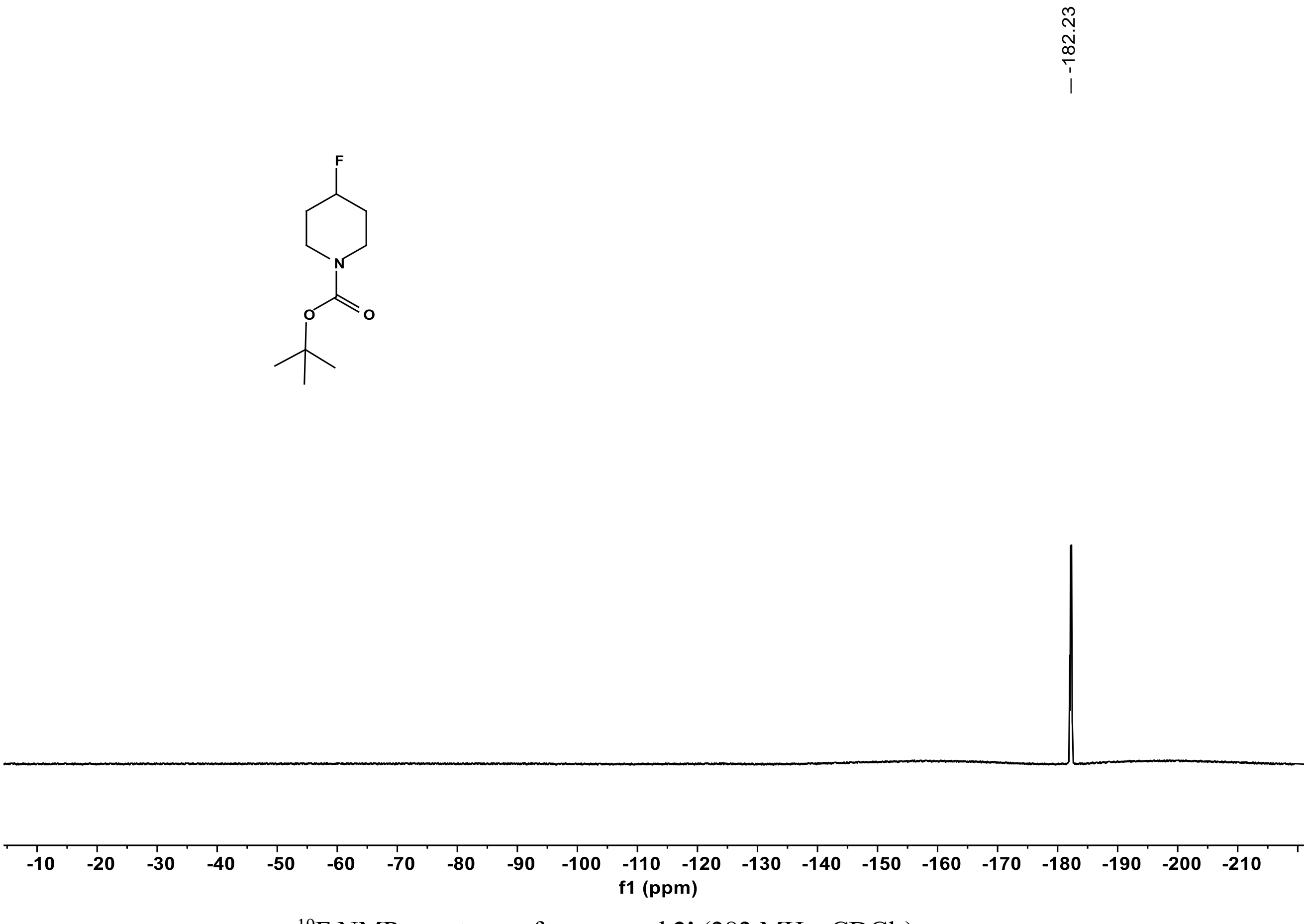

^{19}F NMR spectrum of compound **2i** (282 MHz, $CDCl_3$)

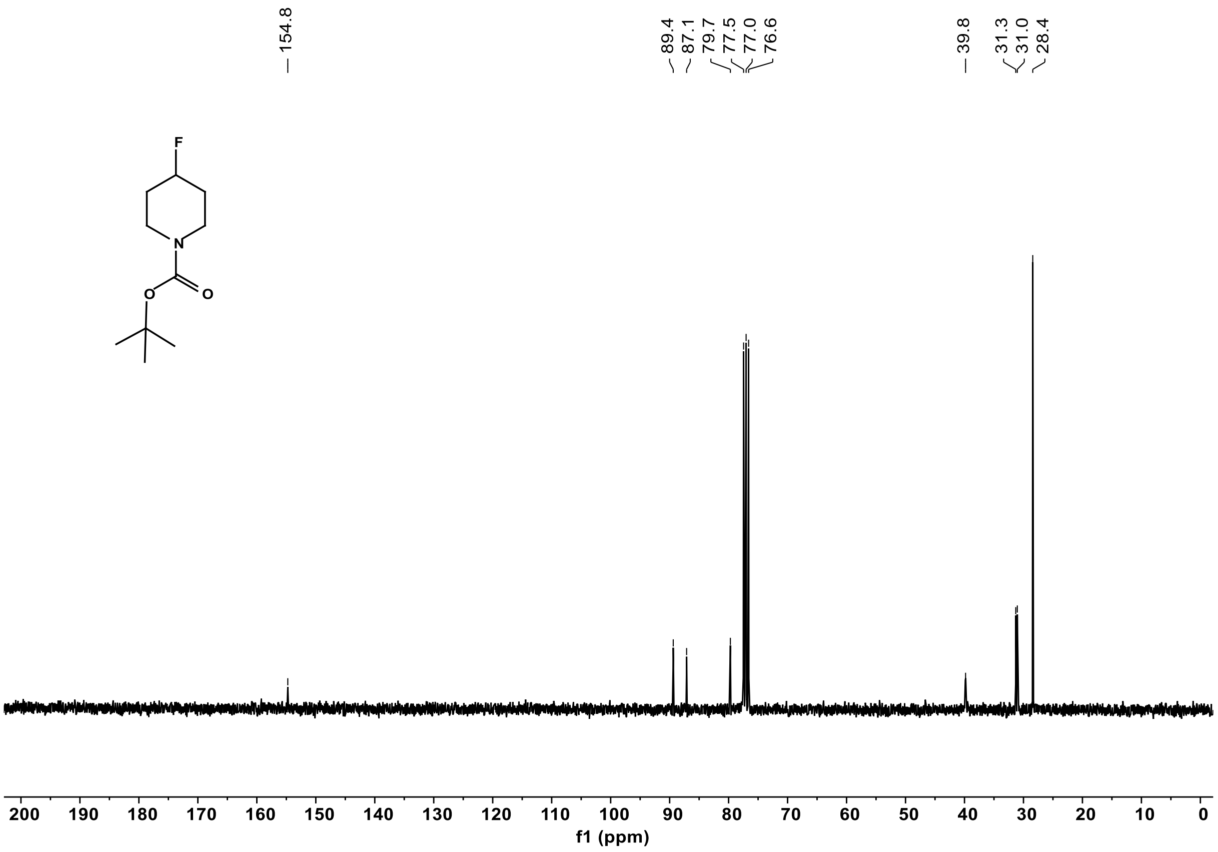

$^{13}C\{^{1}H\}$ NMR spectrum of compound **2i** (75 MHz, $CDCl_3$)

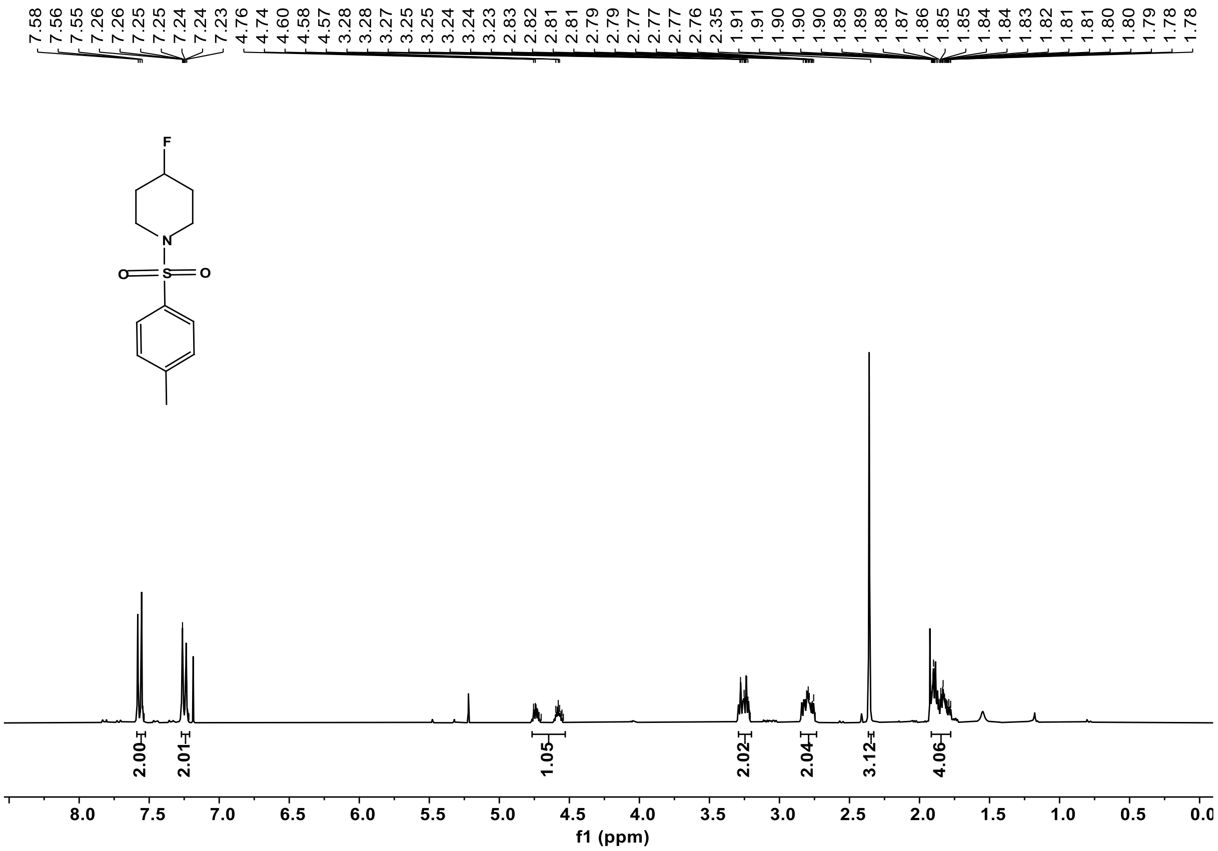

^{1}H NMR spectrum of compound **2J** (300 MHz, $CDCl_3$)

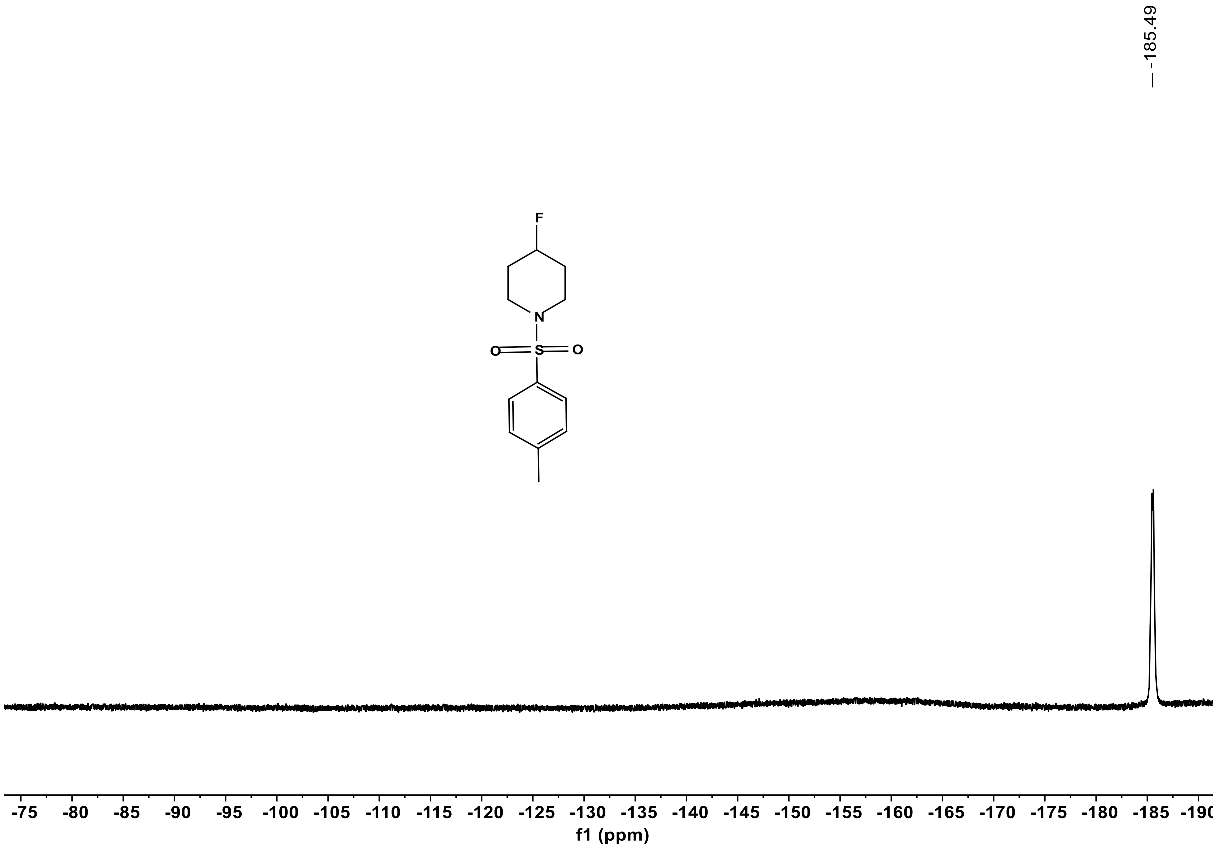

^{19}F NMR spectrum of compound **2J** (282 MHz, $CDCl_3$)

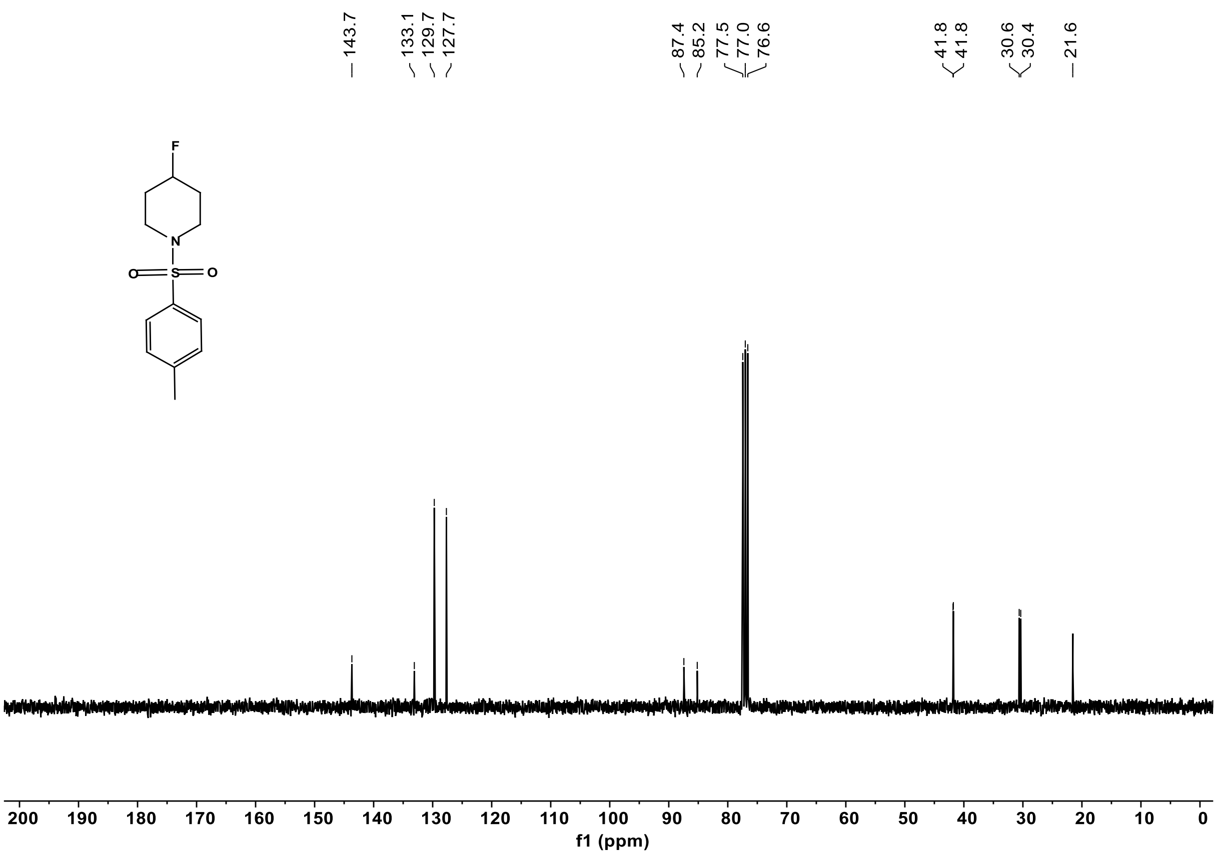

$^{13}C\{^{1}H\}$ NMR spectrum of compound **2J** (75 MHz, $CDCl_3$)

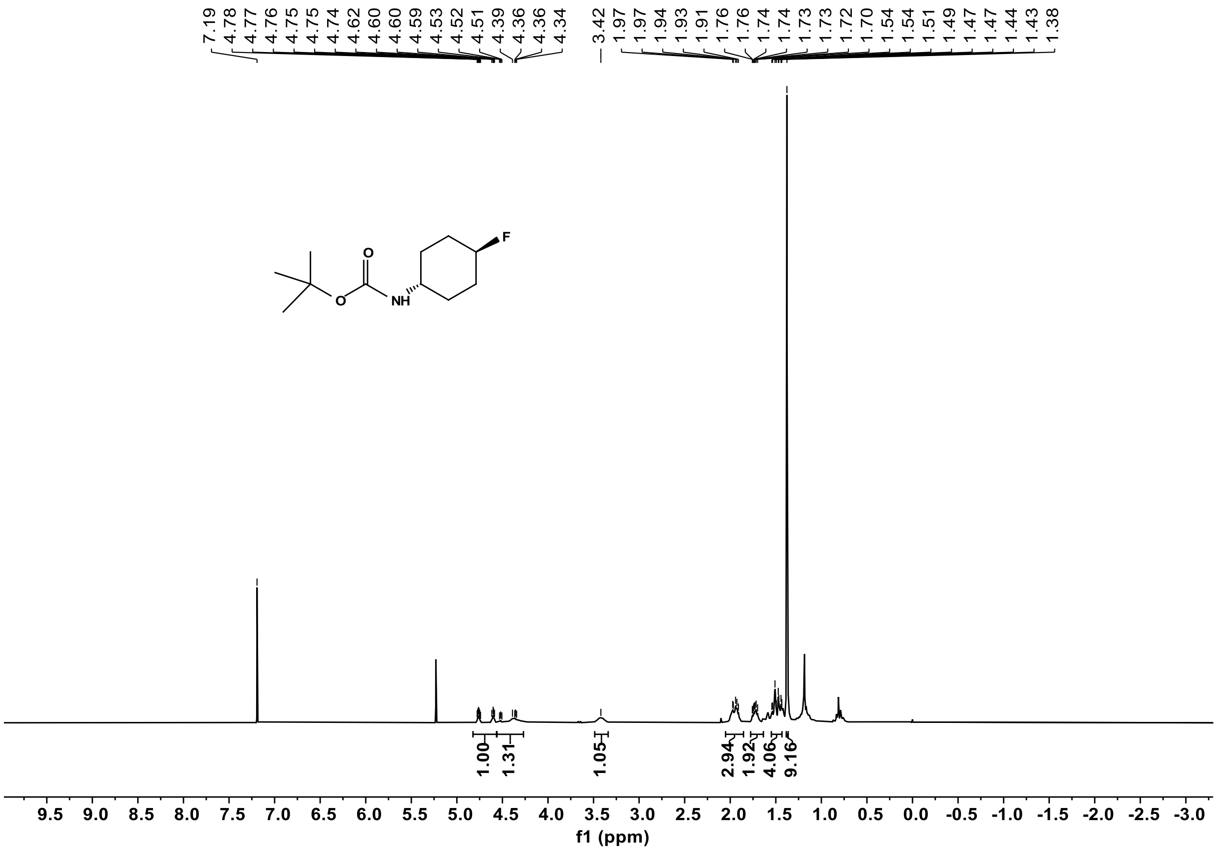

^{1}H NMR spectrum of compound **2k** (300 MHz, $CDCl_3$)

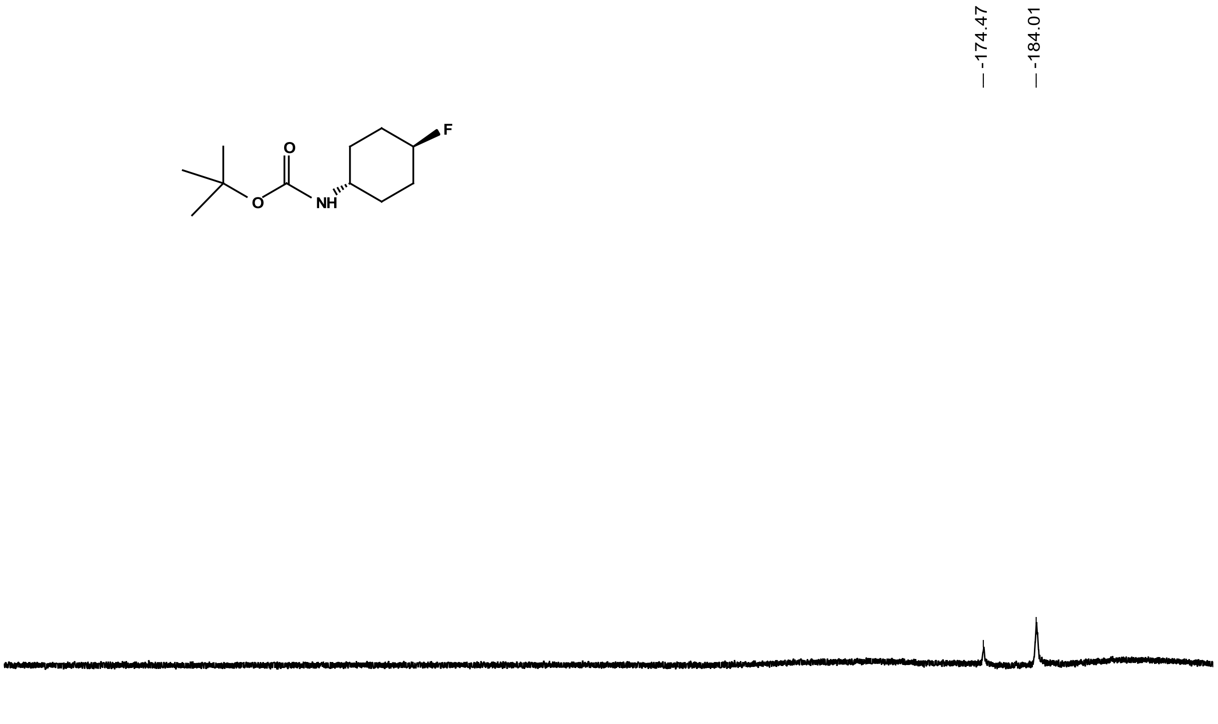

^{19}F NMR spectrum of compound **2k** (282 MHz, $CDCl_3$)

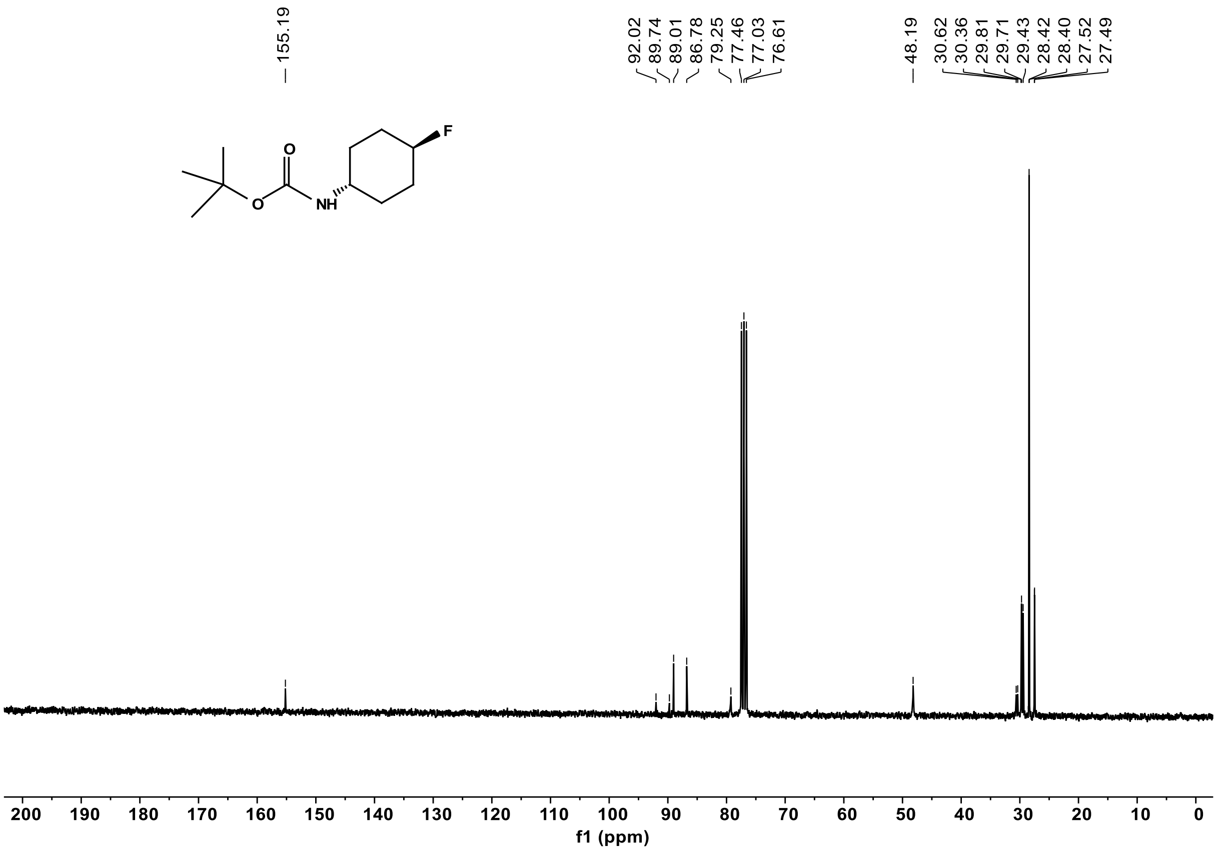

$^{13}C\{^{1}H\}$ NMR spectrum of compound **2k** (75 MHz, $CDCl_3$)

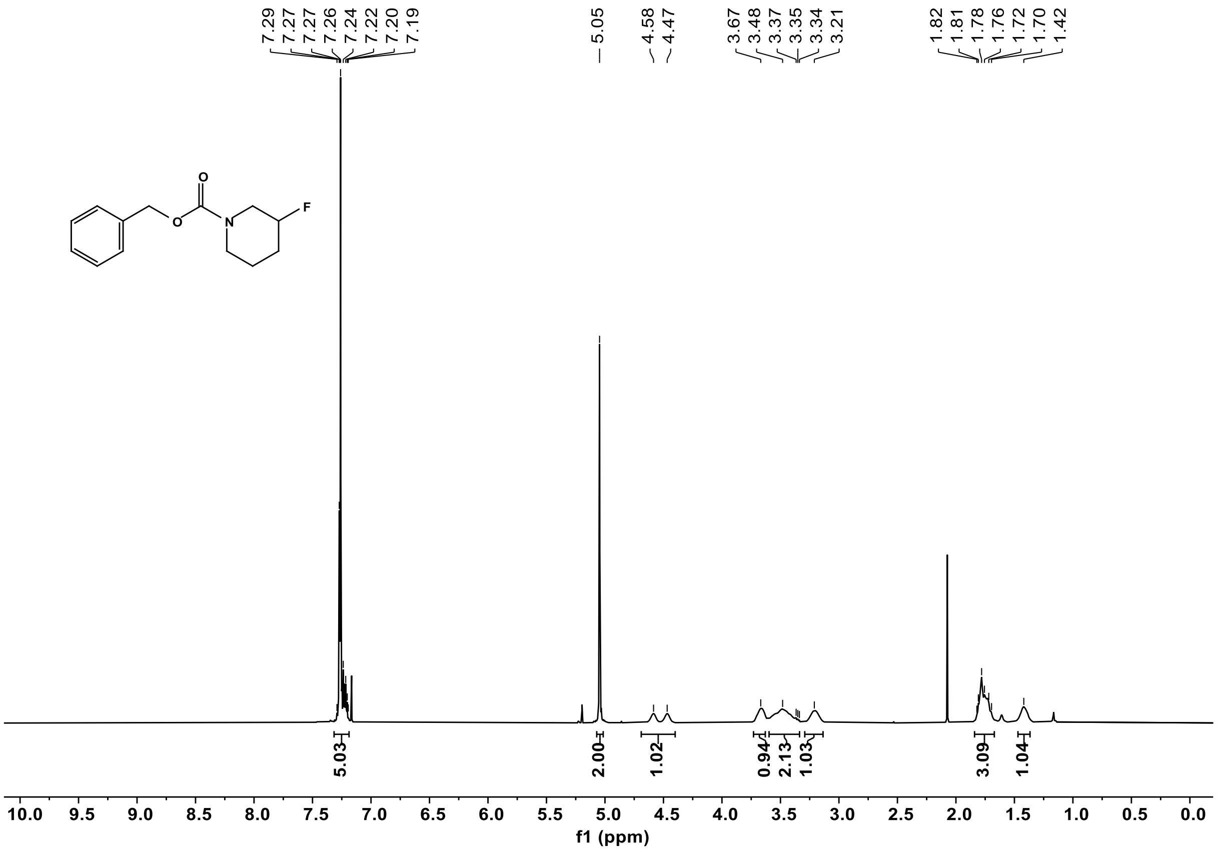

^{1}H NMR spectrum of compound **2l** (400 MHz, $CDCl_3$)

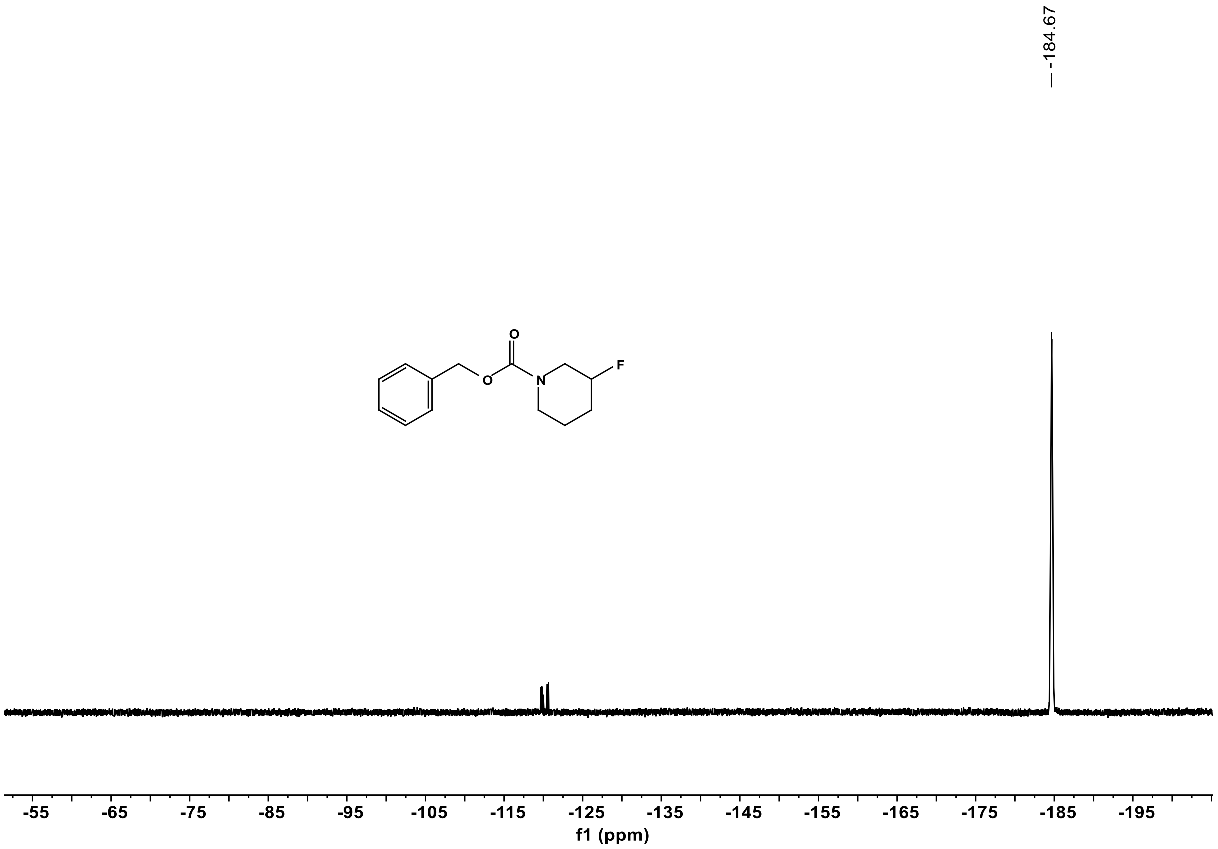

^{19}F NMR spectrum of compound **2l** (376 MHz, $CDCl_3$)

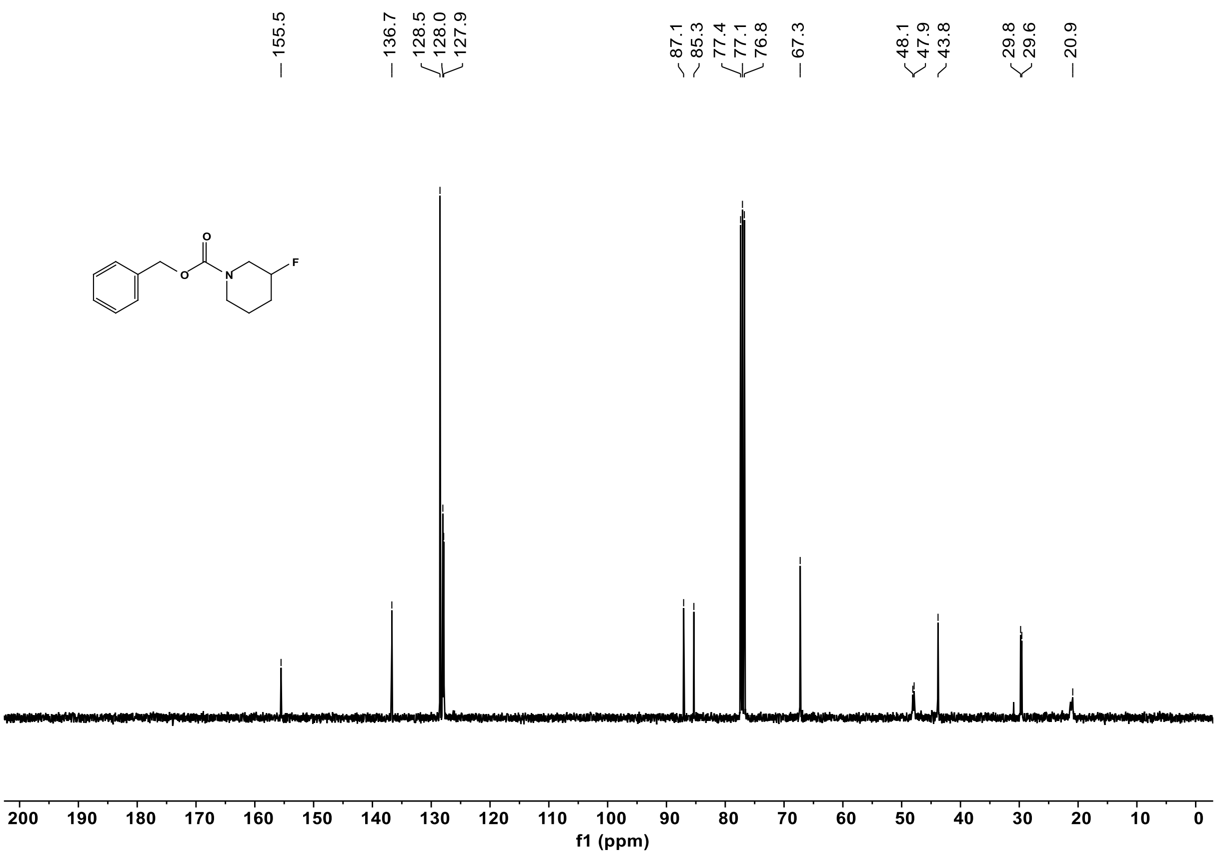

$^{13}C\{^1H\}$ NMR spectrum of compound **2l** (101 MHz, $CDCl_3$)

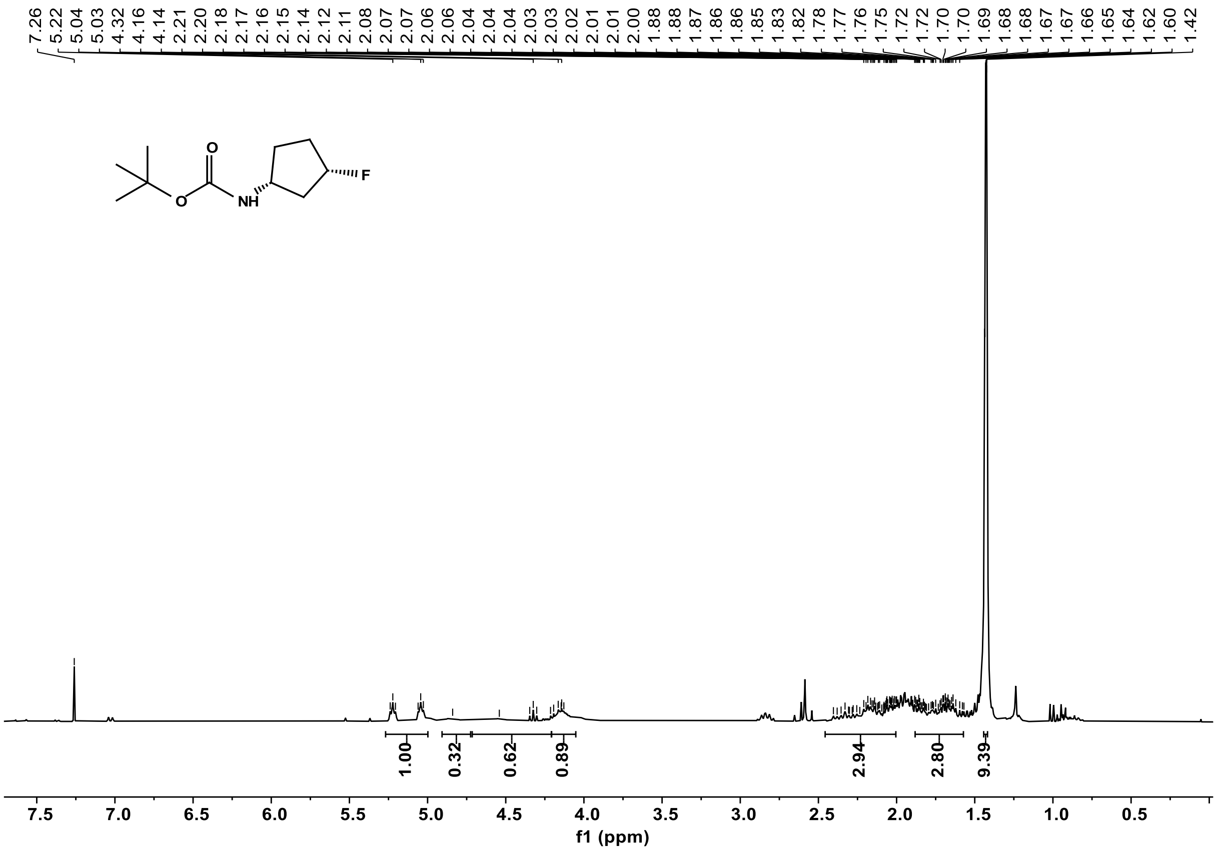

^{1}H NMR spectrum of compound **2m** (300 MHz, $CDCl_3$)

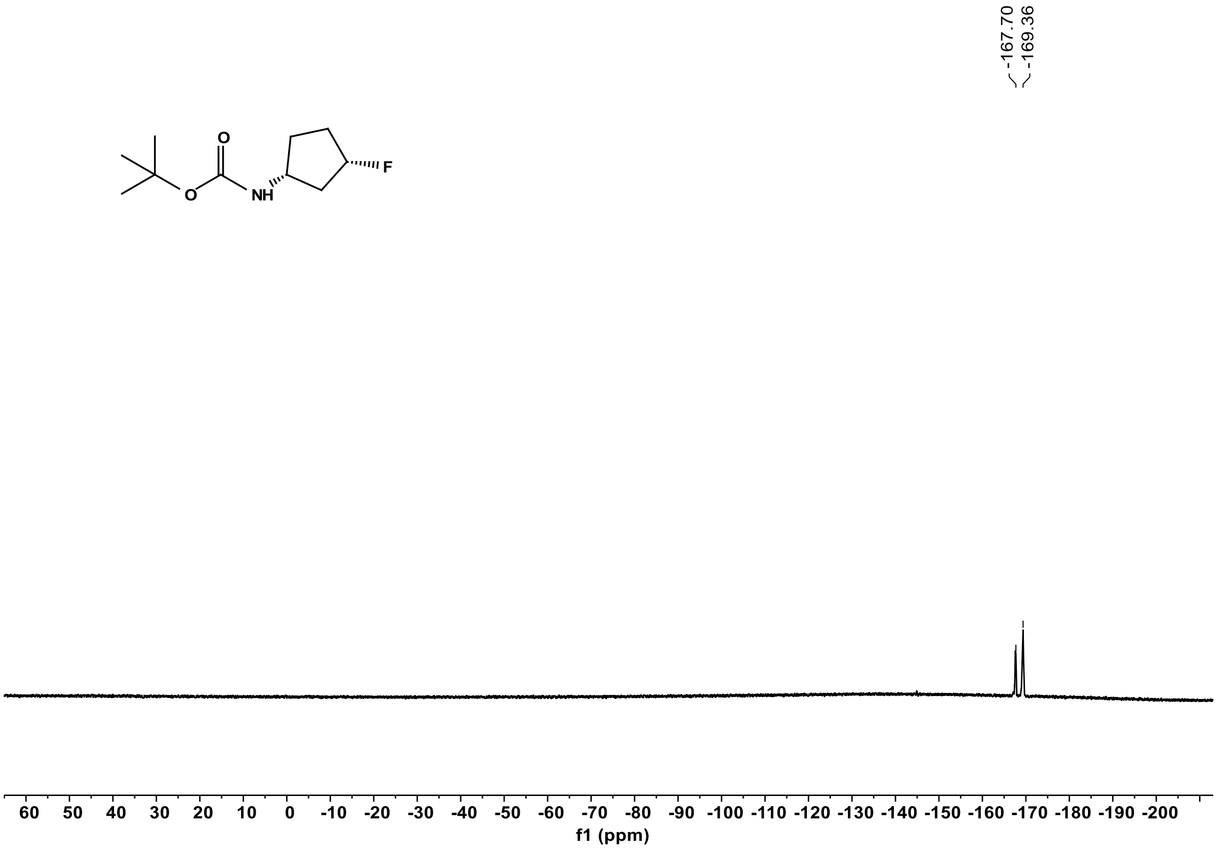

^{19}F NMR spectrum of compound **2m** (376 MHz, $CDCl_3$)

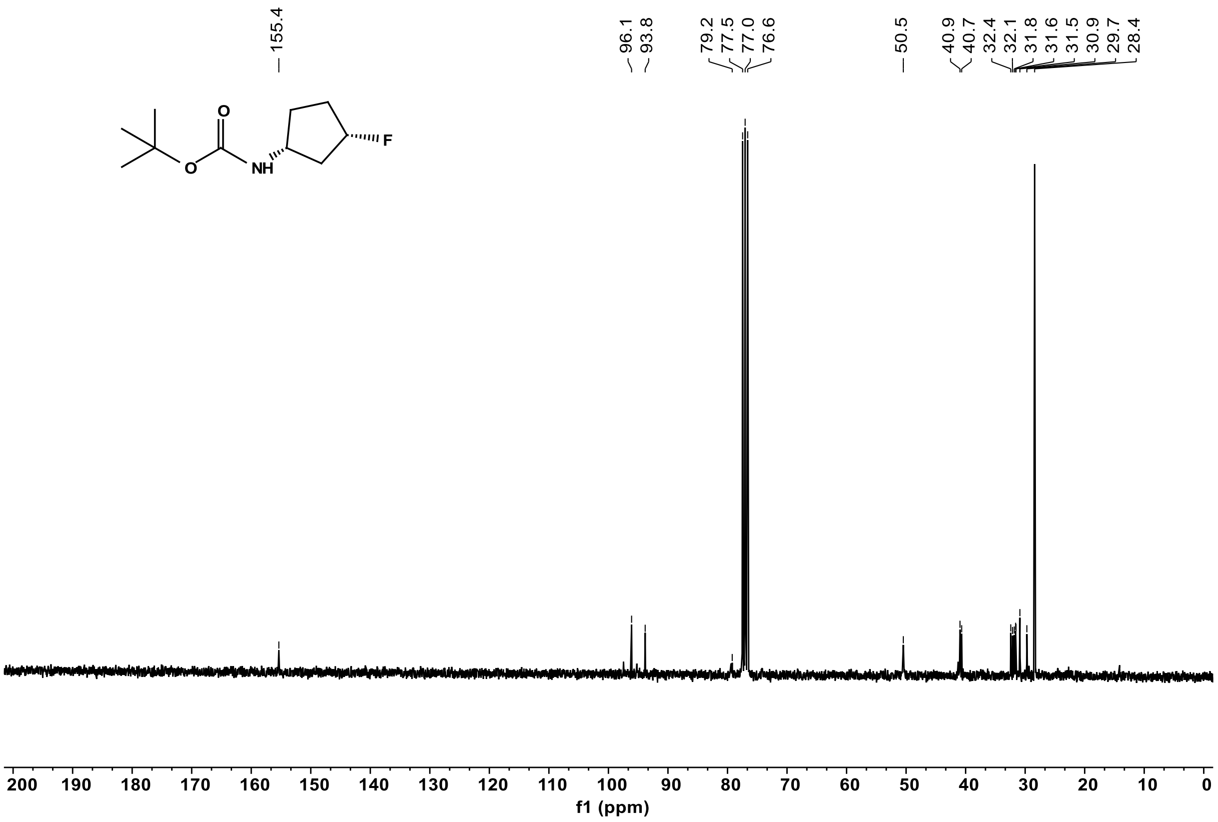

$^{13}C\{^{1}H\}$ NMR spectrum of compound **2m** (75 MHz, $CDCl_3$)

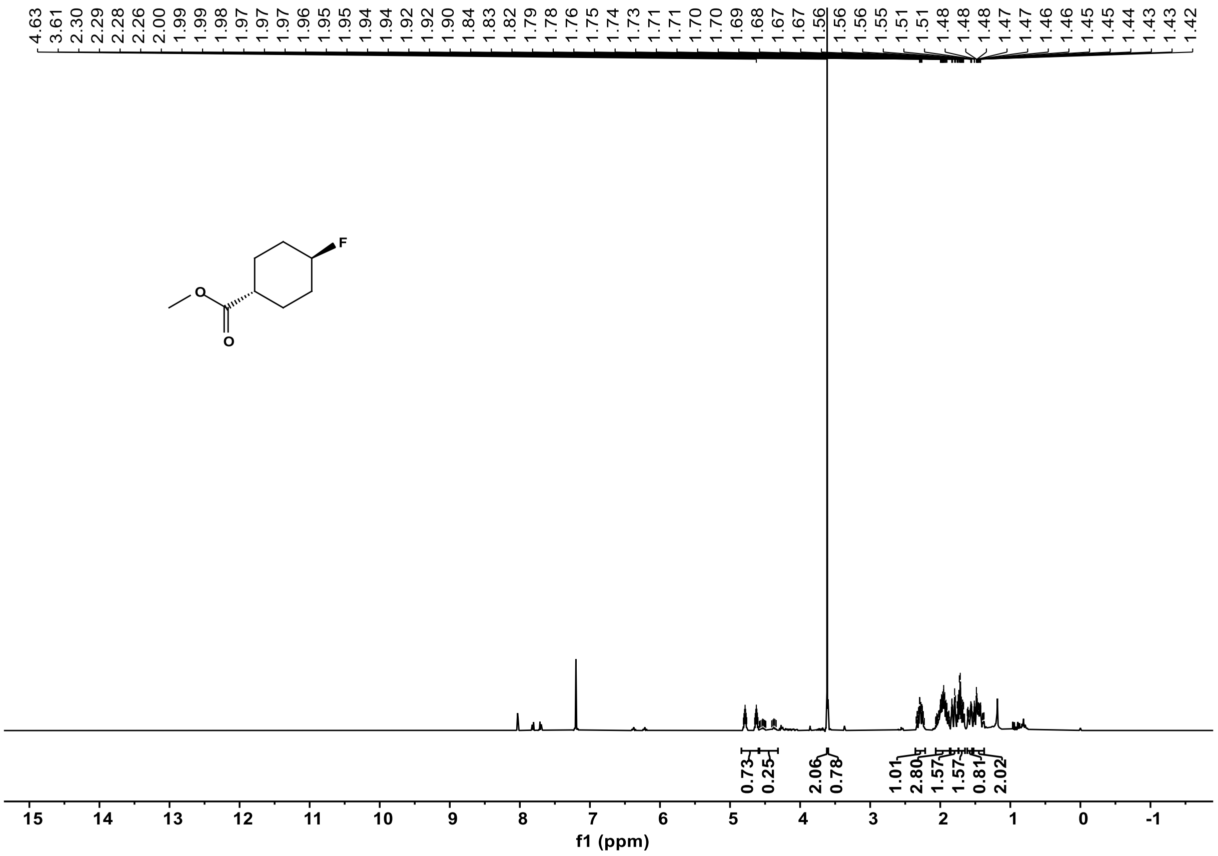

^{1}H NMR spectrum of compound **2n** (400 MHz, $CDCl_3$)

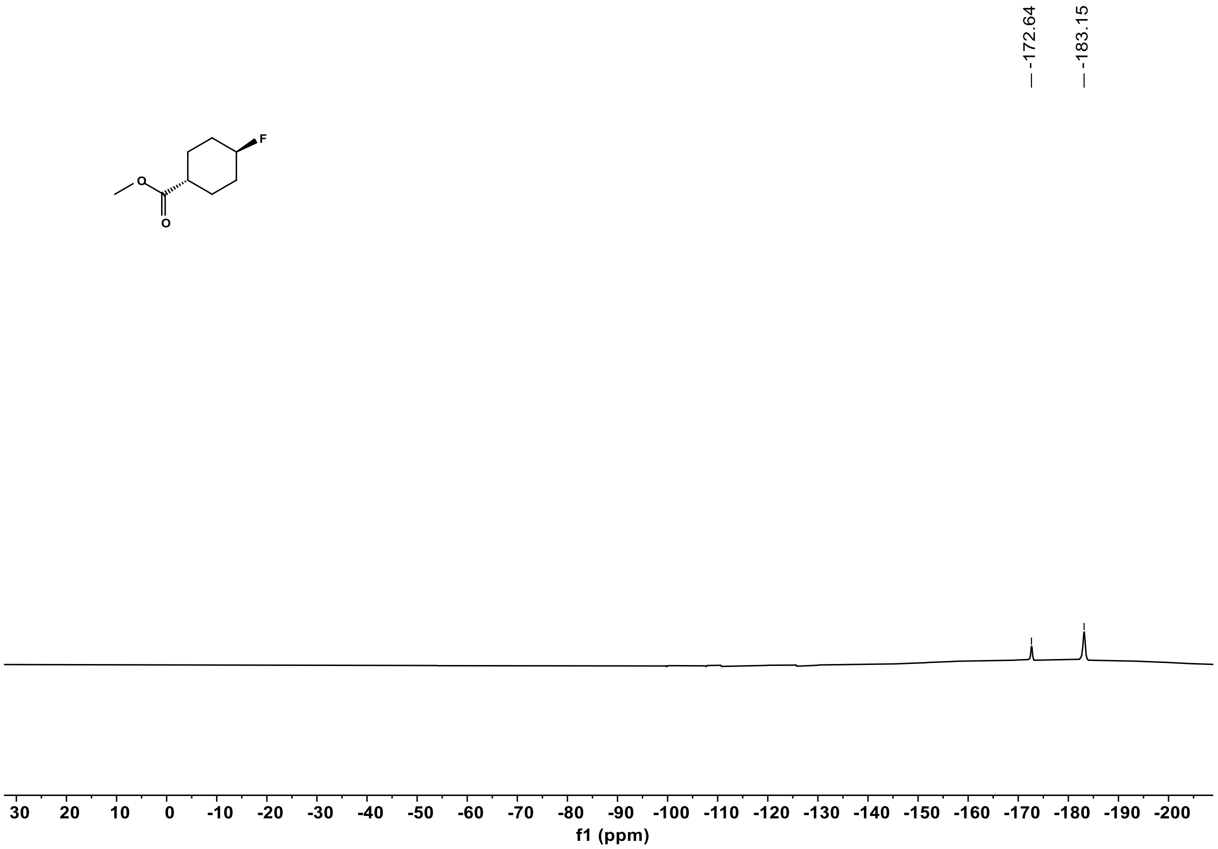

^{19}F NMR spectrum of compound **2n** (282 MHz, $CDCl_3$)

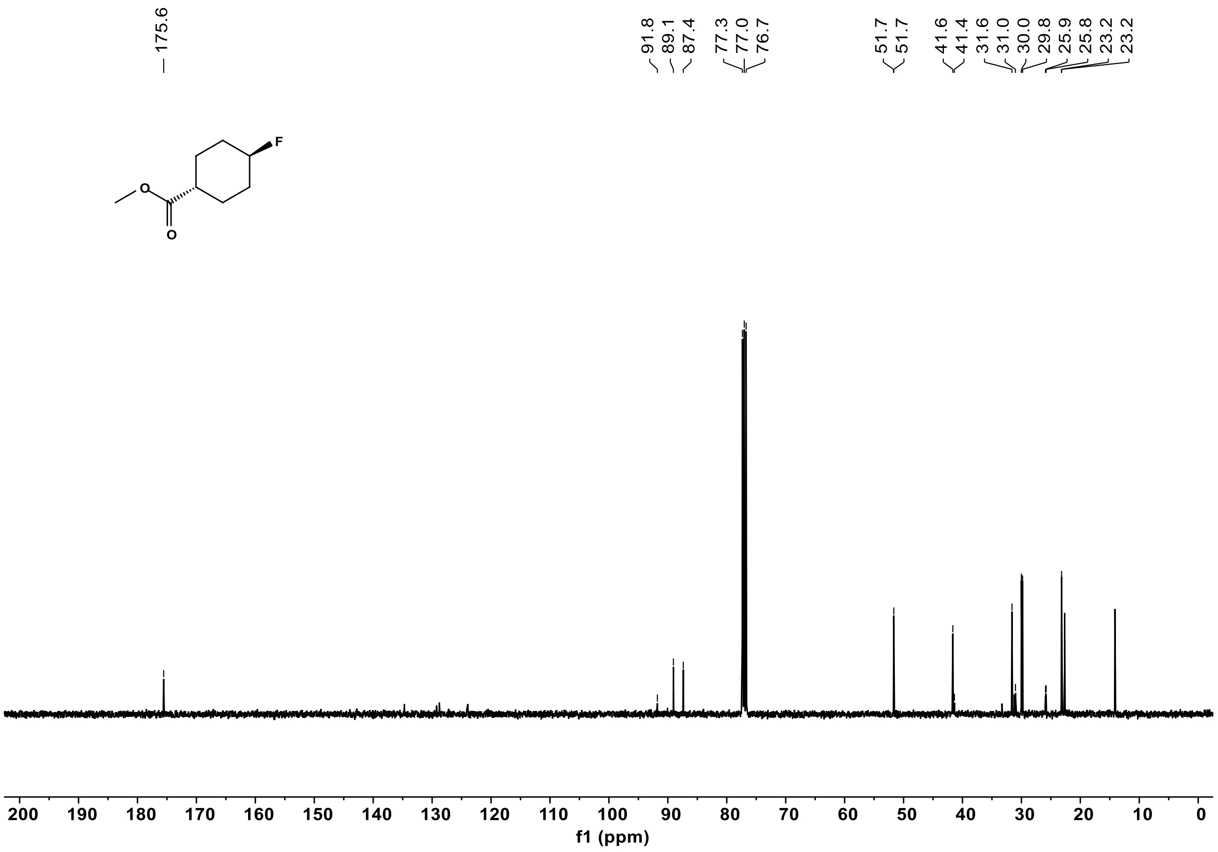

$^{13}C\{^1H\}$ NMR spectrum of compound **2n** (101 MHz, $CDCl_3$)

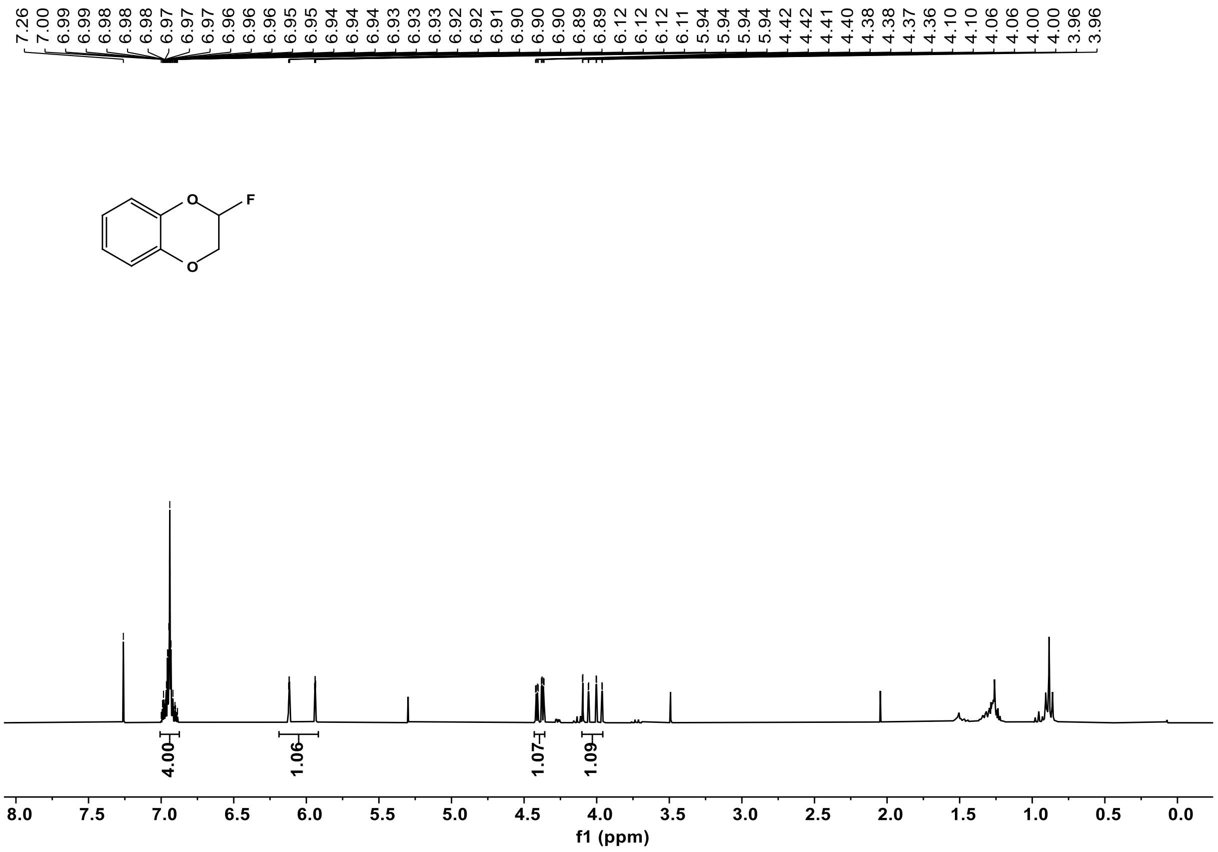

^{1}H NMR spectrum of compound **2o** (300 MHz, $CDCl_3$)

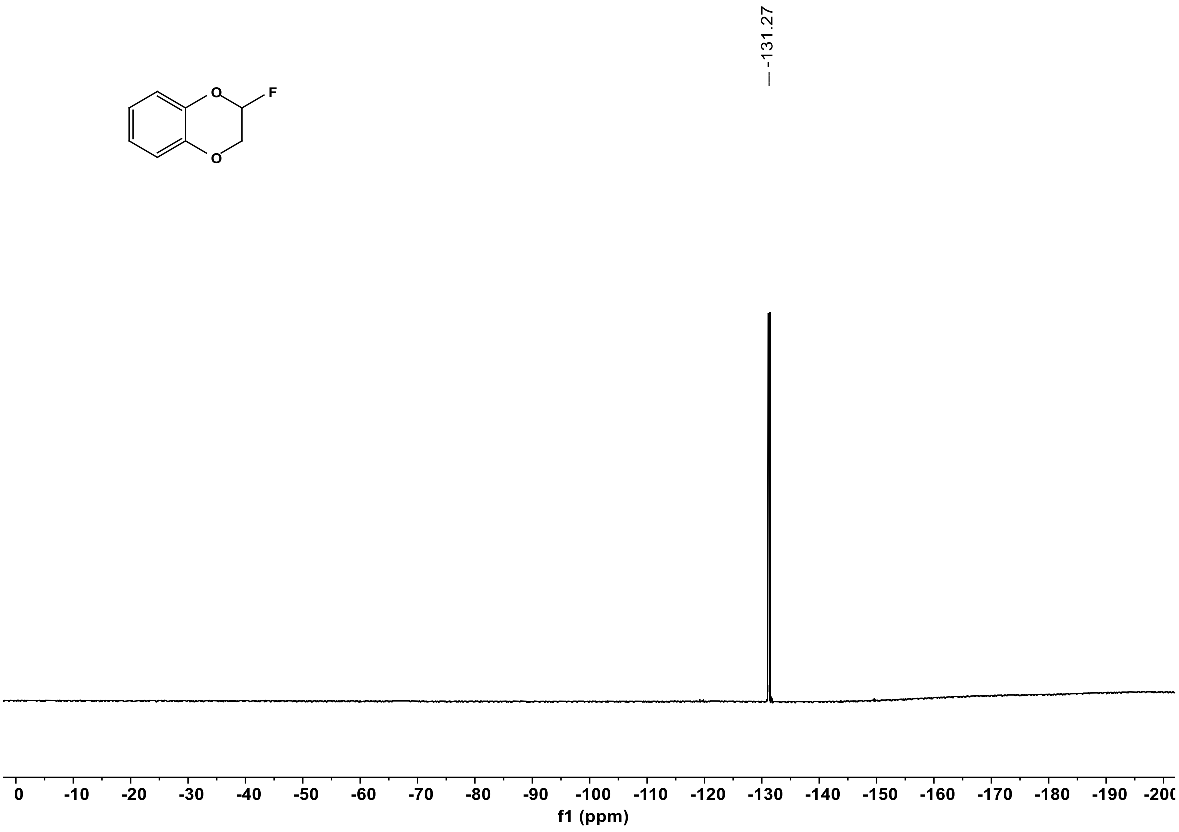

^{19}F NMR spectrum of compound **2o** (282 MHz, $CDCl_3$)

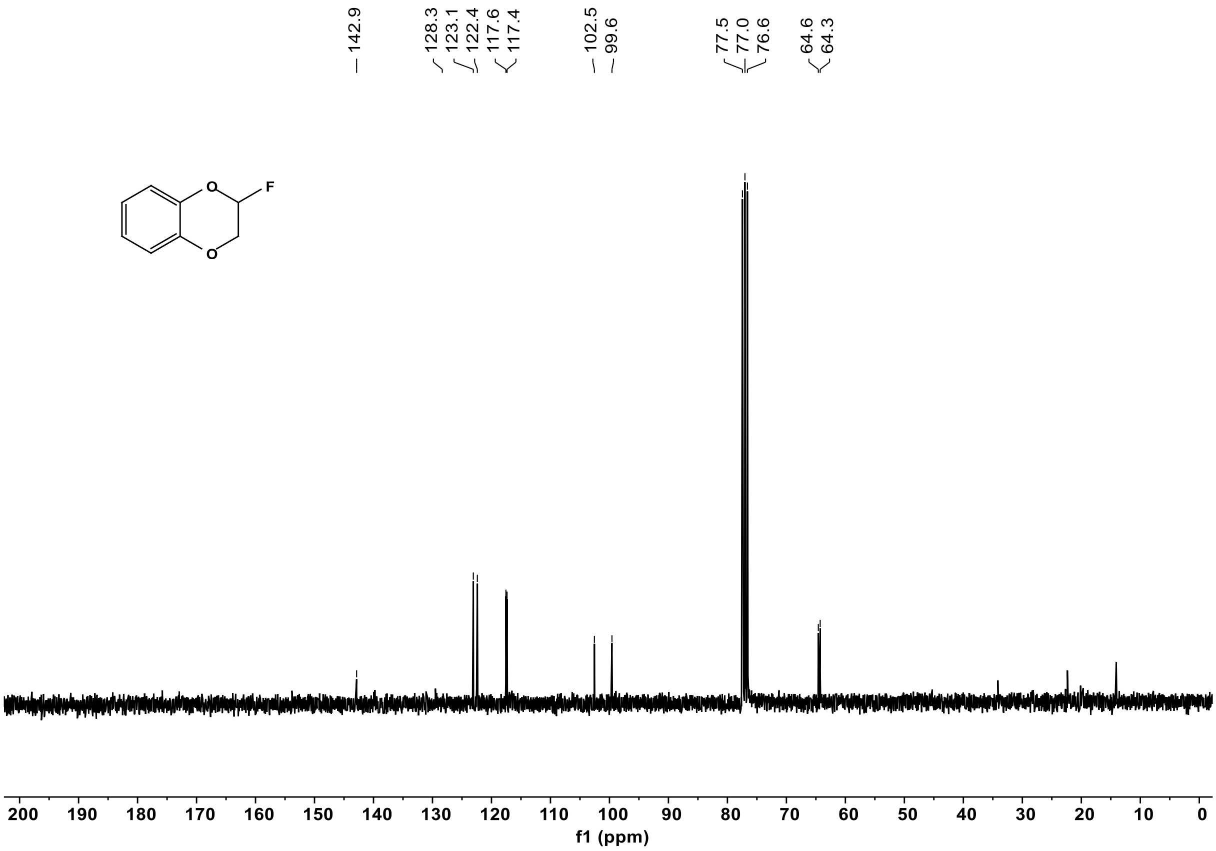

$^{13}C\{^{1}H\}$ NMR spectrum of compound **2o** (75 MHz, $CDCl_3$)

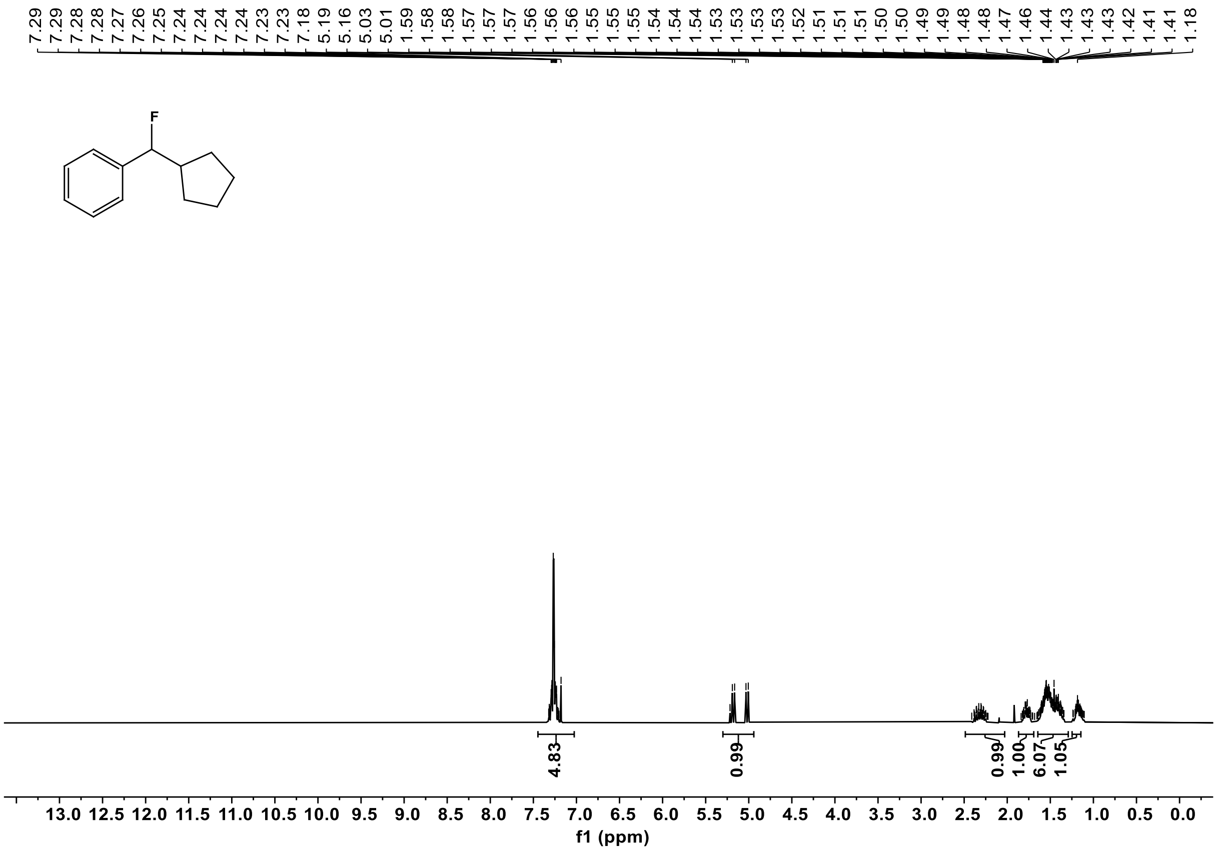

^{1}H NMR spectrum of compound **2p** (300 MHz, $CDCl_3$)

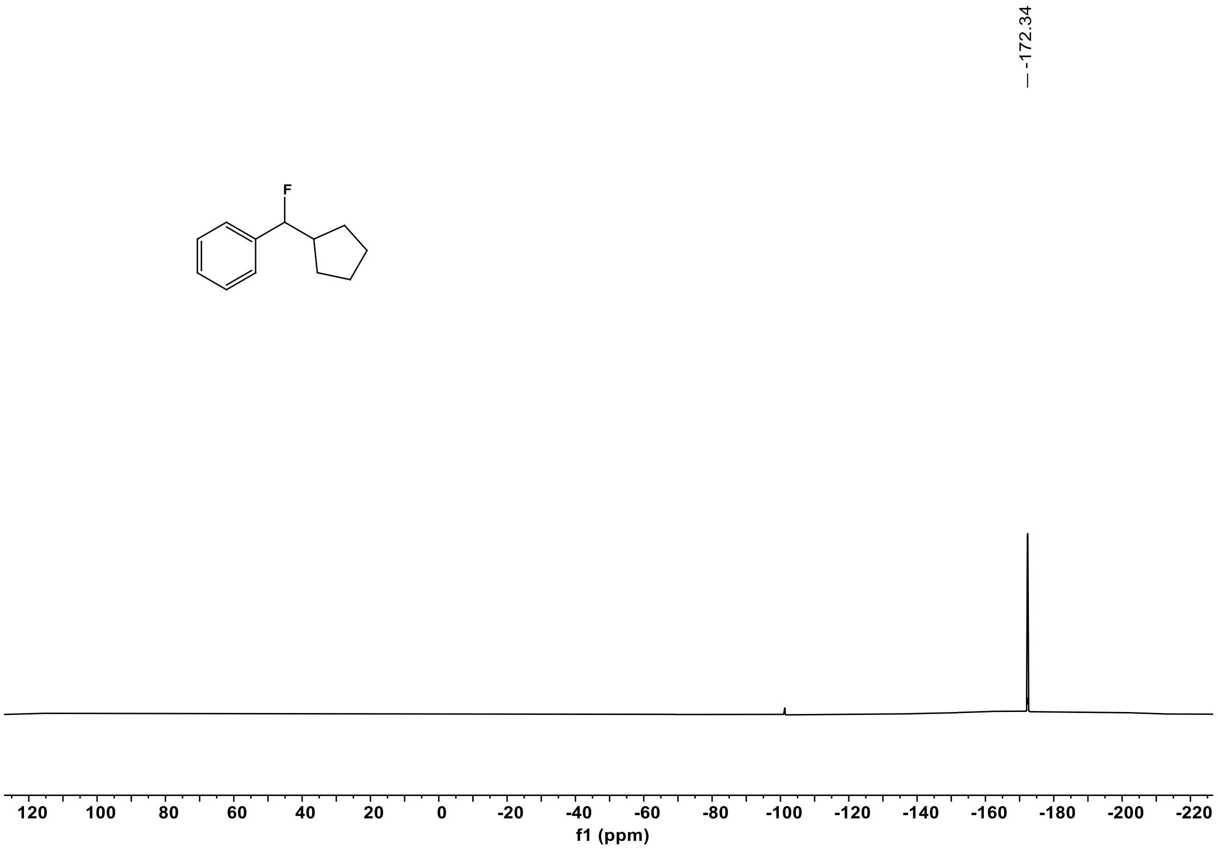

^{19}F NMR spectrum of compound **2p** (282 MHz, $CDCl_3$)

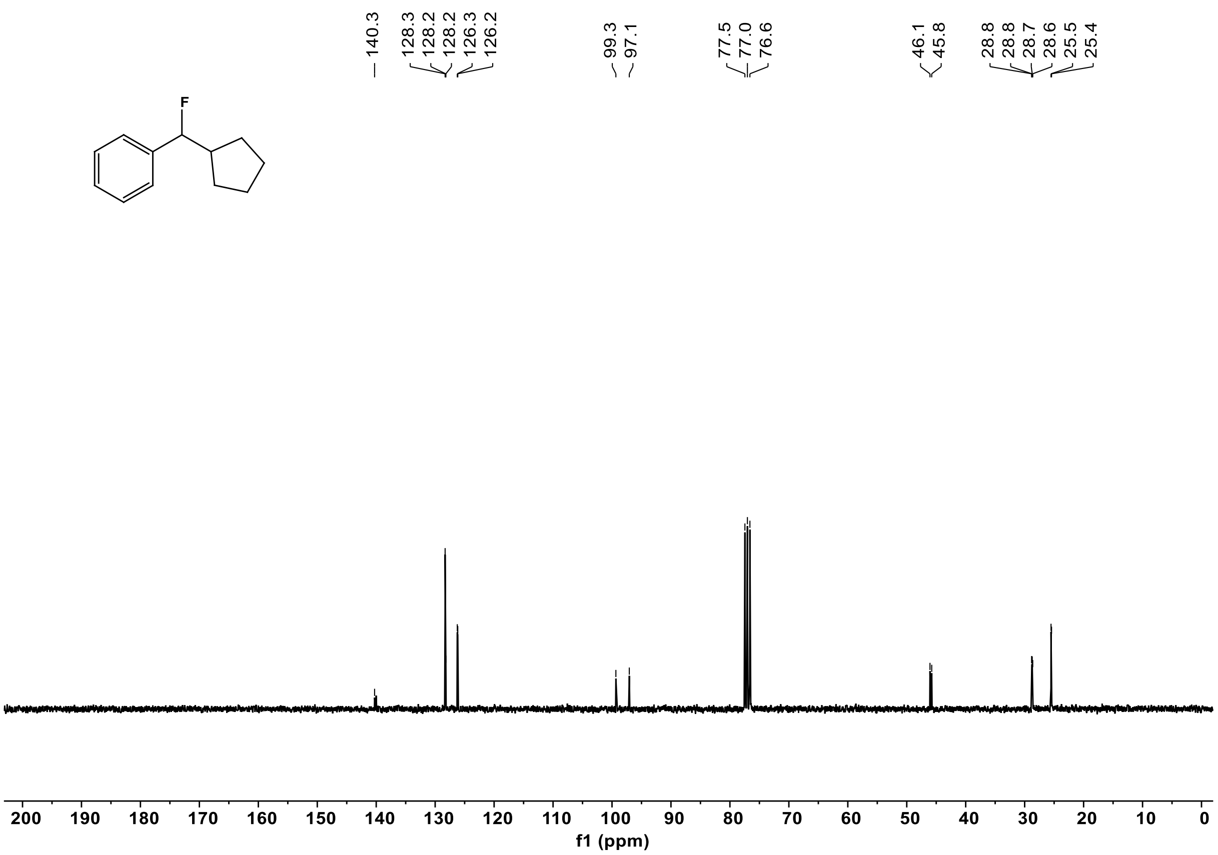

$^{13}C\{^1H\}$ NMR spectrum of compound **2p** (75 MHz, $CDCl_3$)

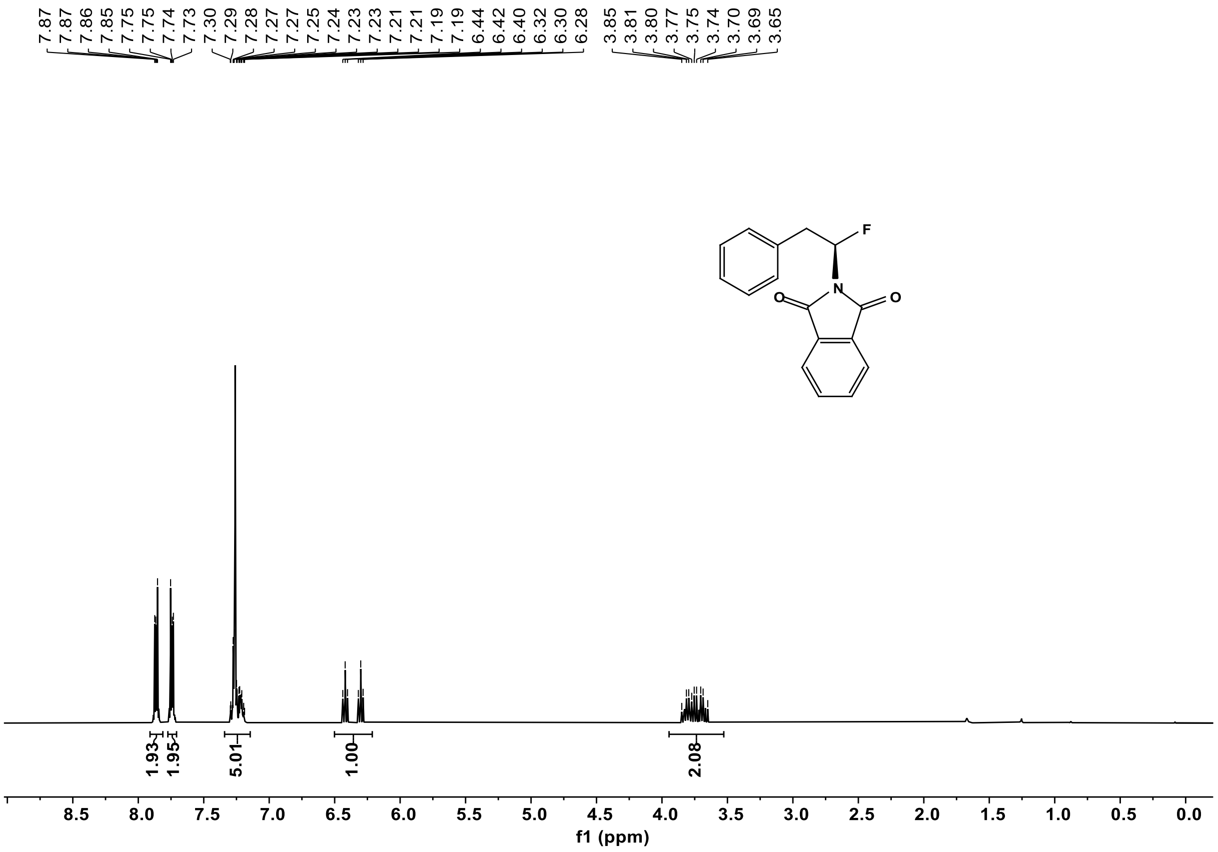

^{1}H NMR spectrum of compound **2q** (300 MHz, $CDCl_3$)

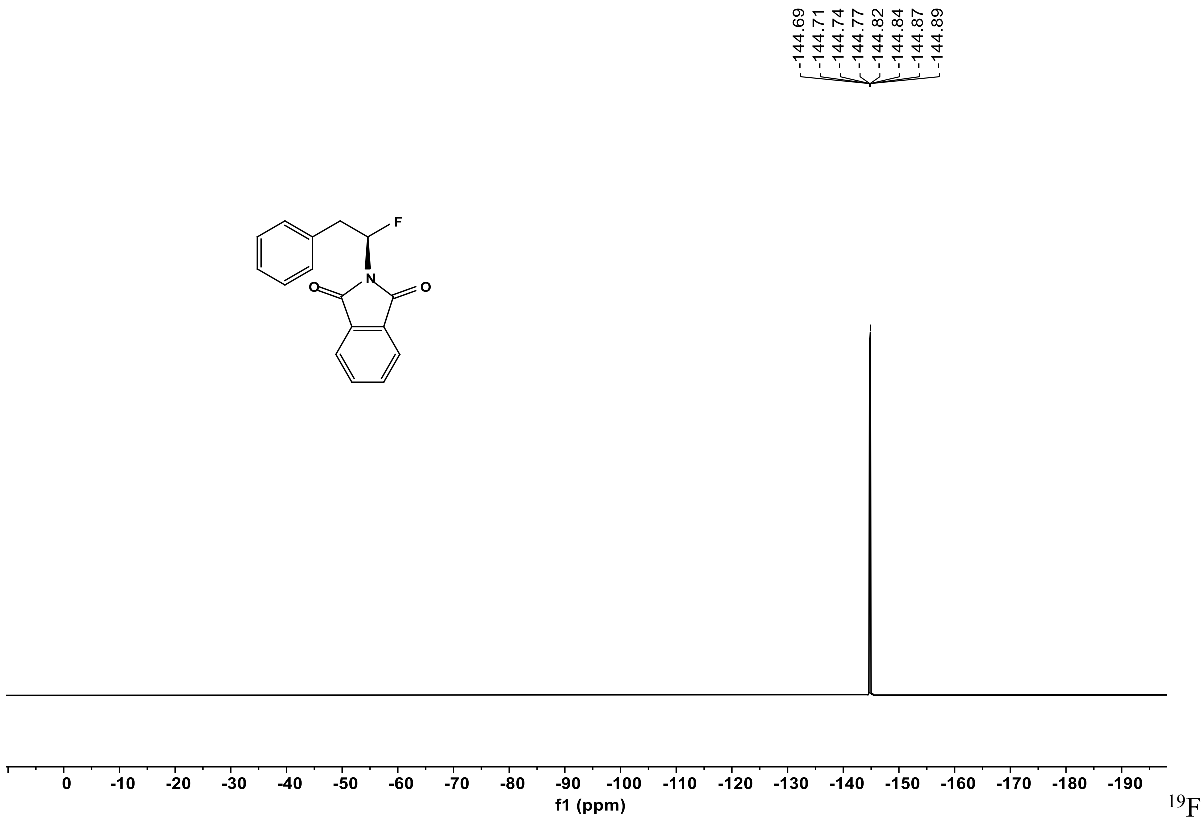

^{19}F NMR spectrum of compound **2q** (376 MHz, $CDCl_3$)

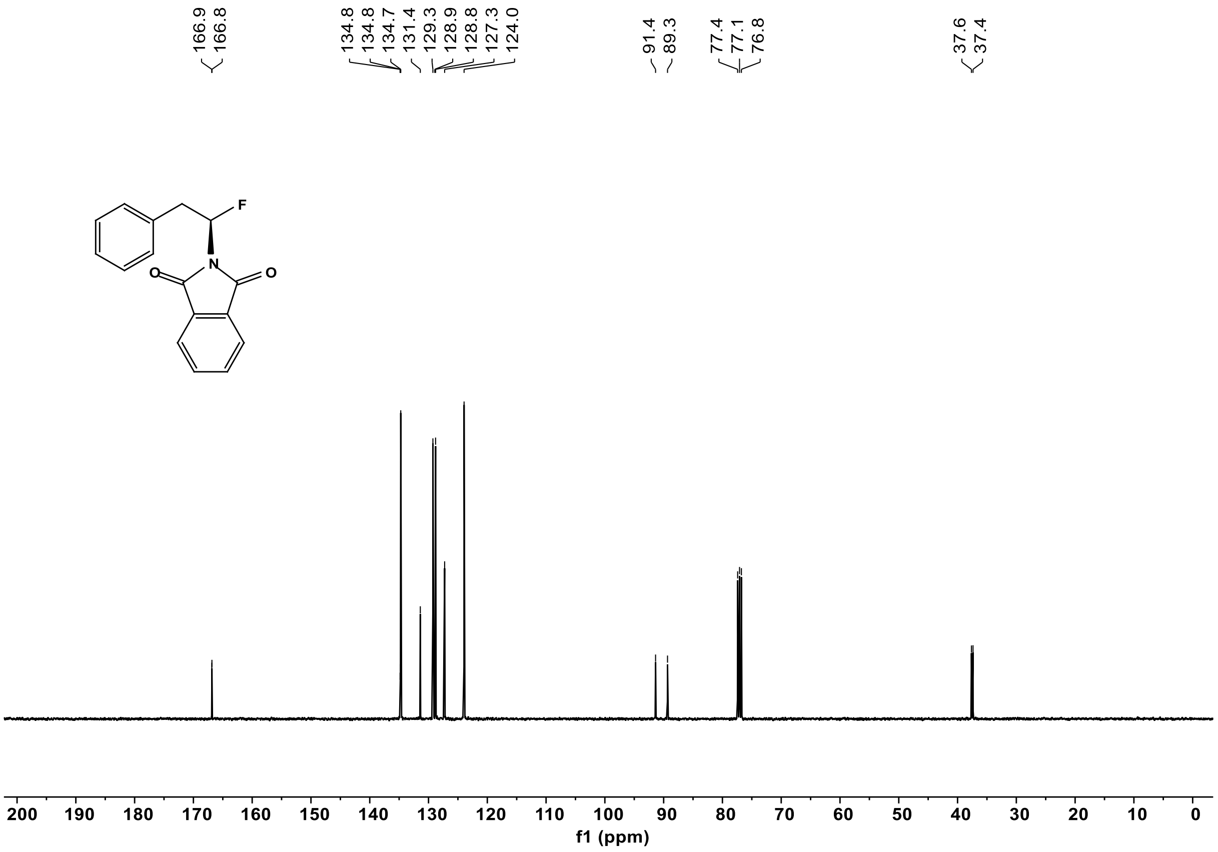

$^{13}C\{^1H\}$ NMR spectrum of compound **2q** (75 MHz, $CDCl_3$)

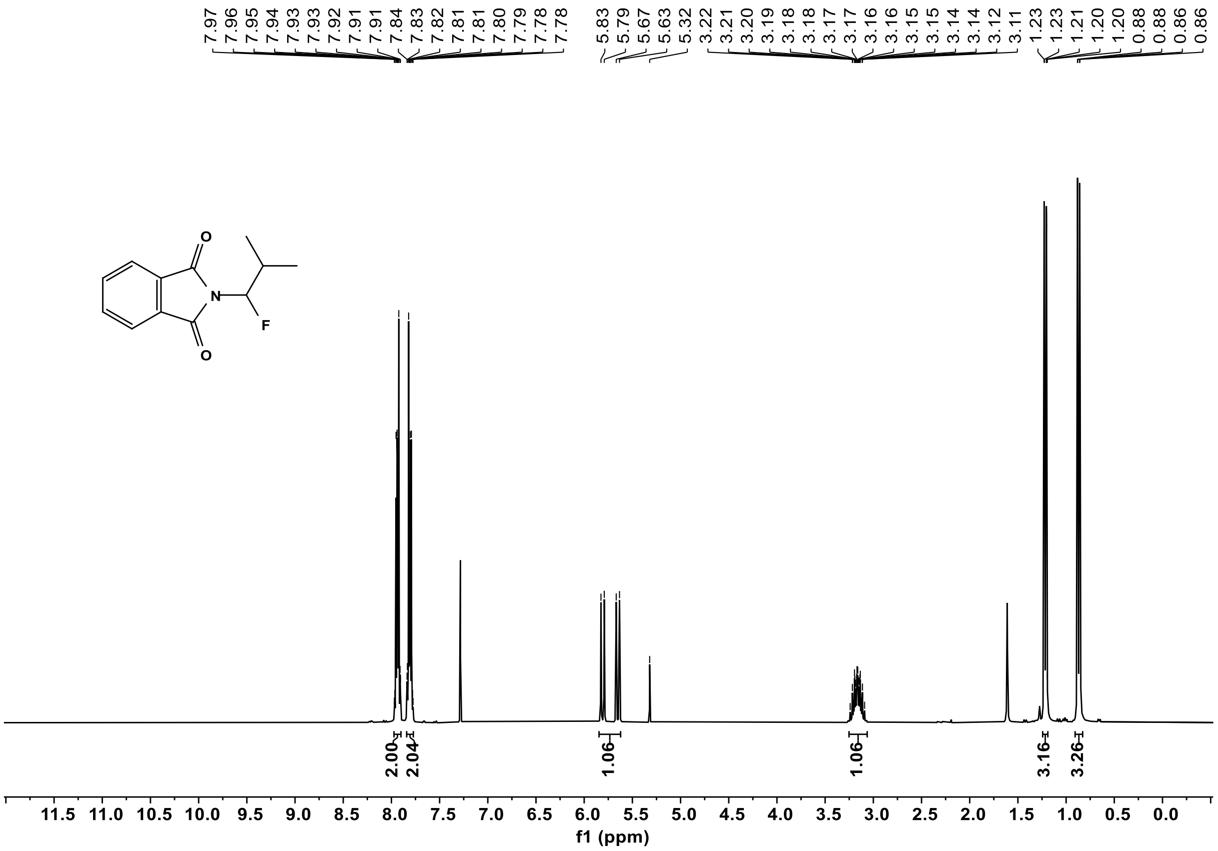

^{1}H NMR spectrum of compound **2r** (300 MHz, $CDCl_3$)

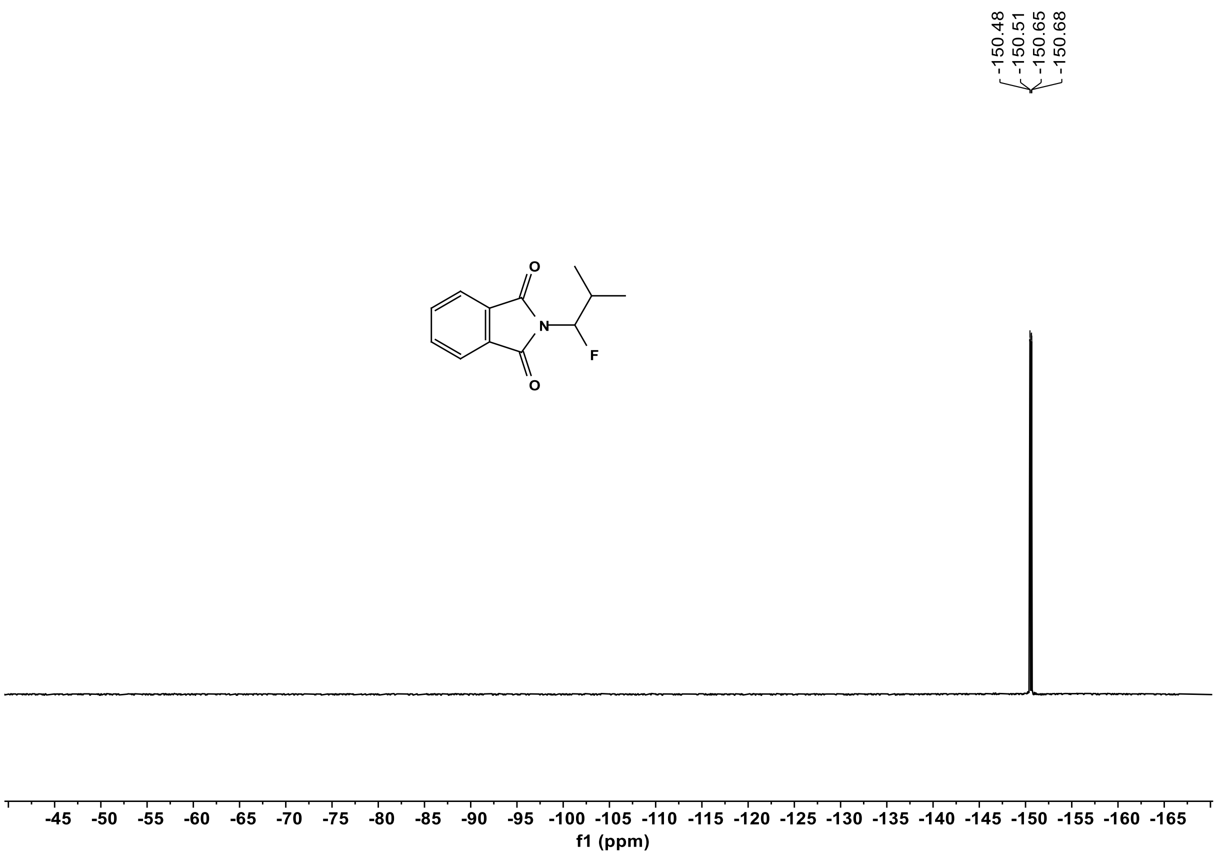

^{19}F NMR spectrum of compound **2r** (282 MHz, $CDCl_3$)

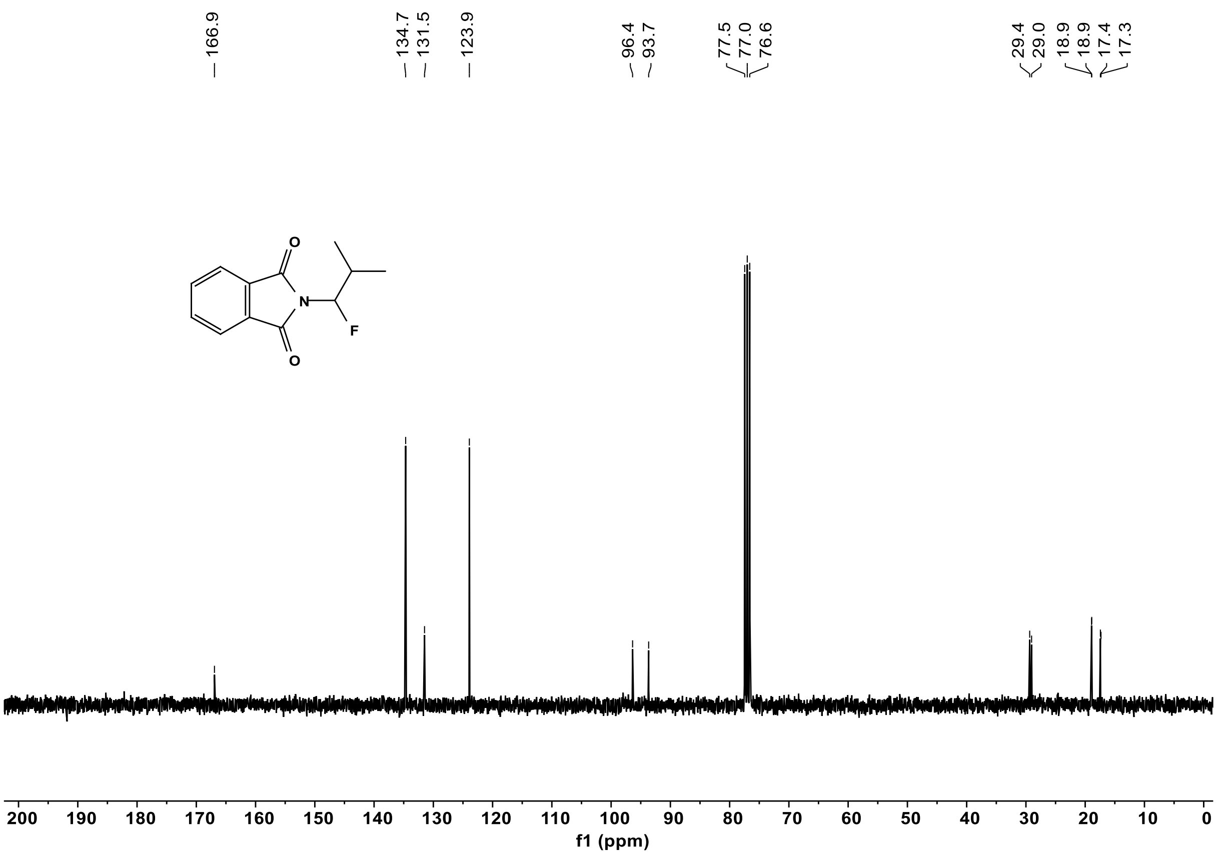

$^{13}C\{^{1}H\}$ NMR spectrum of compound **2r** (75 MHz, $CDCl_3$)

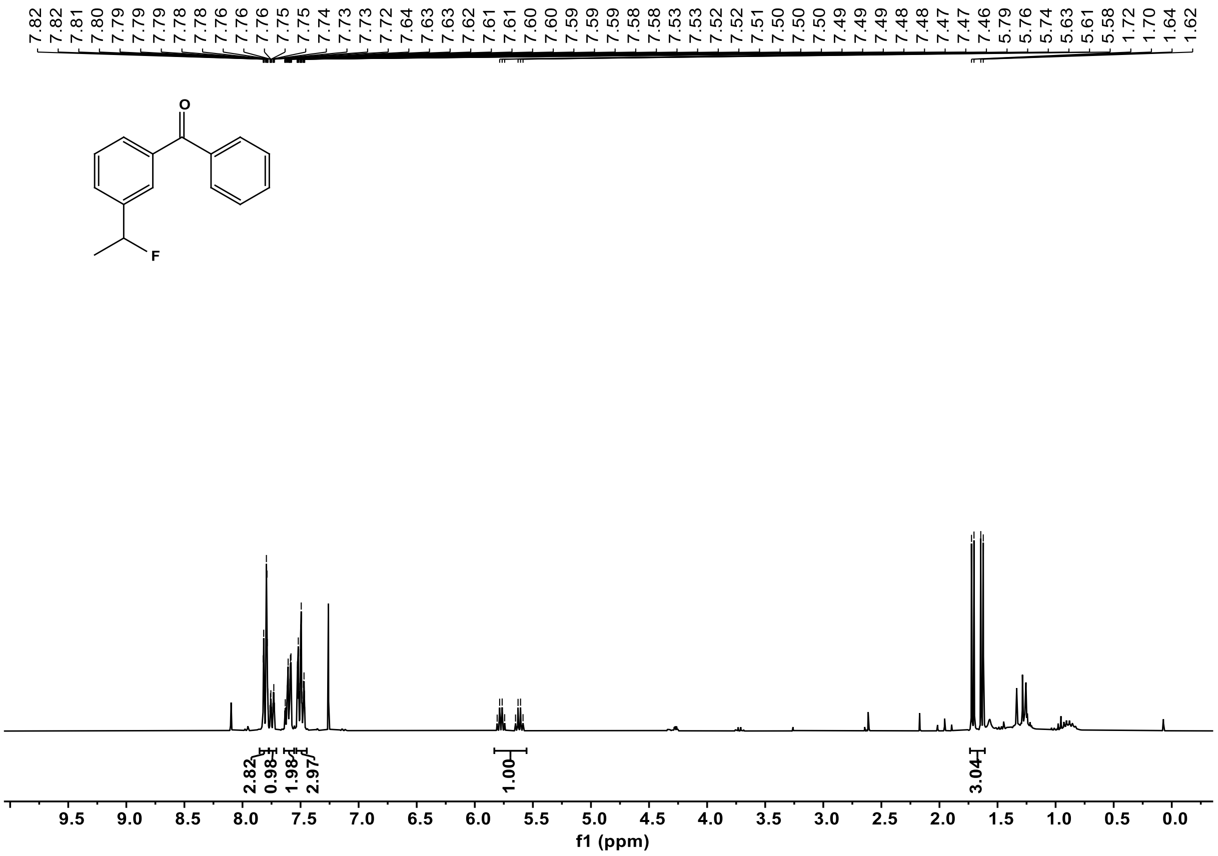

^{1}H NMR spectrum of compound **2s** (300 MHz, $CDCl_3$)

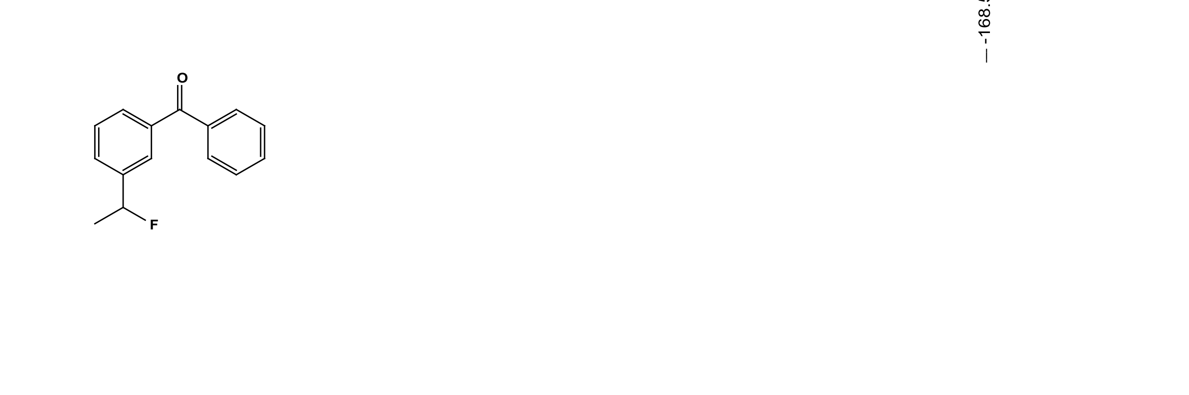

^{19}F NMR spectrum of compound **2s** (282 MHz, $CDCl_3$)

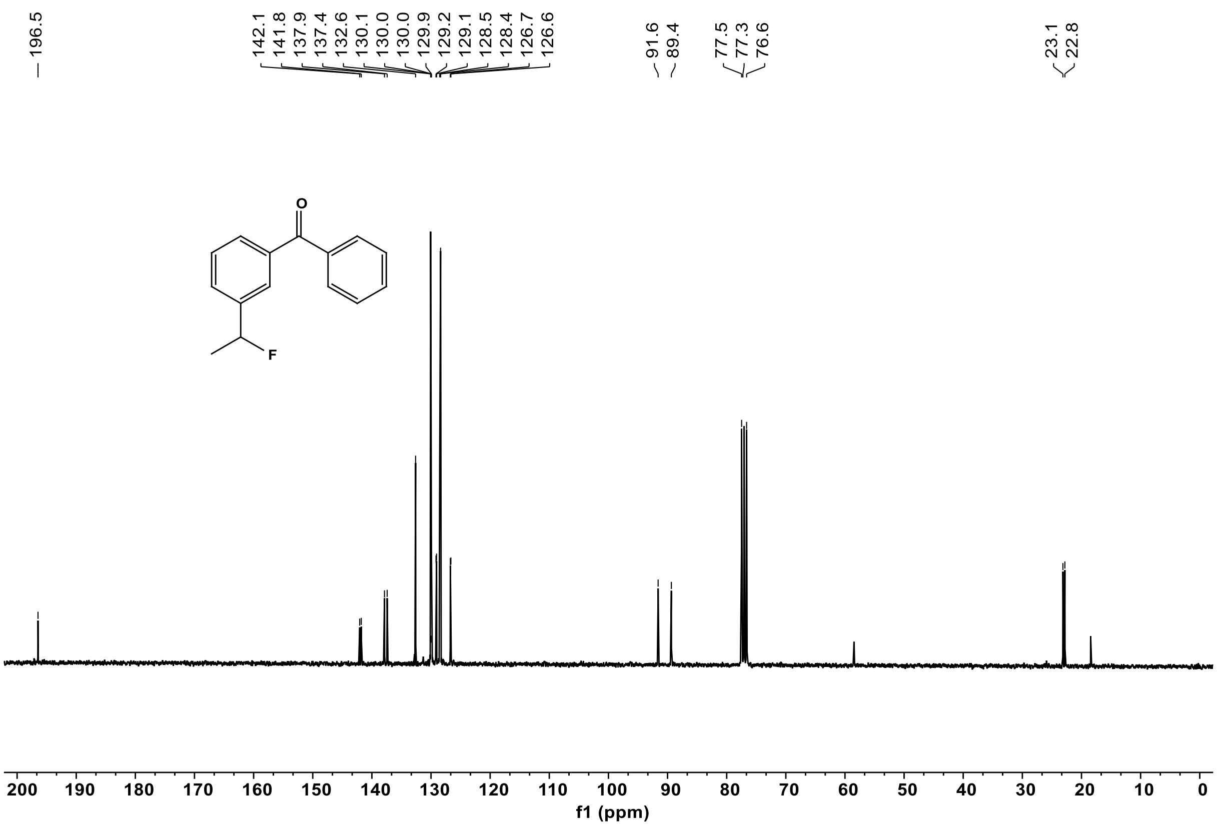

$^{13}C\{^{1}H\}$ NMR spectrum of compound **2s** (75 MHz, $CDCl_3$)

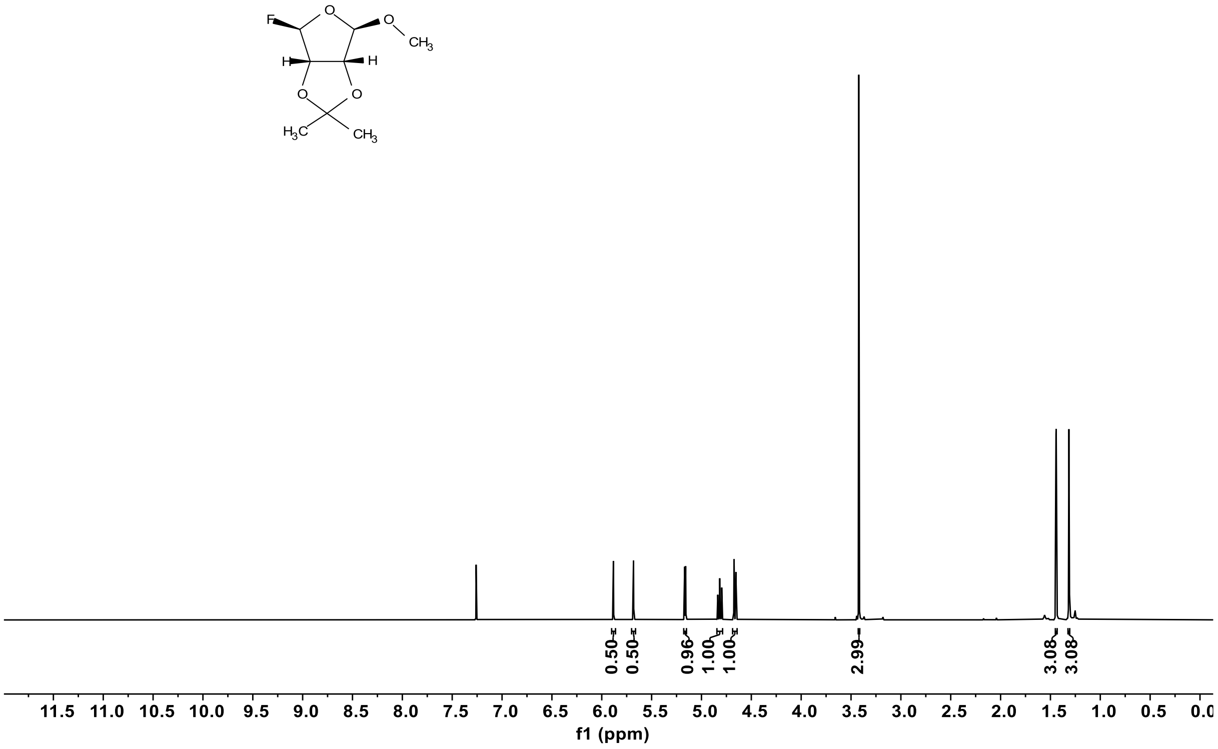

^{1}H NMR spectrum of compound **2t** (300 MHz, $CDCl_3$)

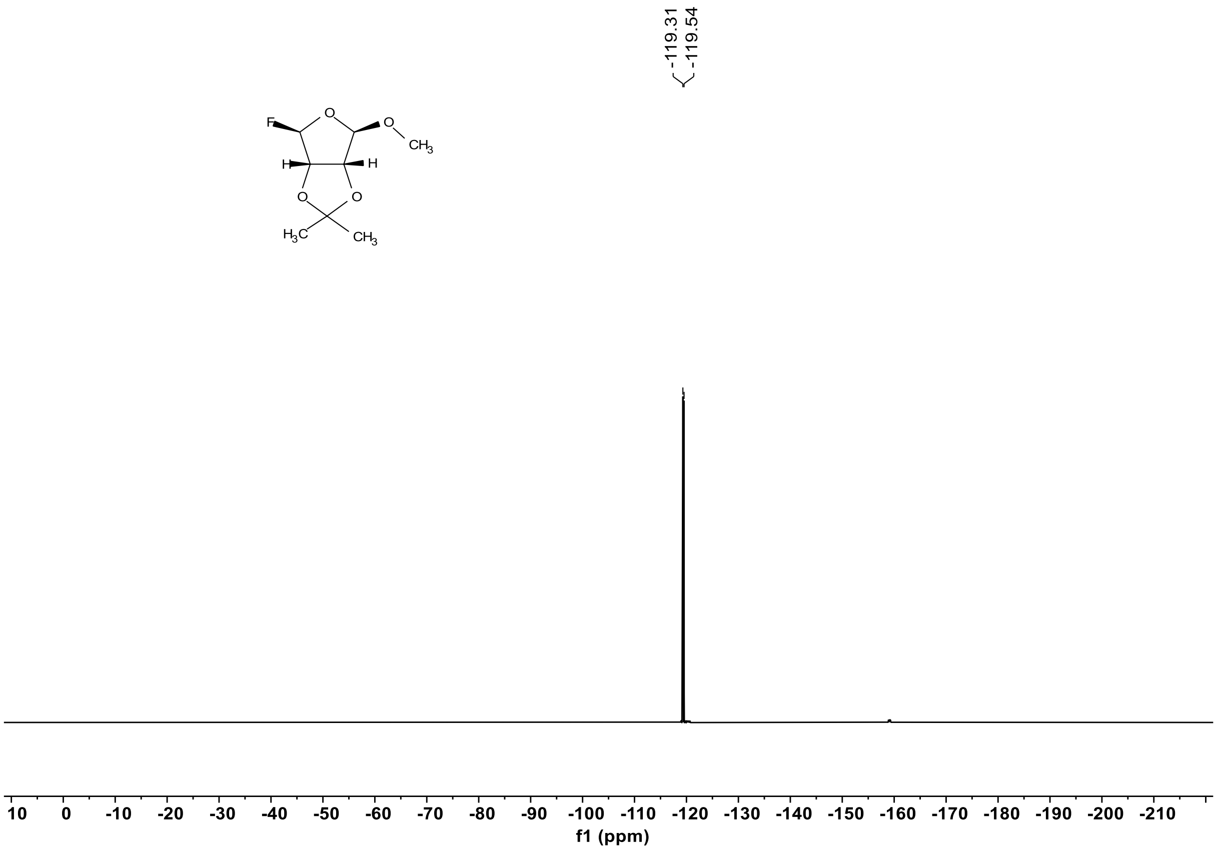

^{19}F NMR spectrum of compound **2t** (282 MHz, $CDCl_3$)

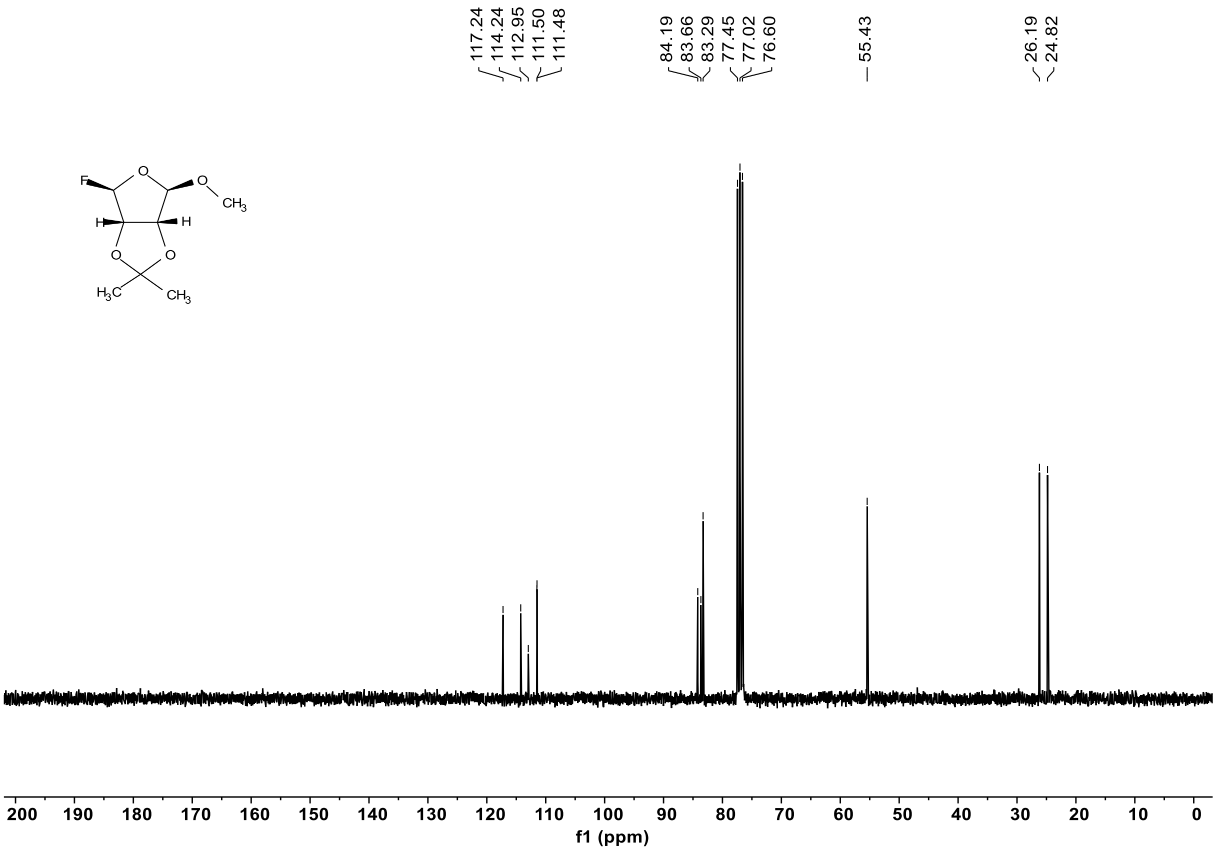

$^{13}C\{^1H\}$ NMR spectrum of compound **2t** (75 MHz, $CDCl_3$)

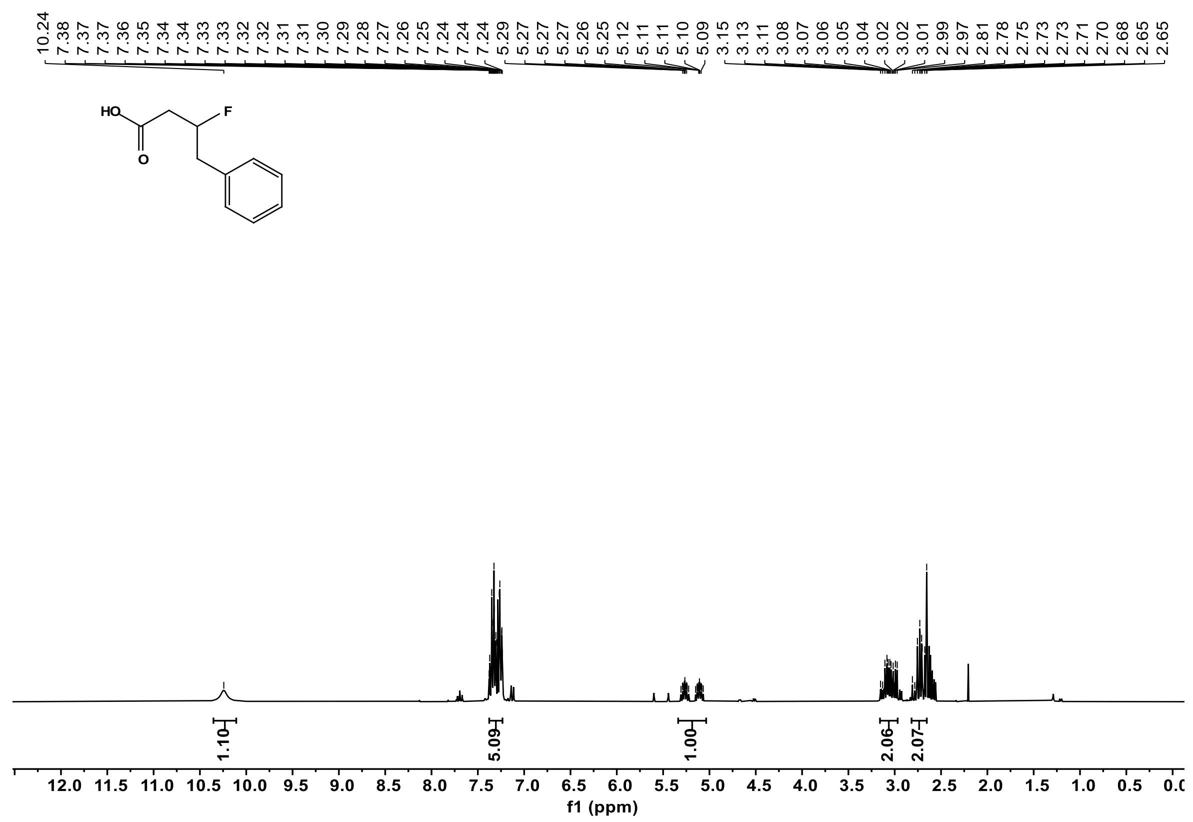

^{1}H NMR spectrum of compound **2u** (300 MHz, $CDCl_3$)

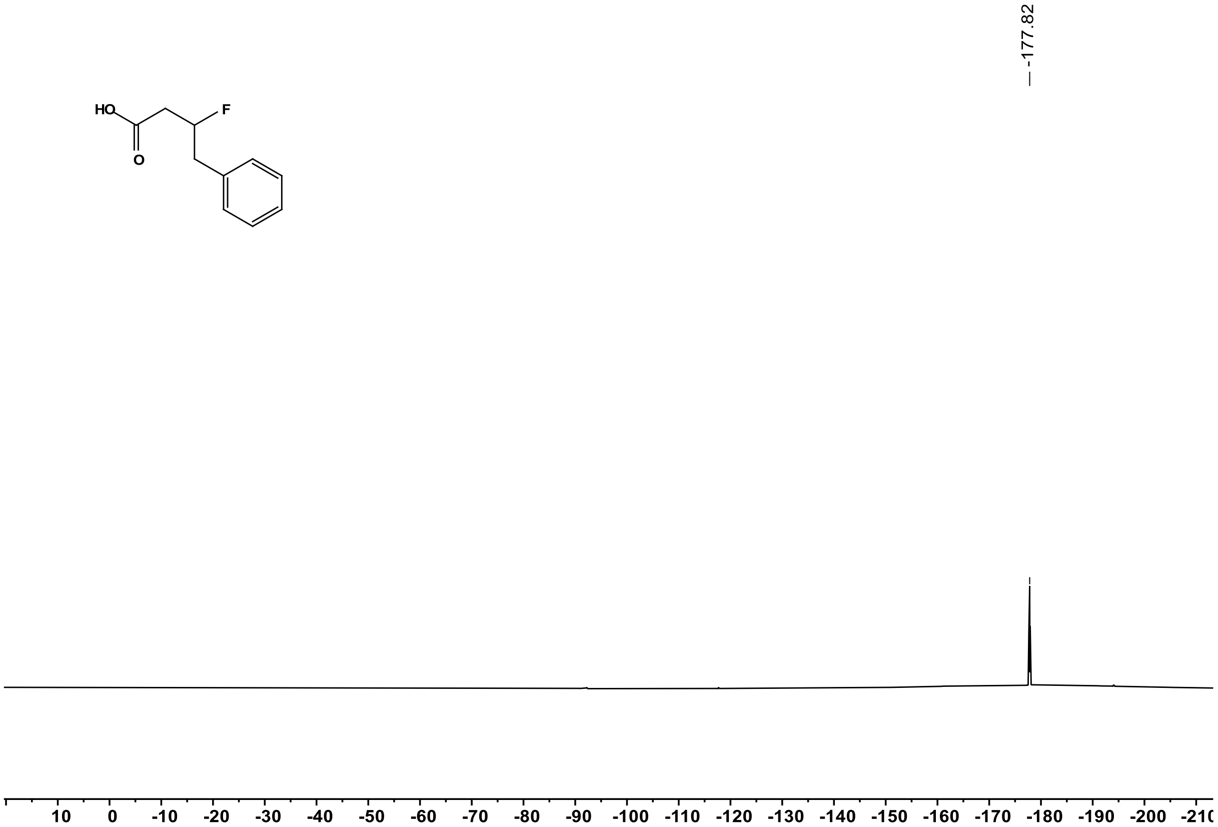

^{19}F NMR spectrum of compound **2u** (282 MHz, $CDCl_3$)

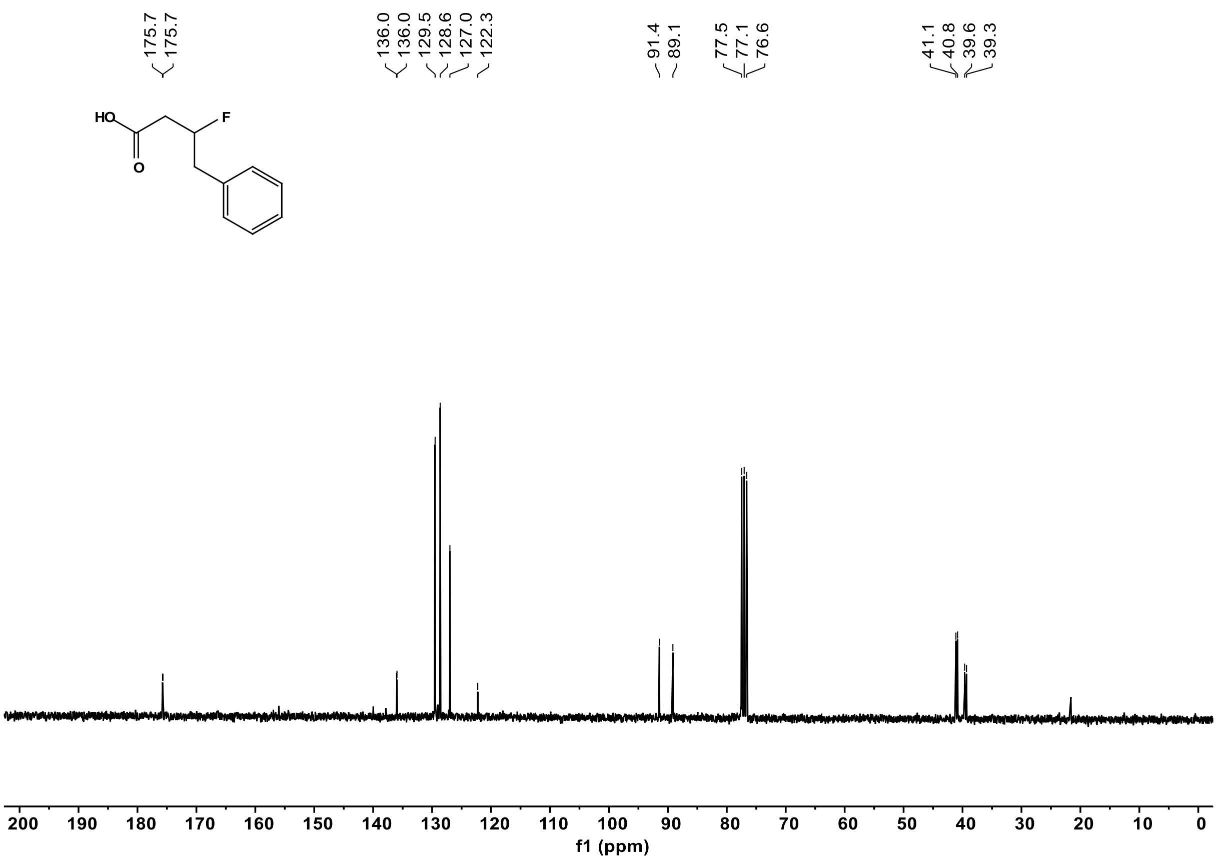

$^{13}C\{^1H\}$ NMR spectrum of compound **2u** (75 MHz, $CDCl_3$)

7. References

(1) Zhang, Y.; Qian, J.; Wang, M.; Huang, Y.; Hu, P. Visible-Light-Induced Decarboxylative Fluorination of Aliphatic Carboxylic Acids Catalyzed by Iron. *Org. Lett* **2022**, *24* (32), 5972-5976.

(2) Mizuta, S.; Stenhagen, I. S. R.; O'Duill, M.; Wolstenhulme, J.; KirJavainen, A. K.; Forsback, S. J.; Tredwell, M.; Sandford, G.; Moore, P. R.; Huiban, M.; et al. Catalytic Decarboxylative Fluorination for the Synthesis of Tri- and Difluoromethyl Arenes. *Org. Lett* **2013**, *15* (11), 2648-2651.

(3) Wu, X.; Meng, C.; Yuan, X.; Jia, X.; Qian, X.; Ye, J. Transition-metal-free visible-light photoredox catalysis at room-temperature for decarboxylative fluorination of aliphatic carboxylic acids by organic dyes. *Chem Commun (Camb)* **2015**, *51* (59), 11864-11867.

(4) Huang, X.; Liu, W.; Hooker, J. M.; Groves, J. T. Targeted Fluorination with the Fluoride Ion by Manganese-Catalyzed Decarboxylation. *Angew. Chem. Int. Ed* **2015**, *54* (17), 5241-5245.

(5) Yedase, G. S.; Kumar, S.; Stahl, J.; König, B.; Yatham, V. R. Cerium-photocatalyzed aerobic oxidation of benzylic alcohols to aldehydes and ketones. *Beilstein J. Org. Chem* **2021**, *17*, 1727-1732.
